# Dietary vegetable oils do not alter the intestine transcriptome of gilthead sea bream (*Sparus aurata*), but modulate the transcriptomic response to infection with *Enteromyxum leei*

**DOI:** 10.1186/1471-2164-13-470

**Published:** 2012-09-11

**Authors:** Josep A Calduch-Giner, Ariadna Sitjà-Bobadilla, Grace C Davey, Michael T Cairns, Sadasivam Kaushik, Jaume Pérez-Sánchez

**Affiliations:** 1 Nutrigenomics and Fish Growth Endocrinology Group, Department of Marine Species Biology, Culture and Pathology, Instituto de Acuicultura Torre de la Sal (IATS-CSIC), Ribera de Cabanes, Castellón, 12595, Spain; 2 Fish Pathology Group, Department of Marine Species Biology, Culture and Pathology, Instituto de Acuicultura Torre de la Sal (IATS-CSIC), Ribera de Cabanes, Castellón, 12595, Spain; 3 Ryan Institute, National University of Ireland, Galway, Ireland; 4 INRA, UR1067 NuMeA Nutrition, Metabolism Aquaculture, Saint Pée-sur, Nivelle, F64310, France

**Keywords:** Teleost, Parasite, Myxozoa, Intestine, Transcriptome, Nutrigenomics

## Abstract

**Background:**

Studies conducted with gilthead sea bream (*Sparus aurata* L.) have determined the maximum dietary replacement of fish meal and oil without compromising growth or product quality. The present study aimed to analyze the effect of the nutritional background on fish health and fish fed plant protein-based diets with fish oil (FO diet) or a blend of vegetable oils (66VO diet) were exposed for 102 days to the intestinal myxosporean parasite *Enteromyxum leei*, and the intestine transcriptome was analyzed with a customized oligo-microarray of 7,500 annotated genes.

**Results:**

Infection prevalence was high and similar in the two diet groups, but the outcome of the disease was more pronounced in fish fed the 66VO diet. No differences were found in the transcriptome of both diet control groups, whereas the number of differentially expressed genes in infected groups was considerable. K-means clustering of these differentially expressed genes identified four expression patterns that reflected the progression of the disease with the magnitude of the fold-change being higher in infected 66VO fish. A positive correlation was found between the time of infection and the magnitude of the transcriptional change within the 66VO group, being higher in early infected animals. Within this diet group, a strong up-regulation of many components of the immune specific response was evidenced, whereas other genes related to complement response and xenobiotic metabolism were down-regulated.

**Conclusions:**

The high replacement of fish oil by vegetable oils in practical fish feeds did not modify the intestine transcriptome of gilthead sea bream, but important changes were apparent when fish were exposed to the myxosporean *E. leei*. The detected changes were mostly a consequence rather than a cause of the different disease progression in the two diet groups. Hence, the developed microarray constitutes an excellent diagnostic tool to address changes associated with the action of intestinal pathogens, but lacks a prognostic value to predict in advance the different susceptibility of growing fish to the current pathogen.

## Background

The increased demand for fish meal and fish oil to meet the requirements of expanding aquaculture and other farming industries has led to the search for alternative raw materials. Attention has been focused on plant ingredients and studies conducted in gilthead sea bream (GSB) (*Sparus aurata* L.) have demonstrated that the combined replacement of fish meal and fish oil (FO) is highly feasible without detrimental effects in growth performance when the theoretical requirements for essential amino acids and fatty acids are met by the diet [1,2]. However, obvious changes in muscle fatty acid (FA) signatures occur with the replacement of FO by vegetable oils (VO) [3-5] and a wash-out period with fish oil-based diets is needed for the restoration of the desired FA profile having a high concentration of n-3 long-chain polyunsaturated FAs [6].

The effect of the nutritional background on fish health and welfare also merits careful consideration and there is much interest for research in this area in the recent years (reviewed in [7]). Indeed, adequate nutrition is essential to maintain health and to reduce disease susceptibility and pathological changes, and dietary lipids, as other nutritional factors, have specific actions on the immune response [8,9]. In GSB, it is noteworthy that the redox balance [10] and the cortisol response after stress confinement [11] were altered by the high replacement of FO by VOs. Even lower VO replacement levels (50-60%) increased the cumulative mortality in GSB challenged with *Vibrio alginolyticus* or increased the intestine expression of TNF-α in *Photobacterium damselae* subsp. *piscicida* injected animals (see [9]). Furthermore, fish fed with a diet rich in VO exhibited a worse disease outcome when challenged with the intestinal parasite *Enteromyxum leei*, consisting in lower biometrical (weight, condition factor, specific growth rate), immunological (haematocrit, complement and lysozyme activity) and antioxidant (hepatic total glutathione) parameters compared to infected fish fed a FO diet [12]. In an effort to understand the possible underlying mechanisms involved in this greater progression of the infection in VO fish, we undertook a series of detailed studies of gut immunology and physiology in fish fed plant- or fish-based diets and challenged with this myxosporean. This intestinal parasite causes severe desquamative enteritis and thus far there are no preventive or curative treatments for this enteromyxosis. However, a few select inflammatory and immune relevant genes have been initially explored [13,14], and more recently the molecular profiling with a cDNA microarray of the host response to the chronic exposure to *E. leei* has highlighted a complex interplay of proteases, protease inhibitors, apoptotic factors as well as cell proliferation and antioxidant defence genes [15]. The same array has also proven to be very useful for assessing the time course of stress response after confinement exposure [16], and herein the transcriptome database which served for the construction of these arrays was further enriched by suppression subtractive hybridization (SSH) libraries with lipid-responsive genes. All sequences were *de novo* assembled and the final annotated sequences were used to construct a customized oligo-array for expression profiling of the GSB intestine using a factorial design (2x2) with diet composition and parasite infection as experimental variables. To pursue this issue, juvenile GSB fed plant protein-based diets with either FO diet or a blend of VOs (66VO diet) as a major dietary lipid source were infected with the intestinal parasite *E. leei* by water effluent and the intestinal transcriptome of the host was analyzed 102 days post-challenge.

## Results

### Microarray profiling

**Figure 1:**
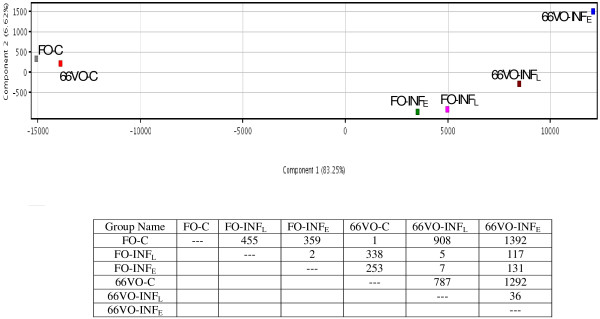
**Principal components analysis of gut transcriptome after nutritional and parasite challenges.** The number of differentially expressed genes among experimental groups was determined by one-way ANOVA (corrected P-value < 0.05, Benjamini-Hochberg).

Principal component analysis of microarray results revealed that the first two components accounted for the 89% of total variation (Figure1). Much of the variation (83%) was explained by component 1, which primarily separated three main groups according to the progression of infection: i) control fish fed FO (FO-C) and 66VO diets (66VO-C), ii) FO-recipients with early (FO-INF_E_) or late (FO-INF_E_) infection, and iii) 66VO-recipients with early (66VO-INF_E_) or late (66VO-INF_E_) infection. More than 2,000 unique sequences were differentially expressed when comparisons were made among all groups (corrected P-value < 0.05, Benjamini-Hochberg), but interestingly only one gene was differentially expressed when comparisons were made between FO-C and 66VO-C groups. Among the differentially expressed genes, 110 were derived from the SSH libraries enriched with lipid-responsive genes.

**Figure 2:**
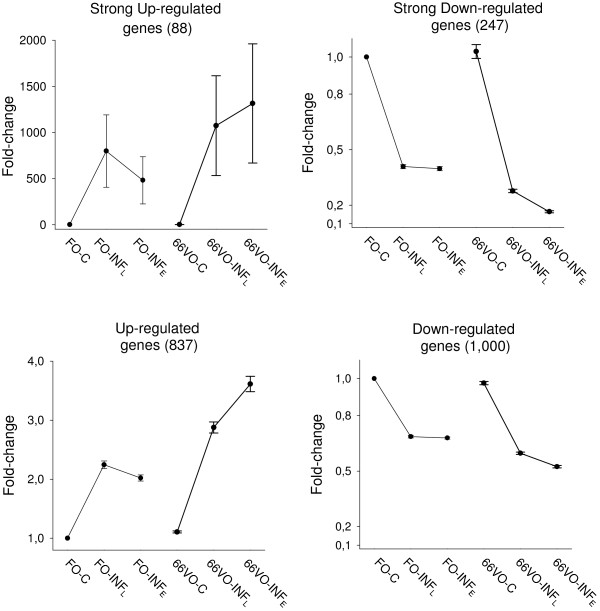
**K-means analysis of differentially expressed genes in the diet groups after parasite challenge.** The FO-C group was used as reference for fold-change calculations. Values are the mean ± SEM. For each cluster group, the number of differentially expressed genes is in parenthesis.

The k-means clustering of differentially expressed genes identified four major expression patterns or clusters (Figure2). The entire sets of genes included in each cluster are listed in an additional file (Additional file 1: Table S1) with fold-change expression values referred to the FO-C group. Cluster 1 was composed of 88 genes that were strongly up-regulated in early and late infected fish (fold-change > 500). Gene Ontology (GO) and Fisher enrichment analyses did not show relevant GO categories for cluster 1 that included, among others, carbonic anhydrase 9, arginase-1 and many proteases and ribosomal proteins. A second group of up-regulated genes (fold-change 2.0-4.0) were grouped in cluster 2 (838 genes) that was significantly enriched (P < 0.05) in GO terms related to “translation”, “RNA processing”, “mitotic cell cycle” and “cell cycle” with a high representation of urea cycle and polyamine biosynthetic pathway genes (ornithine aminotransferase, ornithine carbamoyltranferase, ornithine decarboxylase), immunoglobulins and cytokines, including interleukin-6 (IL6), IL6 receptor and interferon-related proteins.

Strongly and moderately down-regulated genes were included in cluster 3 and 4, respectively. Cluster 3 contained 247 genes (fold-change 0.1-0.4) enriched in the GO categories “oxidation-reduction” and “cellular lipid metabolic process” (P < 0.05). In particular, P450 detoxifying enzymes, phospholipase A2, intestinal fatty acid binding protein (FABP2), uncoupling protein 1 (UCP1) and many complement related factors, such as complement C1q tumor necrosis factor-related protein 3, complement factor H, complement factor C2, lectin and fucolectin-1. Cluster 4 was the most abundant group with 1,000 differentially expressed genes that exhibited a moderate down-regulation (fold-change 0.5-0.7) in response to infection. This cluster was significantly enriched (P < 0.05) in genes with the GO categories “signal transduction”, “positive regulation of cellular process”, “cell differentiation” and “regulation of RNA metabolic process”, and included among others several growth factors such as insulin-like growth factor II (IGF-II), growth hormone receptors type I (GHR-I) and II (GHR-II) and IGF binding-protein 4 (IGFBP4).

**Figure 3:**
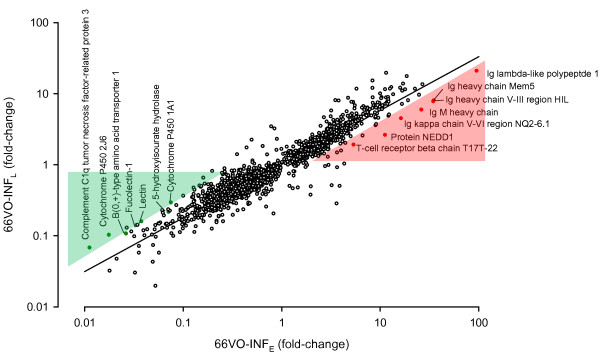
**Correlation of the gene expression pattern for differentially expressed genes of clusters 2, 3 and 4 in early (66VO-INF**_**E**_**, X-axis) and late infected fish (66VO-INF**_**L**_**, Y-axis) fed the 66VO diet.** The FO-C group was used as reference for fold-change calculations. Shaded areas mark genes that are particularly up- (red) or down-regulated (green) after prolonged time of infection.

As a general rule, the k-means cluster analysis also showed that the magnitude of change for differentially expressed genes was highest in *E. leei*-challenged fish in the 66VO group, which also exhibited a higher and faster disease progression (for details see [12]). In addition, within the 66VO group, the magnitude of transcriptional change was more pronounced in early infected fish (66VO-INF_E_) than in those infected later (66VO-INF_E_). This was particularly evident when differentially expressed genes (clusters 2, 3 and 4) in early infected fish (66VO-INF_E,_ X-axis) were plotted against the gene expression profile of late infected fish (66VO-INF_L,_ Y-axis), evidencing a very good linear correlation (r = 0.95) (Figure3). However, the detailed analysis of the correlation showed that some genes deviate from the predictions (shaded areas in Figure3). This was the case for genes related to the production of immunoglobulins, which were more over-expressed than envisaged in early-infected 66VO fish, whereas those related with the complement pathway (lectin, fucolectin-1, complement C1q tumor necrosis factor-related protein 3) and the metabolism of xenobiotics (cytochrome P450 1A1, cytochrome P450 2J6) were more under-expressed than expected. This means that some genes are particularly up- or down-regulated after prolonged time of infection.

### Functional annotation

**Figure 4:**
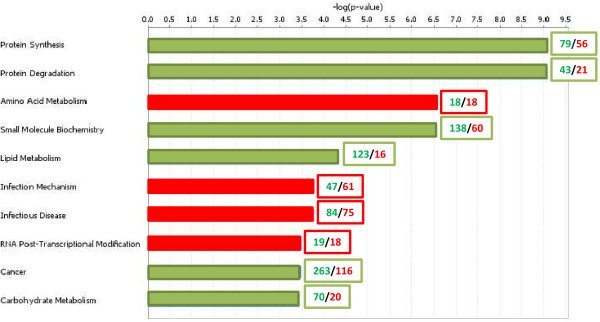
**Over-represented biological functions in differentially expressed genes in early infected fish fed the 66VO diet (66VO-INF**_**E**_**) as determined by IPA (Fisher’s Exact test, P < 0.05).** For each biological function, column colours represent predominance of down-regulated genes (green) or a balanced distribution between up- and down-regulated genes (red). The numbers of down-regulated (green) and up-regulated (red) genes are represented in boxes.

Given that the major disease outcome was observed in 66VO-INF_E_ fish, pathway analysis by means of IPA software focused on this group (Figure4). For the top ten biological functions, more than 65% were represented by down-regulated genes (green bars), which was the case for protein synthesis, protein degradation, small molecule biochemistry, lipid metabolism, cancer and carbohydrate metabolism. By contrast, other biological functions, including amino acid metabolism, RNA post-transcriptional modification, infection mechanism and infectious disease were mostly equally or slightly over-represented in the group of up-regulated genes (red bars).

### Real time qPCR validation

**Figure 5:**
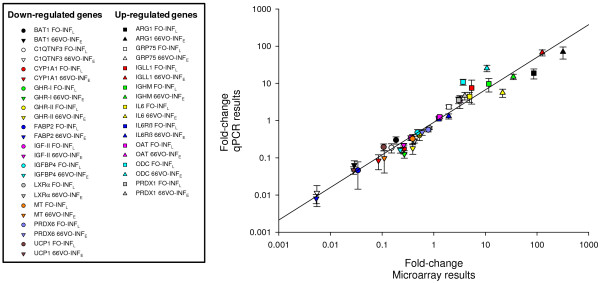
**Real-time qPCR validation of microarray results.** Correlation plot of fold-change values for the selected genes analyzed by microarray (X-axis) and qPCR methodologies (Y-axis) in infected fish fed FO and 66VO diets. The FO-C group was used as reference for fold-change calculations. To simplify the graph representation mean deviations (mean ± SEM) are only represented for qPCR data.

Microarray results were validated for a large number of differentially expressed genes in fish from each diet group (Figure5). There was a strong linear correlation between microarray and qPCR results, though genes with the highest level of expression (e.g., arginase-1 and immunoglobulin lambda-like polypeptide 1) were slightly over-estimated in the microarray in comparison to the results of qPCR.

## Discussion

To date, a considerable number of studies have been conducted in fish to identify target genes of relevance for the improvement of production traits of farmed fish species (reviewed in [17-19]). The liver, due to its major role in metabolism, has been the main studied tissue in fish genome-wide analyses with special attention to stress response [16,20,21], ecotoxicology [22-24] or nutrigenomics [25-28]. Transcriptome responses involving the fish immune system after bacterial or viral experimental challenges have also been assessed in liver, although the main studied tissue for this purpose is the head kidney because of its central hematopoietic role, equivalent to that of mammalian bone marrow [29-31]. The fish intestine, besides its importance for the absorption of nutrients and osmoregulation, also acts as an immune tissue and constitutes a barrier and first line of defence against certain pathogens and environmental challenges [32,33], so the gene expression profile of the intestine in nutritional and/or pathogenic trials should be specially considered. However, very few transcriptome-wide studies have paid attention to the response of fish gut, being focused these approaches on salinity adaptation in European eel (*Anguilla anguilla*) [34], and phosphorus deficiencies and dietary immunostimulants in rainbow trout (*Oncorhynchus mykiss*) [35,36] or replacement of fish meal and FO in diets for Atlantic halibut (*Hippoglossus hippoglossus*), Atlantic salmon (*Salmo salar*), and Atlantic cod (*Gadus morhua*) [37-39].

In GSB, the host response to chronic exposure to *E. leei* was previously analyzed in both intestine and head kidney by transcriptome profiling of few candidate genes [14], and more recently [15] by means of a cDNA microarray that was previously proven successful to assess the response of the liver of fish undergoing confinement exposure [16]. The oligo-microarray designed and used in the current work was developed from these previous annotated nucleotide sequences updated with lipid-responsive genes by means of a SSH approach in order to have a wide range of genes potentially regulated by nutritional deficiencies in essential fatty acids. In this scenario, it must be noted that this is the first attempt to analyze the combined effect of diet and parasite infection on the fish intestinal transcriptome. The detected massive transcriptome changes can be mainly attributed to the progression of the infection, but not to the dietary treatment, since no differences between diet groups were found in the intestine transcriptome of control animals (not exposed to the parasite) as revealed by the one-way ANOVA and principal components analysis. Furthermore, both control diet groups showed similar growth performance with no evidences of hepatic and intestinal histopathology, probably due to the adequate supplementation of diets with soy-lecithin as an extra-source of dietary phospholipids [2,3]. By contrast, in Atlantic salmon and Atlantic cod, the adverse effects of dietary soybean meal leading to intestinal disorders (inflammation and lipid accumulation) were also accompanied by changes in the intestine transcriptome [38,39].

In our experimental model, intestine transcriptomic differences between diets only became evident when animals were infected with the parasite, as the number of differentially expressed genes and the degree of fold-change variations were much higher in animals fed the 66VO diet than in those fed the FO diet, which paralleled the increased symptoms of enteromyxosis (lower growth, condition factor and haematocrit in combination with higher anorexia, and intensity and extension of the infection) in parasitized fish of this diet group [12]. Furthermore, the gene expression profile determined by k-means clustering also emphasizes the importance of the progression of the infection, with a stronger effect in terms of fold-change variation in infected animals fed the 66VO diet, and even more in early infected fish. This reinforces the idea that the microarray used in the present study constitutes an excellent diagnostic tool to address changes associated with the action of the pathogen.

When analyzing the biological functions of the genes differentially expressed upon infection, it was evident that the infection produced a detrimental effect on many pathways related to growth and normal metabolism, as genes related to protein synthesis, protein degradation, small molecule biochemistry, lipid metabolism, cancer and carbohydrate metabolism were down-regulated. By contrast, other biological functions, related to infection mechanism, infectious disease and immune response were over-represented by up-regulated genes. It is difficult to compare the current results with those obtained in other fish-pathogen models, since the host-pathogen interactions are different and the times post-infection at which the samples are analyzed differ. However, in most cases up-regulation of different immune genes occurred and the down-regulation of many other genes was also evident, as already shown [31].

In order to define the response to the parasite, special attention was paid to differentially expressed genes (up- and down-regulated genes) of biological processes statistically enriched in the infected groups, and the reliability of the results was validated by qPCR of selected key genes representative of the four clusters. The enzyme arginase-1 was representative of the strongly up-regulated genes of cluster 1 in infected fish. Enhanced expression of this gene was also detected in the head kidney of Atlantic salmon and common carp (*Cyprinus carpio*) challenged with the bacterium *Aeromonas salmonicida* and the protozoan parasite *Trypanosoma carasii*, respectively [30,40]. The so-called “alternatively” activated macrophages play important roles in the clearance of pathogens (as reviewed in [41]), and their enhanced arginase activity allows them to produce ornithine, a precursor of hydroxyproline and polyamines. A first enzyme step is that of ornithine decarboxylase (ODC), though ornithine can also be produced from glutamate via ornithine aminotransferase (OAT). Interestingly, in our experimental model both ODC and OAT were up-regulated and belong to cluster 2, which confirms and extend the idea of an enhanced production of polyamines in the fish challenged with *E. leei*.

Polyamines have a significant effect on the growth of the gastrointestinal mucosa of a variety of organisms including fish [42], and the *in vitro* immunostimulatory action of putrescine has been observed in head kidney leukocytes of GSB [43]. Therefore, the increased expression of these genes involved in cell proliferation in infected GSB should be interpreted as an evidence of the regenerative action of the intestinal tissue as a response to the damage induced by the parasite invasion. L-arginine is also the substrate for the synthesis of nitric oxide (NO) by the catalytic action of NO synthases. Thus, if L-arginine is mainly used by arginase-1 in ornithine synthesis, the production of NO should be expected to be reduced, and this is exactly what occurred in the serum NO levels of infected fish [12]. NO is an important molecule in regulating immune functions and also has a direct antimicrobial effect [44]. However, NO has double-edge sword effects [45], since an elevated production of NO for long periods of time not only can have the desirable protective action against the pathogen, but also can lead to a higher concentration of NO available to react with O_2_, increasing the production of reactive nitrogen species (RNS) and generating indirect toxic effects on the host. Since reactive oxygen species (ROS) were enhanced in infected fish (increased respiratory burst of circulating leukocytes) [12], it is a reasonable conservative strategy to reduce RNS to avoid detrimental effects to the host.

In cluster 2, several molecular chaperones (mitochondrial 10 kDa and 60 kDa heat shock proteins, glucose-regulated protein 75, heat shock 70 kDa protein 4, mitochondrial chaperone BCS1, proteasome assembly chaperone 3) were up-regulated in the infected fish. The role of these life essential proteins is to stabilize unfolded proteins, often coupling ATP binding/hydrolysis to the folding process. Thus, their expression is often increased by cellular stress, as occurs with heat shock proteins of the HSP70 family, which are highly inducible under stress conditions in higher vertebrates [46] and also in fish [47]. Glucose-regulated protein 75, also named mortalin or mitochondrial HSP70, is one of the molecular chaperones representative of this cluster and their enhanced expression was coincident with the up-regulation of several mitochondrial ATP synthases, included in the same cluster. This finding is consistent with previous results analyzing the gene expression pattern of some target growth, redox and immune-relevant genes in the intestine of GSB [14]. The up-regulated expression of mortalin at the mRNA and protein level has also been observed in the liver tissue of GSB during both acute and chronic confinement stress [48], which emphasizes the relevance of this mitochondrial protein encoded by nuclear DNA as a stress biomarker in this fish species. Interestingly, no changes in mortalin expression were observed in the gills of Atlantic salmon infected with the protozoan *Neoparamoeba perurans*, the causative agent of amoebic gill disease, but resistant animals exposed but not infected with the parasite showed a significant up-regulation of mortalin expression [49], which evidences a complex and perhaps species-specific protective role of this protein in front of different stressors. Cluster 2 also comprised several immune-related genes, and the expression pattern of some of them, including IL6, IL6 receptor and peroxiredoxin 1 (also named natural killer enhancing factor-A), was validated by qPCR. Their up-regulation probably reflects the activation of innate immune response at the local site of infection. Similarly, in other fish-parasite models, up-regulation of different immune genes, mainly chemokines, cytokines, lectins and enzymes of eicosanoid metabolism, have been reported at the local site of infection (gills, skin, cartilage) [50-55].

Cluster 3 grouped genes severely down-regulated by infection. Among them, genes involved in xenobiotic metabolism and detoxification pathways were represented by cytochromes P450 and metallothionein. Since an increasing body of evidence points to the importance of intestine as a xenobiotic-metabolizing tissue [56], this decreased gene expression should be viewed as a counter-regulatory response that maintains the redox balance between the mechanisms bringing about the pathogen elimination and those governing the growth and repair of damaged tissues in a scenario where ROS production is potentiated in response to the parasite [12]. Another group of genes related to lipid metabolism (FABP2, liver X receptor alpha, phospholipase A2) and other established markers of metabolic activity in GSB, such as UCP1 and peroxiredoxin 6 [57,58], were also down-regulated in cluster 3. Their reduced expression with infection is also suggestive of the loss of intestinal functions. This was further corroborated by pathway analysis of the differentially expressed genes during infection and by the down-regulation in cluster 4 of components of the somatotropic axis (GHR-I, GHR-II, IGF-II, IGFBP4) of importance in intestinal growth and repair [59], that can be considered a prelude to the severe cachectic episodes typically associated with more advanced stages of *E. leei* infection.

Some genes stand out of the general trend of higher regulation (either up or down) with longer infection time, as shown in Figure3. This was the case for immunoglobulins, whose production was more up-regulated than expected in early-infected fish. This is consistent with the fact that the specific immune response takes more time to appear in fish than in higher vertebrates [60] and this time is particularly extended in this fish-parasite model. In fact, it has been shown that time of exposure to *E. leei* is the most determinant factor for the intestine expression of immunoglobulin M, the major component of fish specific humoral response, and this response is also magnified in 66VO fish [61]. In any case, we cannot discard the possible action of another Ig isotype, IgT/IgZ, which seems to act exclusively in mucosal areas, and has been described in very few fish species, with outstanding results in another myxosporean infection [62], but not yet found in GSB nor present in the current microarray. On the other hand, some genes related to complement pathways were strongly down-regulated, which agrees with the previously observed down-regulation in *E. leei*-exposed GSB, although timing of infection was not considered [15]. These observations reflect the exhaustion of the alternative complement pathway also reported in other *Enteromyxum* spp. chronic infections [13,63,64].

Taking together all these results, it appears that the changes detected in the intestine transcriptome are mostly a consequence rather than a cause of the different disease progression, so the differentially expressed genes can function as diagnostic markers of disease progression but lack a prognostic value to predict in advance the susceptibility of diet groups to an infective scenario. Although we did not find any transcriptomic differences in the intestine of the control groups through this microarray approach, other possibilities rather than a lack of effect of a differential diet on the intestinal gene expression profile must be considered to explain this result: (i) technical limitation of our microarray to detect significant differences in the expression of some genes, (ii) absence of genes with potential prognostic value in our nucleotide database or the resulting microarrays, or (iii) differences between groups could be due to non-transcriptional mediated processes. Further work is under way in order to achieve a more complete picture of the transcriptome of GSB. For instance, next-generation sequencing techniques (454 pyrosequencing) have been applied to normalized libraries from several GSB tissues including intestine, yielding millions of new nucleotide reads that will update and enrich in thousands of genes our GSB nucleotide database. Thus, the potential for the detection of changes in the expression of new genes will be greatly increased in future studies.

Finally, in this and other related works where the fish transcriptome response to an infective pathogen is observed, the transcriptome response is focused exclusively on the host [65-67], but the direct effects of diet composition and nutrients on parasite physiology and survival should also be considered, as it has been in the case of the direct effect of FAs as antimalarial agents where a direct effect on the FA biosynthetic machinery of the parasite *Plasmodium falciparum* has been shown [68]. It must be noted that most oils of vegetable origin are less susceptible to oxidative degradation than fish oils due to their lower content of very-long-chain *n*-3 polyunsaturated fatty acids [69], so parasite fatty acid uptake when feeding with FO diet could make it more sensitive to oxidative stress and consequently to the immune host response.

## Conclusions

Plant oils in plant protein-based diets did not modify the intestine transcriptome of GSB, although there were significant effects when these fish were exposed to a parasite challenge with the myxosporean *E. leei*. The detected changes are mostly a consequence rather than a cause of the different disease progression in the two diet groups. The microarray approach as used here constitutes an excellent diagnostic tool to address changes associated with the action of intestinal pathogens, but lacks a prognostic value to predict in advance the susceptibility of different diet groups to this pathogen. Further studies are needed to explore the mechanisms involved in the altered susceptibility of GSB against the parasite *E. leei* when fed different diets, without excluding direct effects of fish feeds on parasite metabolism.

## Methods

### Experimental design

Details of the experimental design and sampling procedure have been provided previously [12]. Briefly, naïve juvenile fish were checked for the absence of the parasite and divided in two experimental groups fed over 9 months two different diets Additional file ( 2: Table S2) with either fish oil (FO diet) or a blend of vegetable oils (66VO diet, 66% fish oil replacement) as the major source of dietary lipids. After this period, fish from both diet groups were exposed to *E. leei*-water effluent (recipient group, R, n = 30) or kept unexposed (control group, C, n = 30). All fish were individually tagged with passive integrated transponders and non-lethally sampled at three consecutive times (32, 53, 88 days) for parasite diagnosis. In a final lethal sampling at 102 days post exposure (p.e.), fish were euthanized for tissue sampling and posterior intestine was rapidly excised, frozen in liquid nitrogen and stored at -80°C. All procedures were carried out according to national (CSIC, Institute of Aquaculture Torre de la Sal Review Board) and current EU legislation on the handling of experimental animals.

### Parasite diagnosis

Parasite diagnosis was performed by PCR from non-lethal samples obtained at different times (32, 53, 88 days) post-challenge by probing the rectum with a cotton swab as described in [70]. At the final lethal sampling (102 days), parasite diagnosis was performed in intestine samples by observation of histological sections following standard procedures (haematoxylin and eosin staining on paraffin-embedded sections). More details in [12].

As fish are infected by water effluent, the kinetics of the infection may differ in each individual. Thus, fish tagging and non-lethal diagnosis allowed the individualized monitoring of infection along the experimental period and the classification of R fish according to their first infection-timing in two categories: early infected (FO-INF_E_ and 66VO-INF_E_ groups), being infected at 32 or 53 days p.e., and late infected (FO-INF_E_ and 66VO-INF_E_ groups), being infected at 88 days p.e. or later. Early infected fish had higher intensity of infection and extension of the infection than late infected ones. Fish non-exposed to the parasite (FO-C and 66VO-C groups) remained uninfected throughout the experiment.

### Construction of SSH libraries

Total RNA was extracted using a Qiazol and RNeasy Maxi combination protocol (Qiagen) from liver and adipose tissue of fish fed FO and VO with signs of essential FA deficiencies (total fish oil replacement) [1]. Transcripts of mRNA were purified from total RNA using an Illustra Quickprep Micro mRNA purification kit (GE Healthcare). The mRNA populations were quantified by spectrophotometric measurements at 260 nm and analyzed for quality by the Agilent 2100 bioanalyzer (Agilent). Construction of SSH libraries was performed by means of the BD PCR Select cDNA Subtraction Kit (BD Biosciences Clontech). Briefly, two μg of mRNA were used to generate tester and driver cDNA. Then, both cDNAs were digested with *Rsa* I, tester cDNA was ligated to adaptor, and tester and driver cDNA were hybridized and PCR amplified. Aliquots (1.5 μl) of secondary PCR products from subtracted cDNA populations were ligated to the pCR2.1 vector using the TA Cloning Kit (Invitrogen). Aliquots of ligation reactions were transformed into competent Top10 *E. coli* cells. Sequencing of SSH libraries (5,760 clones) was carried out at the Max Planck Institute of Molecular Genetics (Berlin, Germany), using ABI 3730XL (Applied Biosystems) and MegaBACE 4500 (GE Healthcare) capillary sequencer systems. All sequencing reactions were carried out with ABI BigDye Terminator version 3.1.

### Sequence assembly and annotation

The cDNA sequences from SSH libraries were edited to remove vector and adaptor sequences, cleaned and filtered before clustering and annotation by the SIGENAE platform (INRA Toulouse, France). Cleaning involved masking of poor quality bases and low complexity sequences such as polyA tails. Filtering removed contaminating sequences (bacteria, yeast) and only high quality sequences of more than 100 bases in length were retained and deposited in the NCBI GenBank database [accession numbers GW820255-GW824593]. After combination with public and private GSB nucleotide sequences, a total number of 42,411 sequences were assembled in 20,218 contigs and singletons that were hosted in an exclusive GSB nucleotide database http://www.sigenae.org/iats. The SSH sequences from the present study amounted to 1,242 unique sequences (463 of which were new GSB sequences) and BLASTX similarity searches with a cut-off E-value of 1e-5 automatically annotated 7,587 sequences. GO analysis of all annotated sequences was made by means of the Blast2GO software [71], and GO terms like “response to stress”, “lipid metabolic process” and “immune system process” were amongst the most prevalent GO categories (Additional file 3: Table S3).

### RNA extraction for microarray analysis

Total RNA was extracted from individual intestine samples with the ABI PRISM™ 6100 Nucleic Acid PrepStation (Applied Biosystems). Samples were homogenized at a concentration of 25 mg/ml with a guanidine-detergent lysis reagent. The reaction mixture was treated with protease K and RNA purification was achieved by passing the tissue lysate (0.4 ml) through a purification tray containing an application-specific membrane. Wash solutions containing DNase were applied and total RNA was eluted into a 96-well plate. The final RNA yield was 10-30 μg and RIN (RNA integrity number) measurements using the Agilent 2100 Bioanalyzer ranged between 8 and 10, which is indicative of clean and intact RNA.

### Microarray construction, hybridization and data analysis

A custom high-density oligo-microarray (8 x 15K) was designed and printed (eArray web tool, Agilent) to analyze the intestine transcriptome of each diet group. The array comprised 2 sets of 60-oligomer probes for 7,500 GSB annotated sequences. Total RNA (200 ng) from individual fish (n = 9 for each control group and n = 6 for each recipient group) were labelled with cyanine 3-CTP, and 1,000 ng of each labelled cRNA were hybridized to microarray slides that were analyzed with an Agilent G2565BA Microarray Scanner according to the manufacturer’s protocol. Data were extracted using the Agilent Feature Extraction Software 9.5.3, and they were deposited in the Gene Expression Omnibus (GEO) database under accession identifier GSE35633. Data analysis, including k-means clustering of differentially expressed genes, was carried out with the GeneSpring GX 11.5.1 software (Agilent). GO and Fisher enrichment analyses of k-means clusters were carried out by means of Blast2GO software. Pathway analysis of differentially expressed sequences was performed using the Ingenuity Pathway Analysis (IPA) software.

### Real-time qPCR validation

Up to 21 genes representative of the four k-means clusters of differentially expressed genes were validated on individual samples (n = 6-9) by real-time qPCR, using an iCycler IQ Real-time Detection System (Bio-Rad). Synthesis of cDNA was performed with the High-Capacity cDNA Archive Kit (Applied Biosystems) using random decamers. For this purpose, 500 ng total RNA were reverse transcribed in a final volume of 100 μl. RT reactions were incubated 10 min at 25°C and 2h at 37°C. Negative control reactions were incubated in the absence of reverse transcriptase. Diluted RT reactions were conveniently used for PCR reactions in 25 μl volume. Each PCR-well contained a SYBR Green Master Mix (Bio-Rad) and specific primers (Table1) were used at a final concentration of 0.9 μM. DNA Polymerase was activated and cDNA denatured by preincubation for 3 min at 95°C; the template was amplified for 40 cycles of denaturation for 15 s at 95°C, and annealing/extension at 60°C for 60 s. β-Actin was used as the housekeeping gene and the efficiency of PCR reactions for target and reference genes varied between 90% and 98%. The dynamic range of standard curves (serial dilutions of RT-PCR reactions) spanned five orders of magnitude, and the amount of product in a particular sample was determined by interpolation of the cycle threshold (Ct) value. The specificity of reaction was verified by analysis of melting curves and by electrophoresis and sequencing of PCR amplified products. Fluorescence data acquired during the extension phase were ultimately normalized to β-actin by the ΔΔCt method [72]. For each selected gene, fold-change variations were calculated for FO-INF_E_ and 66VO-INF_E_ using expression values of FO-C group as reference. 

**Table 1:** Primer sequences for real-time qPCR validation

**Gene name**	**Symbol**	**Primer sequence**
Arginase-1	ARG1	F CGT CCA GTC CAC AGT CAG CAC
		R TCG GGC AGG CGG TAG TCC
B(0,+)-type amino acid transporter 1	BAT1	F GCC GTG TGT GCT TTG TTG CTG
		R GGT GAA GAT AAG GGC TGG AGA TGG GGT GAA
Complement C1q tumor necrosis factor-related protein 3	C1QTNF3	F ATG CTG TGC TGA GAG AGA TGA G
		R AGT CTT CTG CTT CTC CTG CTC
Cytochrome P450 1A1	CYP1A1	F GCA TCA ACG ACC GCT TCA ACG C
		R CCT ACA ACC TTC TCA TCC GAC ATC TGG GGT GAA
Fatty acid-binding protein, intestinal	FABP2	F CGA GCA CAT TCC GCA CCA AAG
		R CCC ACG CAC CCG AGA CTT C
Glucose regulated protein 75	GRP75	F TCC GGT GTG GAT CTG ACC AAA GAC GGT GAA
		R TGT TTA GGC CCA GAA GCA TCC ATG GGT GAA
Growth hormone receptor type I	GHR-I	F ACC TGT CAG CCA CCA CAT GA
		R TCG TGC AGA TCT GGG TCG TA
Growth hormone receptor type II	GHR-II	F GAG TGA ACC CGG CCT GAC AG
		R GCG GTG GTA TCT GAT TCA TGG T
Immunoglobulin lambda-like polypeptide 1	IGLL1	F TGA GTG GTG TGA CGG TGG TG
		R ATG GTG GCT GTC TCT CCT TTG G
Immunoglobulin M heavy chain	IGHM	F ACC TCA GCG TCC TTC AGT GTT TAT GAT GCC GGT GAA
		R CAG CGT CGT CGT CAA CAA GCC AAG C GGT GAA
Insulin-like growth factor-II	IGF-II	F TGG GAT CGT AGA GGA GTG TTG T
		R CTG TAG AGA GGT GGC CGA CA
Insulin-like growth factor-binding protein 4	IGFBP4	F GGC ATC AAA CAC CCG CAC AC
		R ATC CAC GCA CCA GCA CTT CC
Interleukin-6	IL6	F TCT TGA AGG TGG TGC TGG AAG TG
		R AAG GAC AAT CTG CTG GAA GTG AGG
Interleukin-6 receptor subunit beta	IL6Rβ	F AGC ACT GAG TCT CCG TAT GAA GC
		R ACA ACT GAA ACC GCA TCT AAA GGC
Liver X receptor alpha	LXRα	F GCA CTT CGC CTC CAG GAC AAG
		R CAG TCT TCA CAC AGC CAC ATC AGG
Metallothionein	MT	F CTC TAA GAC TGG AAC CTG
		R GGG CAG CAT GAG CAG CAG
Ornithine aminotransferase	OAT	F TGC GGT CTG AGC TGA ACA A
		R CTT CCA GGC GTC GTA GTC T
Ornithine decarboxylase	ODC	F GCC TCG TGT CAC TCC CTT CTA TG
		R GCT GAA TCT CCG TCT TGC TTG C
Peroxiredoxin 1	PRDX1	F CTC CAA GCA ATA ATA AGC CCA AAG
		R TCA CTC TAC AGA CAA CAG AAC AC
Peroxiredoxin 6	PRDX6	F AGA GAC AAG GAC GGA ATG C
		R TGT GGC GAC CTT CTT CTG
Uncoupling protein 1	UCP1	F GCA CAC TAC CCA ACA TCA CAA G
		R CGC CGA ACG CAG AAA CAA AG
ß-Actin		F TCC TGC GGA ATC CAT GAG A
		R GAC GTC GCA CTT CAT GAT GCT

### Statistical analysis

Microarray results from the six experimental groups (FO-C, 66VO-C, FO-INF_E_, 66VO-INF_E_, FO-INF_E_, 66VO-INF_E_) were analyzed by one-way ANOVA (corrected P-value < 0.05, Benjamini-Hochberg) and principal components analysis by means of GeneSpring software.

## Competing interests

The authors declare that they have no competing interests.

## Authors' contributions

JPS, SK and ASB conceived and designed the study. ASB designed and performed the parasite infection and diagnosis. JACG, ASB and JPS sampled the experimental animals. GCD and MTC produced the SSH libraries. JACG and JPS designed the microarray and performed GeneSpring, statistical, gene ontology and pathway analyses. JACG validated microarray data by qPCR. JACG, ASB and JPS wrote the manuscript. All authors read and approved the final manuscript.

## Supplementary Material

Additional file 1 **Table S1.** Sets of differentially expressed genes composing clusters 1-4. For each gene, fold-change values of experimentally expressed groups referred to FO-C group are represented. Click here for file

Additional file 2 **Table S2.** Ingredients and chemical composition of experimental diets. Click here for file

Additional file 3 **Table S3.** Top biological functions (GO multilevel) represented on the gilthead sea bream oligo-microarray. Click here for file
