# Supplementary material for: Dietary vegetable oils do not alter the intestine transcriptome of gilthead sea bream (Sparus aurata), but modulate the transcriptomic response to infection with Enteromyxum leei
Source: BMC Genomics. 2012 Sep 11;13:470. doi: 10.1186/1471-2164-13-470 (PMC3444936; doi:10.1186/1471-2164-13-470)
Supplement: Additional file 1 — Table S1. Sets of differentially expressed genes composing clusters 1-4. For each gene, fold-change values of experimentally expressed groups referred to FO-C group are represented. [file 1471-2164-13-470-S1.pdf]

Cluster 1: Strong up-regulation (88 genes)

Putative Id		INF$_E$	INF$_L$
26S proteasome non-ATPase regulatory subunit 11	*FO*	41.13	62.27
	66VO	102.90	83.44
40S ribosomal protein S11	*FO*	45.09	67.26
	66VO	122.91	91.03
40S ribosomal protein S27	*FO*	8.97	15.84
	66VO	19.32	16.10
40S ribosomal protein S8	*FO*	82.09	140.69
	66VO	213.30	216.88
40S ribosomal protein SA	*FO*	125.57	236.35
	66VO	393.18	315.60
60S ribosomal protein L10	*FO*	579.16	1055.31
	66VO	1613.84	1325.46
60S ribosomal protein L13-3	*FO*	8.36	12.43
	66VO	13.97	10.93
60S ribosomal protein L18a	*FO*	10.26	21.49
	66VO	22.29	17.19
60S ribosomal protein L19	*FO*	9.96	9.55
	66VO	10.81	9.89
60S ribosomal protein L21	*FO*	87.63	163.73
	66VO	232.49	205.85
60S ribosomal protein L26-like 1	*FO*	23.58	52.50
	66VO	71.90	69.44
60S ribosomal protein L3	*FO*	1035.90	1980.62
	66VO	3025.22	2576.21
60S ribosomal protein L32	*FO*	580.11	1044.03
	66VO	1575.11	1368.88
60S ribosomal protein L34	*FO*	18.16	28.97
	66VO	33.04	31.08
60S ribosomal protein L36	*FO*	14.39	23.71
	66VO	28.39	23.98
60S ribosomal protein L37a	*FO*	483.18	958.68
	66VO	1439.39	1272.55
60S ribosomal protein L4	*FO*	171.05	284.94
	66VO	417.70	351.37
60S ribosomal protein L5	*FO*	485.48	1043.76
	66VO	1654.62	1375.17
60S ribosomal protein L7a	*FO*	7.23	13.23
	66VO	16.09	13.06
60S ribosomal protein L9	*FO*	135.48	281.56
	66VO	427.57	355.74
6-phosphogluconate dehydrogenase, decarboxylating	*FO*	765.56	1346.07
	66VO	2263.16	1667.39
Actin-2	*FO*	55.78	88.83
	66VO	143.07	118.76
ADP-ribosylation factor 1	*FO*	474.56	863.94
	66VO	1356.99	1080.89
Ammonium transporter Rh type C	*FO*	13.57	21.31
	66VO	17.51	13.50
Annexin A7	*FO*	40.14	86.37
	66VO	134.26	99.40
Annexin-B12	*FO*	21.71	37.13
	66VO	47.26	38.24
AP-1 complex subunit sigma-2	*FO*	37.31	81.33
	66VO	89.93	76.44
Arginase-1	*FO*	93.98	86.52

	66VO	318.84	166.03
Balbiani ring protein 3	*FO*	22248.56	34184.47
	66VO	56119.17	47085.65
Beta-glucuronidase	*FO*	58.89	103.64
	66VO	181.15	123.12
Calcyclin-binding protein	*FO*	10.02	16.33
	66VO	18.79	16.75
Calnexin homolog	*FO*	47.17	106.86
	66VO	154.92	115.34
Carbonic anhydrase 2	*FO*	10.33	19.64
	66VO	29.26	21.30
Carbonic anhydrase 9	*FO*	2723.81	4817.33
	66VO	8079.29	6490.07
Cathepsin K	*FO*	17.07	26.46
	66VO	30.26	30.80
Cathepsin L2	*FO*	9.32	14.00
	66VO	18.48	15.68
Cathepsin Z	*FO*	257.34	433.30
	66VO	723.25	603.90
Chk1 checkpoint-like protein	*FO*	14.91	16.51
	66VO	17.86	15.31
Cofilin-1	*FO*	8.77	14.04
	66VO	20.28	14.94
Coronin-2B	*FO*	54.68	85.10
	66VO	172.77	102.00
Cysteine proteinase 4	*FO*	2512.43	4326.05
	66VO	7296.68	5863.35
Dehydroascorbate reductase	*FO*	76.09	125.27
	66VO	226.54	215.25
Delta-1-pyrroline-5-carboxylate dehydrogenase, mitochondrial	*FO*	68.48	114.67
	66VO	220.92	178.86
Ef hand family protein	*FO*	76.97	142.00
	66VO	231.80	192.31
Enolase	*FO*	1123.64	2202.18
	66VO	3654.70	2815.66
Fibrillin-2	*FO*	612.53	1167.15
	66VO	2088.83	1514.42
Fructose-bisphosphate aldolase B	*FO*	6.04	12.67
	66VO	19.06	12.93
Fructose-bisphosphate aldolase C	*FO*	11.79	19.93
	66VO	25.20	21.54
Glutamate dehydrogenase	*FO*	302.01	509.01
	66VO	841.82	616.75
Glyceraldehyde-3-phosphate dehydrogenase	*FO*	14.33	17.88
	66VO	21.68	18.73
GTP-binding protein SAR1	*FO*	31.40	52.58
	66VO	90.03	61.99
Heat shock protein 83	*FO*	274.94	583.30
	66VO	1050.25	762.26
Heme oxygenase	*FO*	23.32	30.04
	66VO	41.69	42.00
Histidine-rich glycoprotein	*FO*	27.27	42.10
	66VO	54.89	50.43
Histone H3.2	*FO*	366.89	788.77
	66VO	1217.31	1073.26
Histone H4	*FO*	1162.57	2167.43
	66VO	3367.62	2879.18
Immunoglobulin lambda-like polypeptide 1	*FO*	20.59	5.44
	66VO	126.02	26.20

Iodothyronine deiodinase 3	*FO*	7.39	5.97
	66VO	52.07	13.90
Leukocyte cysteine proteinase inhibitor 1	*FO*	29.99	39.23
	66VO	111.58	58.76
Mitochondrial-processing peptidase subunit beta, mitochondrial	*FO*	27.11	47.15
	66VO	71.06	60.06
Moesin/ezrin/radixin homolog 2	*FO*	43.68	69.50
	66VO	127.09	91.18
NADH-cytochrome b5 reductase 1	*FO*	36.71	51.02
	66VO	83.82	65.85
Nascent polypeptide-associated complex subunit alpha-like protein 3	*FO*	366.41	694.28
	66VO	1147.83	949.49
Peptidyl-prolyl cis-trans isomerase, mitochondrial	*FO*	407.39	628.51
	66VO	959.00	806.94
Peroxiredoxin-1-like	*FO*	22.69	30.40
	66VO	43.83	37.02
Peroxiredoxin-4-like	*FO*	434.67	781.75
	66VO	1262.22	975.14
Phosphatidylinositol-4-phosphate 5-kinase type-1 beta	*FO*	149.76	173.06
	66VO	365.07	286.91
Plastin-2	*FO*	1260.67	2036.75
	66VO	3279.75	2611.91
Probable actin-related protein 2/3 complex subunit 2	*FO*	227.42	404.15
	66VO	700.62	564.09
Probable glutathione peroxidase 2	*FO*	171.16	298.56
	66VO	498.30	380.13
Probable phospholipase D F09G2.8	*FO*	7.28	12.66
	66VO	28.76	13.39
Protein preY, mitochondrial	*FO*	12.79	22.97
	66VO	28.52	24.68
Protein-glutamine gamma-glutamyltransferase K	*FO*	40.82	74.58
	66VO	153.88	115.26
Pumilio homolog 2	*FO*	227.69	403.21
	66VO	777.34	554.28
Rab GTPase-activating protein 1	*FO*	12.52	22.02
	66VO	29.58	20.88
Secretory carrier-associated membrane protein 3	*FO*	7.49	11.96
	66VO	16.01	11.32
Small heat shock protein	*FO*	420.95	871.19
	66VO	1616.51	1066.94
Splicing factor U2AF 65 kDa subunit	*FO*	70.50	116.24
	66VO	179.38	148.51
Thioredoxin	*FO*	142.42	297.60
	66VO	530.64	431.77
Thioredoxin H-type	*FO*	149.84	237.36
	66VO	452.50	356.60
Tissue factor pathway inhibitor	*FO*	10.09	11.73
	66VO	15.14	13.87
Tubulin gamma-2 chain	*FO*	24.79	48.58
	66VO	65.49	51.55
UDP-GalNAc:beta-1,3-N-acetylgalactosaminyltransferase 1	*FO*	13.08	18.79
	66VO	30.66	17.03
UDP-GlcNAc:betaGal beta-1,3-N-acetylglucosaminyltransferase 5	*FO*	17.48	26.25
	66VO	36.02	25.44
Uncharacterized mfs-type transporter c19orf28 homolog	*FO*	18.07	31.92
	66VO	59.17	43.20
Vacuolar ATP proton pump d 1	*FO*	18.23	31.23
	66VO	51.77	39.14
Vacuolar protein sorting-associated protein 26	*FO*	95.05	168.19

	66VO	275.46	209.83
Vitronectin	*FO*	244.51	378.07
	66VO	723.01	517.90

Cluster 2: Up-regulation (837 genes)

Putative Id		INF_E	INF_L
10 kDa heat shock protein, mitochondrial	*FO*	3.76	4.82
	66VO	8.21	7.03
14 kDa phosphohistidine phosphatase	*FO*	1.22	1.65
	66VO	2.42	2.61
14-3-3 protein 1	*FO*	2.83	4.36
	66VO	5.00	3.16
14-3-3 protein beta/alpha-1	*FO*	1.14	1.23
	66VO	1.79	1.43
14-3-3 protein epsilon	*FO*	1.15	1.36
	66VO	1.85	1.90
14-3-3 protein zeta	*FO*	1.66	1.68
	66VO	3.05	2.90
15 kDa selenoprotein	*FO*	1.15	1.31
	66VO	2.33	1.66
26 proteasome complex subunit DSS1	*FO*	1.30	1.58
	66VO	2.17	1.92
26S protease regulatory subunit 6B	*FO*	1.85	2.64
	66VO	4.15	3.23
26S protease regulatory subunit 7	*FO*	1.34	1.52
	66VO	2.41	1.81
26S protease regulatory subunit 8	*FO*	1.41	1.74
	66VO	2.84	2.40
26S protease regulatory subunit S10B	*FO*	1.78	2.16
	66VO	2.92	2.09
26S proteasome non-ATPase regulatory subunit 1	*FO*	1.74	2.34
	66VO	3.52	2.83
26S proteasome non-ATPase regulatory subunit 12	*FO*	1.46	1.81
	66VO	2.84	2.27
26S proteasome non-ATPase regulatory subunit 14	*FO*	1.66	2.00
	66VO	3.85	2.85
26S proteasome non-ATPase regulatory subunit 5	*FO*	1.30	1.63
	66VO	2.06	1.90
26S proteasome non-ATPase regulatory subunit 6	*FO*	1.23	1.43
	66VO	2.41	1.89
26S proteasome non-ATPase regulatory subunit 7	*FO*	1.82	2.01
	66VO	3.65	2.49
26S proteasome non-ATPase regulatory subunit 8	*FO*	1.40	1.72
	66VO	2.37	1.85
28S ribosomal protein S12, mitochondrial	*FO*	1.46	1.57
	66VO	2.93	1.88
28S ribosomal protein S17, mitochondrial	*FO*	1.29	1.56
	66VO	2.77	2.09
28S ribosomal protein S18b, mitochondrial	*FO*	1.33	1.43
	66VO	1.50	1.77
28S ribosomal protein S24-B, mitochondrial	*FO*	1.15	1.20
	66VO	2.11	1.72
28S ribosomal protein S35, mitochondrial	*FO*	1.16	1.16
	66VO	1.90	1.57
28S ribosomal protein S7, mitochondrial	*FO*	1.34	1.32
	66VO	2.42	1.88
2-amino-3-ketobutyrate coenzyme A ligase, mitochondrial	*FO*	1.23	1.40
	66VO	3.01	2.08
39S ribosomal protein 54, mitochondrial	*FO*	1.11	1.12
	66VO	1.85	1.65
39S ribosomal protein L11, mitochondrial	*FO*	1.92	2.10

	66VO	3.03	2.77
39S ribosomal protein L14, mitochondrial	*FO*	1.39	1.30
	66VO	2.75	2.16
39S ribosomal protein L16, mitochondrial	*FO*	1.53	1.55
	66VO	2.73	2.27
39S ribosomal protein L17, mitochondrial	*FO*	1.47	1.57
	66VO	2.80	2.34
39S ribosomal protein L18, mitochondrial	*FO*	1.35	1.38
	66VO	2.42	2.03
39S ribosomal protein L20, mitochondrial	*FO*	1.09	1.10
	66VO	1.89	1.49
39S ribosomal protein L28, mitochondrial	*FO*	1.47	1.44
	66VO	2.58	2.19
39S ribosomal protein L30, mitochondrial	*FO*	1.50	1.50
	66VO	2.54	2.21
39S ribosomal protein L35, mitochondrial	*FO*	1.96	2.25
	66VO	3.82	3.46
39S ribosomal protein L40, mitochondrial	*FO*	1.30	1.22
	66VO	1.92	1.73
39S ribosomal protein L43, mitochondrial	*FO*	1.45	1.59
	66VO	2.21	2.18
39S ribosomal protein L48, mitochondrial	*FO*	1.23	1.46
	66VO	2.26	2.04
39S ribosomal protein L51, mitochondrial	*FO*	1.39	1.47
	66VO	2.47	2.24
39S ribosomal protein L55, mitochondrial	*FO*	1.14	1.19
	66VO	1.77	1.68
3-beta-hydroxysteroid- -isomerase	*FO*	4.49	2.74
	66VO	14.98	14.03
3-hydroxyacyl-CoA dehydrogenase type-2	*FO*	1.54	1.67
	66VO	1.91	2.05
3-hydroxyisobutyrate dehydrogenase, mitochondrial	*FO*	1.45	1.42
	66VO	1.31	1.50
3-keto-steroid reductase	*FO*	1.78	1.58
	66VO	2.65	2.33
40S ribosomal protein S16	*FO*	4.05	7.51
	66VO	7.59	5.67
40S ribosomal protein S2-3	*FO*	4.78	8.69
	66VO	9.99	10.60
40S ribosomal protein S24-B	*FO*	4.53	9.17
	66VO	10.02	8.86
40S ribosomal protein S3	*FO*	1.70	2.32
	66VO	4.47	2.90
40S ribosomal protein S3a	*FO*	5.92	10.86
	66VO	16.14	11.67
40S ribosomal protein S4	*FO*	2.52	3.87
	66VO	4.20	2.63
40S ribosomal protein S6	*FO*	5.87	9.85
	66VO	12.18	10.26
40S ribosomal protein S9	*FO*	6.72	12.01
	66VO	10.16	8.27
4-hydroxyphenylpyruvate dioxygenase-like protein	*FO*	5.26	7.16
	66VO	19.92	13.12
60 kDa heat shock protein, mitochondrial	*FO*	6.56	8.67
	66VO	13.67	10.56
60S ribosomal protein L14	*FO*	4.91	8.86
	66VO	10.82	7.58
60S ribosomal protein L23a	*FO*	9.98	11.40
	66VO	11.88	11.99

60S ribosome subunit biogenesis protein NIP7 homolog	*FO*	1.63	2.06
	66VO	4.04	3.05
Acetyl-CoA acetyltransferase, cytosolic	*FO*	4.50	3.60
	66VO	15.42	14.71
Acetyl-coenzyme A transporter 1	*FO*	1.51	1.42
	66VO	2.09	1.94
Acidic and secreted protein	*FO*	2.52	1.97
	66VO	10.83	4.46
Acidic leucine-rich nuclear phosphoprotein 32-related protein	*FO*	3.27	5.34
	66VO	6.24	4.75
ACN9 protein homolog, mitochondrial	*FO*	1.07	1.23
	66VO	1.26	1.43
Actin, cytoplasmic 2	*FO*	1.77	1.94
	66VO	2.27	1.79
Actin-binding protein anillin	*FO*	2.09	1.67
	66VO	3.09	3.11
Actin-like protein 6A	*FO*	1.97	2.16
	66VO	5.39	3.65
Actin-related protein 2	*FO*	3.24	5.09
	66VO	8.64	5.87
Actin-related protein 2/3 complex subunit 1A	*FO*	4.90	9.73
	66VO	12.53	8.18
Actin-related protein 2/3 complex subunit 2	*FO*	1.26	1.29
	66VO	1.78	1.55
Actin-related protein 2/3 complex subunit 3	*FO*	1.18	1.32
	66VO	1.78	1.65
Actin-related protein 2/3 complex subunit 4	*FO*	1.43	1.66
	66VO	2.20	2.10
Actin-related protein 2/3 complex subunit 5	*FO*	3.86	7.19
	66VO	9.69	5.90
Actin-related protein 3	*FO*	1.59	1.83
	66VO	2.34	2.07
Activating signal cointegrator 1	*FO*	1.02	1.04
	66VO	1.30	1.25
Activator of 90 kDa heat shock protein ATPase homolog 1	*FO*	1.22	1.23
	66VO	2.78	1.68
Acyl-CoA dehydrogenase family member 9, mitochondrial	*FO*	1.48	1.74
	66VO	2.00	1.82
Acyl-CoA-binding protein	*FO*	1.36	1.30
	66VO	2.28	2.11
ADAM 23 precursor	*FO*	3.39	3.78
	66VO	4.69	4.83
Adaptin ear-binding coat-associated protein 2	*FO*	0.97	1.10
	66VO	1.50	1.39
Adenosine kinase	*FO*	2.34	2.24
	66VO	3.61	3.52
Adenylate cyclase	*FO*	3.52	4.08
	66VO	5.79	4.20
Adenylosuccinate synthetase isozyme 2	*FO*	1.21	1.25
	66VO	2.11	1.75
Adenylyl cyclase-associated protein 1	*FO*	1.21	1.57
	66VO	1.62	1.76
ADP-ribosylation factor-like protein 11	*FO*	3.84	1.91
	66VO	22.82	9.62
Alcohol dehydrogenase [NADP+]	*FO*	1.13	1.67
	66VO	2.07	1.58
Alkylated DNA repair protein alkB homolog 1	*FO*	1.64	1.79
	66VO	2.79	2.14
Allograft inflammatory factor 1	*FO*	1.43	1.32

	66VO	3.53	2.19
Alpha- and gamma-adaptin-binding protein p34	*FO*	1.03	1.15
	66VO	1.86	1.79
Aminopeptidase O	*FO*	1.57	1.70
	66VO	1.99	1.88
Ammonium transporter Rh type C 2	*FO*	3.71	5.39
	66VO	6.58	8.17
Anamorsin	*FO*	0.87	0.90
	66VO	1.75	1.22
Angiogenin	*FO*	1.82	1.37
	66VO	4.89	3.87
Annexin A3	*FO*	1.30	1.39
	66VO	2.00	1.64
AP-1 complex subunit mu-1	*FO*	1.23	1.23
	66VO	2.59	2.02
AP-2 complex subunit mu-1	*FO*	1.83	2.82
	66VO	4.40	2.65
Apolipoprotein M	*FO*	3.42	2.57
	66VO	3.78	3.00
Apolipoprotein O	*FO*	1.47	1.72
	66VO	2.00	2.44
Apoptosis inhibitor 5	*FO*	1.83	1.93
	66VO	3.14	2.76
Apoptosis-associated speck-like protein containing a CARD	*FO*	1.42	1.63
	66VO	3.16	2.52
Apoptosis-inducing factor 1, mitochondrial	*FO*	1.54	1.72
	66VO	2.06	2.14
Aprataxin	*FO*	1.32	1.50
	66VO	2.95	1.79
Aquaporin-1	*FO*	3.06	11.00
	66VO	6.84	14.01
Armadillo repeat-containing protein 1	*FO*	1.29	1.67
	66VO	1.56	2.15
Asparaginyl-tRNA synthetase, cytoplasmic	*FO*	2.05	2.22
	66VO	5.48	3.26
ATP synthase lipid-binding protein, mitochondrial	*FO*	1.46	1.44
	66VO	1.94	1.83
ATP synthase mitochondrial F1 complex assembly factor 1, mitochondrial	*FO*	1.61	1.64
	66VO	2.25	2.00
ATP synthase mitochondrial F1 complex assembly factor 2, mitochondrial	*FO*	1.19	1.35
	66VO	1.48	1.61
ATP synthase subunit alpha, mitochondrial	*FO*	1.59	1.53
	66VO	1.85	1.77
ATP synthase subunit b, mitochondrial	*FO*	1.22	1.21
	66VO	1.65	1.56
ATP synthase subunit beta, mitochondrial	*FO*	1.58	2.02
	66VO	3.48	2.74
ATP synthase subunit f, mitochondrial	*FO*	1.47	1.58
	66VO	2.16	0.83
ATP synthase subunit g, mitochondrial	*FO*	1.82	1.98
	66VO	2.99	2.67
ATP synthase subunit O, mitochondrial	*FO*	1.37	1.32
	66VO	2.11	1.97
ATPase family AAA domain-containing protein 1-B	*FO*	1.21	1.41
	66VO	2.74	2.25
ATPase family AAA domain-containing protein 2	*FO*	1.25	1.22
	66VO	1.65	1.73
ATP-binding cassette sub-family B member 8, mitochondrial	*FO*	2.02	2.55
	66VO	3.33	3.18

ATP-binding cassette sub-family E member 1	*FO*	1.54	2.03
	66VO	4.53	3.37
ATP-binding cassette sub-family F member 1	*FO*	1.78	2.25
	66VO	3.26	2.75
ATP-binding cassette sub-family F member 2	*FO*	1.53	1.80
	66VO	4.81	3.34
ATP-binding domain 1 family member C	*FO*	1.07	1.06
	66VO	1.98	1.26
ATP-dependent DNA helicase Q4	*FO*	1.11	1.35
	66VO	2.10	2.57
ATP-dependent RNA helicase DDX1	*FO*	1.37	1.37
	66VO	2.23	1.82
ATP-dependent RNA helicase DDX39	*FO*	1.52	1.69
	66VO	2.89	2.36
ATP-dependent RNA helicase DDX51	*FO*	1.48	1.61
	66VO	1.76	1.56
ATP-dependent RNA helicase SUPV3L1, mitochondrial	*FO*	1.53	1.49
	66VO	2.65	2.02
AT-rich interactive domain-containing protein 3A	*FO*	1.45	1.39
	66VO	5.63	4.72
Aurora kinase A-interacting protein	*FO*	1.42	1.32
	66VO	1.76	1.46
B- and T-lymphocyte attenuator	*FO*	3.22	2.13
	66VO	2.83	2.33
Bactericidal permeability-increasing protein	*FO*	1.65	1.42
	66VO	1.85	1.90
Barrier-to-autointegration factor	*FO*	2.45	2.34
	66VO	5.44	3.92
Basic leucine zipper transcriptional factor atf-like 3	*FO*	1.61	3.02
	66VO	5.32	3.64
Bcl-2-related proline-rich protein	*FO*	1.95	2.56
	66VO	3.79	2.91
Beta-catenin-like protein 1	*FO*	2.14	2.85
	66VO	3.36	3.24
BH3-interacting domain death agonist	*FO*	1.80	2.02
	66VO	1.81	1.31
Bifunctional purine biosynthesis protein PURH	*FO*	1.35	1.55
	66VO	2.10	2.05
Biliverdin reductase A	*FO*	2.36	2.48
	66VO	2.72	2.59
Bleomycin hydrolase	*FO*	3.02	3.49
	66VO	5.68	3.75
BolA-like protein 2	*FO*	1.31	1.46
	66VO	2.48	2.28
BolA-like protein 3	*FO*	1.24	1.20
	66VO	2.65	2.15
Borealin	*FO*	3.11	2.94
	66VO	3.09	2.71
Brix domain-containing protein 2	*FO*	2.84	3.16
	66VO	7.71	4.51
Bystin	*FO*	1.47	1.64
	66VO	2.53	2.05
C-4 methylsterol oxidase	*FO*	5.99	2.30
	66VO	19.01	14.48
Calcium/calmodulin-dependent protein kinase type II delta chain	*FO*	1.56	1.46
	66VO	1.72	1.83
Calcium-binding mitochondrial carrier protein Aralar1	*FO*	1.36	1.61
	66VO	2.09	2.05
Calreticulin	*FO*	3.23	6.36

	66VO	9.34	7.23
cAMP-regulated phosphoprotein 19	*FO*	1.11	1.24
	66VO	2.13	1.90
cAMP-responsive element modulator	*FO*	2.67	1.93
	66VO	10.70	4.30
Caprin-2	*FO*	12.19	12.19
	66VO	6.08	9.15
Carboxy-terminal kinesin 2	*FO*	2.63	2.59
	66VO	2.90	2.69
Cardiolipin synthetase	*FO*	1.09	1.14
	66VO	2.05	1.95
Caspase-6	*FO*	1.96	2.44
	66VO	2.51	2.14
Cathepsin H	*FO*	1.99	2.09
	66VO	2.65	2.68
Cathepsin L1	*FO*	1.74	1.68
	66VO	4.01	2.64
CC chemokine ligand 4	*FO*	2.07	1.92
	66VO	3.78	2.42
CCAAT/enhancer-binding protein beta	*FO*	1.76	2.13
	66VO	4.70	2.36
CCAAT/enhancer-binding protein delta	*FO*	2.40	2.15
	66VO	2.08	0.78
CCAAT/enhancer-binding protein zeta	*FO*	1.66	1.59
	66VO	2.90	2.07
CD9 antigen	*FO*	1.39	1.44
	66VO	2.32	2.09
CDGSH iron sulfur domain-containing protein 2-B	*FO*	1.30	1.50
	66VO	1.98	1.97
Cell cycle checkpoint protein RAD1	*FO*	2.81	2.43
	66VO	5.93	3.93
Cell division control protein 2 homolog	*FO*	2.53	2.52
	66VO	3.43	3.13
Cell division cycle-associated protein 7	*FO*	4.80	5.12
	66VO	3.78	6.91
Cell division protein kinase 2	*FO*	2.45	3.09
	66VO	4.74	5.05
Cell division protein kinase 4	*FO*	1.52	1.72
	66VO	2.46	1.97
Cell division protein kinase 6	*FO*	0.36	0.36
	66VO	4.29	0.24
Cell growth-regulating nucleolar protein	*FO*	1.37	1.53
	66VO	2.64	2.41
Centaurin-alpha-2	*FO*	1.22	1.17
	66VO	2.41	2.11
Centromere protein F	*FO*	1.66	1.84
	66VO	2.02	2.30
Centromere protein P	*FO*	5.17	4.87
	66VO	5.34	4.62
Centromere protein Q	*FO*	5.92	4.76
	66VO	8.42	6.00
Centromere protein S	*FO*	1.58	1.32
	66VO	3.02	2.68
Ceruloplasmin	*FO*	1.17	1.23
	66VO	3.05	2.73
C-factor	*FO*	2.41	2.41
	66VO	2.11	2.35
Cg2446-pc	*FO*	1.63	1.58
	66VO	2.00	2.10

Chloride intracellular channel 4	*FO*	1.49	2.57
	66VO	0.65	4.12
Chromatin accessibility complex protein 1	*FO*	1.62	1.42
	66VO	2.03	1.68
Chymotrypsin-C	*FO*	1.37	1.40
	66VO	2.74	2.31
Cirhin	*FO*	1.36	1.39
	66VO	2.55	1.84
Clathrin heavy chain 1	*FO*	1.14	2.04
	66VO	6.76	5.62
Cleavage and polyadenylation specificity factor subunit 1	*FO*	1.63	1.99
	66VO	2.24	2.00
Cleavage and polyadenylation specificity factor subunit 5	*FO*	1.45	1.65
	66VO	2.57	2.47
C-Myc-binding protein	*FO*	1.86	2.17
	66VO	4.35	3.16
Coatomer subunit epsilon	*FO*	1.15	1.41
	66VO	1.86	1.51
Cofilin-2	*FO*	1.22	1.42
	66VO	1.78	1.65
Coiled-coil domain-containing protein 5	*FO*	2.03	2.21
	66VO	2.99	3.01
Coiled-coil domain-containing protein 51	*FO*	1.26	1.24
	66VO	2.21	2.31
Coiled-coil domain-containing protein 56	*FO*	1.27	1.57
	66VO	2.33	2.36
Coiled-coil domain-containing protein 75	*FO*	0.98	1.16
	66VO	2.25	2.15
Coiled-coil domain-containing protein 90A, mitochondrial	*FO*	1.09	1.12
	66VO	1.81	1.91
Collagen alpha-2(VIII) chain	*FO*	5.92	3.37
	66VO	3.75	5.72
Complement C1q subcomponent subunit C	*FO*	10.99	16.36
	66VO	0.71	3.86
Complement component 1 Q subcomponent-binding protein, mitochondria	*FO*	1.83	1.86
	66VO	4.12	2.93
Complement component C8 beta chain	*FO*	2.29	4.19
	66VO	6.11	5.34
Coproporphyrinogen III oxidase, chloroplast	*FO*	3.22	5.58
	66VO	5.94	3.82
Cornifelin homolog	*FO*	1.57	1.84
	66VO	3.18	1.97
Coronin-1A	*FO*	1.30	1.46
	66VO	2.13	1.86
Coronin-1C	*FO*	1.77	1.74
	66VO	1.86	2.35
Corticotropin-lipotropin	*FO*	2.41	1.44
	66VO	2.37	1.91
C-type lectin domain family 4 member e	*FO*	1.37	1.60
	66VO	2.44	2.73
Cub and zona pellucida-like domains 1	*FO*	2.98	4.33
	66VO	9.94	14.36
Cyclin-A2	*FO*	2.94	2.80
	66VO	4.25	4.11
Cyclin-dependent kinase 2-associated protein 2	*FO*	1.09	1.37
	66VO	1.83	1.59
Cyclin-dependent kinase inhibitor 3	*FO*	1.87	1.62
	66VO	1.65	1.87
Cyclin-dependent kinases regulatory subunit 1	*FO*	2.91	2.49

	66VO	4.32	3.48
Cyclin-H	*FO*	1.19	1.19
	66VO	2.01	1.65
Cyclin-Y-like protein 1	*FO*	1.42	1.44
	66VO	2.34	2.28
Cystathionine beta-synthase	*FO*	1.82	0.97
	66VO	3.07	1.81
Cystathionine gamma-lyase	*FO*	3.12	2.85
	66VO	4.74	3.50
Cystatin-A1	*FO*	17.31	3.95
	66VO	13.68	23.41
Cysteine and histidine-rich domain-containing protein 1	*FO*	0.70	0.63
	66VO	2.58	2.66
Cysteinyl-tRNA synthetase, cytoplasmic	*FO*	4.27	4.48
	66VO	12.42	7.23
Cytochrome b-245 light chain	*FO*	1.13	1.82
	66VO	2.63	2.86
Cytochrome b5	*FO*	1.27	1.56
	66VO	3.33	2.64
Cytochrome b-c1 complex subunit Rieske, mitochondrial	*FO*	1.44	1.49
	66VO	2.05	1.82
Cytochrome c oxidase assembly protein COX15 homolog	*FO*	1.77	3.25
	66VO	5.04	2.80
Cytochrome c oxidase polypeptide VIIa-liver/heart, mitochondrial	*FO*	1.84	1.85
	66VO	3.02	2.65
Cytochrome c oxidase subunit 4 isoform 1, mitochondrial precursor	*FO*	1.12	1.14
	66VO	1.64	1.45
Cytochrome c1, heme protein, mitochondrial	*FO*	1.25	1.26
	66VO	1.77	1.58
Cytochrome c-b	*FO*	1.22	1.58
	66VO	5.97	3.00
Cytochrome P450 8B1	*FO*	3.49	2.97
	66VO	11.78	7.82
Cytohesin-1	*FO*	1.48	1.65
	66VO	3.48	2.35
Cytoplasmic light intermediate chain 2	*FO*	1.58	1.51
	66VO	3.27	2.50
Cytosol aminopeptidase	*FO*	1.41	1.47
	66VO	2.06	1.84
Cytosolic 5'-nucleotidase III	*FO*	1.43	1.56
	66VO	1.59	1.40
Cytosolic purine 5'-nucleotidase	*FO*	2.25	1.69
	66VO	4.06	4.06
D-3-phosphoglycerate dehydrogenase	*FO*	4.31	3.18
	66VO	13.93	9.06
Delta(14)-sterol reductase	*FO*	2.96	1.00
	66VO	11.23	8.98
Delta-1-pyrroline-5-carboxylate synthetase	*FO*	1.38	1.49
	66VO	2.19	2.17
Density-regulated protein	*FO*	1.69	1.95
	66VO	4.33	3.20
Denticleless homolog	*FO*	2.80	3.17
	66VO	6.54	7.39
Deoxycytidine kinase	*FO*	1.95	2.11
	66VO	3.38	2.63
Deoxyhypusine hydroxylase	*FO*	1.00	1.78
	66VO	2.95	1.71
Deoxynucleotidyltransferase terminal-interacting protein 2	*FO*	1.66	1.83
	66VO	3.40	2.48

Deoxyribonuclease-2-alpha	*FO*	2.79	4.09
	66VO	7.77	7.58
Deoxyuridine 5'-triphosphate nucleotidohydrolase, mitochondrial	*FO*	2.30	2.58
	66VO	3.83	3.47
Developmentally-regulated GTP-binding protein 1	*FO*	1.12	1.08
	66VO	1.73	1.40
Differentially regulated trout protein 1	*FO*	1.93	2.95
	66VO	19.80	8.54
Digestive cysteine proteinase 2	*FO*	4.23	6.98
	66VO	9.31	7.19
Dihydrolipoyllysine-residue acetyltransferase component of pyruvate dehy	*FO*	1.10	1.68
	66VO	4.10	2.78
Dihydroorotate dehydrogenase, mitochondrial	*FO*	2.14	2.93
	66VO	4.58	4.01
Dipeptidyl-peptidase 3	*FO*	2.91	3.90
	66VO	4.18	3.84
Diphosphomevalonate decarboxylase	*FO*	1.54	1.36
	66VO	2.96	4.20
Diphthamide biosynthesis protein 2	*FO*	1.26	1.48
	66VO	2.48	2.06
DNA (cytosine-5)-methyltrasferase 1	*FO*	2.11	2.63
	66VO	3.87	4.15
DNA mismatch repair protein Msh2 -	*FO*	1.76	1.85
	66VO	2.75	2.86
DNA polymerase delta catalytic subunit	*FO*	2.01	2.04
	66VO	4.45	4.26
DNA polymerase epsilon subunit 2	*FO*	2.55	2.33
	66VO	3.45	3.84
DNA polymerase subunit delta-2	*FO*	1.60	1.51
	66VO	2.86	2.12
DNA replication complex GINS protein PSF1	*FO*	3.43	3.82
	66VO	4.52	4.34
DNA replication complex GINS protein psf3	*FO*	1.79	2.22
	66VO	4.58	2.96
DNA replication licensing factor mcm2	*FO*	3.08	3.86
	66VO	3.98	5.09
DNA topoisomerase 2-alpha	*FO*	3.02	3.22
	66VO	4.32	3.92
DNA-(apurinic or apyrimidinic site) lyase	*FO*	1.45	1.75
	66VO	2.54	2.72
DNA-directed RNA polymerase I subunit RPA49	*FO*	1.75	2.08
	66VO	4.17	4.03
DNA-directed RNA polymerase II subunit RPB1	*FO*	0.95	1.03
	66VO	1.59	1.78
DNA-directed RNA polymerase II subunit RPB7	*FO*	1.47	2.02
	66VO	4.38	3.73
DNA-directed RNA polymerase II subunit RPB9	*FO*	1.22	1.26
	66VO	2.65	2.12
DNA-directed RNA polymerase III subunit RPC1	*FO*	1.28	1.52
	66VO	2.30	2.12
DNA-directed RNA polymerase III subunit RPC2	*FO*	1.77	2.22
	66VO	3.93	3.40
DNA-directed RNA polymerases I and III subunit RPAC1	*FO*	1.28	1.71
	66VO	3.20	2.36
DNA-directed RNA polymerases I and III subunit RPAC2	*FO*	1.45	1.82
	66VO	2.49	2.34
DNA-directed RNA polymerases I, II, and III subunit RPABC3	*FO*	2.35	2.78
	66VO	6.28	4.91
DNA-directed RNA polymerases I, II, and III subunit RPABC5	*FO*	1.23	1.32

	66VO	2.59	1.96
DnaJ homolog subfamily B member 11	*FO*	2.12	2.27
	66VO	4.10	2.97
DnaJ homolog subfamily B member 6-A	*FO*	1.08	1.11
	66VO	1.66	1.55
DnaJ homolog subfamily C member 11	*FO*	2.04	2.39
	66VO	4.31	3.52
DnaJ homolog subfamily C member 17	*FO*	1.14	1.34
	66VO	1.96	1.79
DnaJ homolog subfamily C member 2	*FO*	1.66	1.67
	66VO	2.95	2.31
Dolichol phosphate-mannose biosynthesis regulatory protein	*FO*	1.50	1.40
	66VO	1.94	1.77
Dolichyl-diphosphooligosaccharide--protein glycosyltransferase 48 kDa su	*FO*	1.77	1.98
	66VO	2.92	2.61
Dolichyl-diphosphooligosaccharide--protein glycosyltransferase 63 kDa su	*FO*	1.54	1.63
	66VO	2.46	1.90
Dolichyl-phosphate beta-glucosyltransferase	*FO*	1.71	1.86
	66VO	2.80	2.18
Double-stranded rna-dependent protein kinase	*FO*	1.31	1.59
	66VO	2.23	1.99
DPH3 homolog	*FO*	0.97	1.29
	66VO	2.21	2.06
DTW domain-containing protein 1	*FO*	1.69	2.09
	66VO	5.93	4.66
Dual specificity protein kinase CLK2	*FO*	4.65	3.54
	66VO	8.03	7.96
Dual specificity protein phosphatase 7	*FO*	1.34	1.47
	66VO	2.49	1.91
Dual specificity testis-specific protein kinase 1	*FO*	1.60	1.52
	66VO	1.46	1.80
Dynactin subunit 5	*FO*	1.38	1.54
	66VO	1.60	1.47
Dynein light chain 2, cytoplasmic	*FO*	1.73	2.90
	66VO	3.97	3.40
Ebna1 binding protein 2-like	*FO*	1.50	1.76
	66VO	3.90	2.84
EF555066-70 Ig kappa-b4 chain C region	*FO*	4.98	2.03
	66VO	14.65	6.07
EF-hand domain-containing protein D2	*FO*	1.29	1.63
	66VO	2.84	2.13
ELAV-like protein 1	*FO*	1.01	1.11
	66VO	2.02	1.66
Electron transfer flavoprotein subunit alpha, mitochondrial	*FO*	1.33	1.29
	66VO	1.77	1.56
Elongation factor 1-beta	*FO*	1.28	1.31
	66VO	1.68	1.63
Elongation factor G 1, mitochondrial	*FO*	1.94	2.47
	66VO	6.65	4.01
Elongation factor Tu GTP-binding domain-containing protein 1	*FO*	1.22	1.50
	66VO	1.63	1.80
Elongation of very long chain fatty acids protein 6	*FO*	6.24	11.86
	66VO	7.99	6.20
Endonuclease VIII-like 3	*FO*	1.91	2.10
	66VO	2.85	3.34
Endoplasmic reticulum aminopeptidase 1	*FO*	1.74	1.98
	66VO	2.42	2.37
Endoplasmic reticulum-Golgi intermediate compartment protein 1	*FO*	1.49	1.80
	66VO	3.40	2.44

ER degradation-enhancing alpha-mannosidase-like 1	*FO*	1.56	1.82
	66VO	2.28	2.07
ER lumen protein retaining receptor 2	*FO*	1.19	1.34
	66VO	2.23	1.91
ERBB receptor feedback inhibitor 1	*FO*	3.22	5.29
	66VO	14.45	5.83
Eukaryotic peptide chain release factor subunit 1	*FO*	1.29	1.27
	66VO	2.60	1.87
Eukaryotic translation initiation factor 3 subunit B	*FO*	1.43	1.66
	66VO	2.28	1.88
Eukaryotic translation initiation factor 3 subunit J	*FO*	1.00	1.06
	66VO	2.01	1.48
Eukaryotic translation initiation factor 4 gamma 1	*FO*	0.70	1.42
	66VO	2.34	0.32
Eukaryotic translation initiation factor 4 gamma 2	*FO*	1.13	1.01
	66VO	3.01	2.03
Eukaryotic translation initiation factor 4E	*FO*	1.72	2.24
	66VO	4.56	3.00
Eukaryotic translation initiation factor 4E type 2	*FO*	2.10	2.40
	66VO	3.43	2.64
Eukaryotic translation initiation factor 4E-1A	*FO*	1.20	0.99
	66VO	3.09	3.10
Exosome complex exonuclease RRP4	*FO*	1.33	1.43
	66VO	3.35	2.50
Exosome complex exonuclease RRP43	*FO*	1.14	1.13
	66VO	2.39	1.83
Exosome component 10	*FO*	1.72	1.89
	66VO	3.56	1.99
Exportin-2	*FO*	0.96	0.96
	66VO	1.82	1.73
Extensin	*FO*	5.80	5.51
	66VO	20.34	16.50
Extensin-like protein	*FO*	5.57	7.70
	66VO	11.62	7.13
F-actin-capping protein subunit alpha-1	*FO*	3.84	6.95
	66VO	8.84	6.56
F-actin-capping protein subunit beta isoforms 1 and 2	*FO*	5.70	11.54
	66VO	14.04	8.56
FAD-linked sulfhydryl oxidase ALR	*FO*	0.87	1.03
	66VO	1.75	1.22
Fanconi anemia group D2 protein	*FO*	2.64	2.61
	66VO	4.52	4.27
Farnesyl pyrophosphate synthetase	*FO*	4.57	3.82
	66VO	2.40	2.01
FAST kinase domain-containing protein 1	*FO*	1.42	1.73
	66VO	4.30	3.98
Fatty acid-binding protein, heart	*FO*	2.00	1.69
	66VO	3.75	3.67
Fc receptor-like and mucin-like 1 precursor	*FO*	1.00	1.10
	66VO	2.56	2.05
Fidgetin-like protein 1	*FO*	2.75	2.74
	66VO	3.73	4.28
FK506-binding protein 3	*FO*	1.22	1.16
	66VO	1.87	1.61
Focal adhesion kinase 1	*FO*	0.73	0.69
	66VO	0.65	0.50
Fructose-1,6-bisphosphatase 1	*FO*	2.26	2.07
	66VO	4.37	5.12
G1/S-specific cyclin-D1	*FO*	1.69	1.55

	66VO	2.25	1.96
G2/mitotic-specific cyclin-B1	*FO*	3.14	2.57
	66VO	3.07	3.08
G2/mitotic-specific cyclin-B2	*FO*	2.55	2.30
	66VO	2.31	2.53
G2/mitotic-specific cyclin-B3	*FO*	2.28	2.29
	66VO	1.39	1.81
Gamma-enolase	*FO*	3.48	5.75
	66VO	6.93	4.21
GATS 3	*FO*	3.47	2.81
	66VO	5.93	5.94
GDP-mannose 4,6 dehydratase	*FO*	2.36	2.93
	66VO	3.60	3.13
Geranylgeranyl transferase type-2 subunit beta	*FO*	1.68	2.64
	66VO	2.85	2.37
Glia maturation factor gamma	*FO*	1.34	1.41
	66VO	2.36	1.68
Glucose regulated protein 75	*FO*	1.76	1.96
	66VO	3.76	2.78
Glucose-6-phosphate 1-dehydrogenase	*FO*	2.86	2.15
	66VO	2.45	2.00
Glucosidase 2 subunit beta	*FO*	1.44	1.55
	66VO	2.19	1.87
Glutamate dehydrogenase 1, mitochondrial	*FO*	3.72	11.15
	66VO	13.41	8.59
Glutamate dehydrogenase, mitochondrial	*FO*	1.86	2.87
	66VO	4.99	5.22
Glutamate--cysteine ligase catalytic subunit	*FO*	1.19	1.54
	66VO	1.91	2.08
Glutaminyl-tRNA synthetase	*FO*	2.18	1.86
	66VO	4.22	2.98
Glutathione reductase	*FO*	1.32	1.47
	66VO	2.25	1.74
Glutathione S-transferase A	*FO*	6.40	5.85
	66VO	8.85	7.60
Glutathione S-transferase Mu 3	*FO*	1.40	1.29
	66VO	2.20	1.66
Glutathione transferase omega-1	*FO*	2.93	3.71
	66VO	5.67	3.32
Glutelin-2	*FO*	0.53	0.57
	66VO	1.60	0.67
Glycine cleavage system H protein, mitochondrial	*FO*	1.33	1.33
	66VO	2.21	2.11
Glycine N-acyltransferase	*FO*	0.91	0.91
	66VO	2.11	1.27
Glycine N-methyltransferase	*FO*	2.11	1.50
	66VO	2.59	2.88
Glycylpeptide N-tetradecanoyltransferase 1	*FO*	1.64	1.74
	66VO	2.62	2.08
GMP reductase 2	*FO*	1.25	1.41
	66VO	2.53	2.23
GPI mannosyltransferase 3	*FO*	1.39	1.84
	66VO	3.03	2.05
Grainyhead-like protein 2 homolog	*FO*	1.66	2.00
	66VO	0.75	2.75
Grainyhead-like protein 3 homolog	*FO*	0.69	0.70
	66VO	3.28	0.80
Growth factor receptor-bound protein 2	*FO*	0.71	0.56
	66VO	4.77	1.89

GrpE protein homolog 1, mitochondrial	*FO*	1.77	2.04
	66VO	3.56	2.67
Guanine nucleotide-binding protein G(I)/G(S)/G(O) subunit gamma-13	*FO*	1.29	1.12
	66VO	2.85	2.30
H/ACA ribonucleoprotein complex subunit 2-like protein	*FO*	1.91	2.36
	66VO	4.50	3.63
H/ACA ribonucleoprotein complex subunit 4	*FO*	1.96	1.99
	66VO	4.29	3.26
Haloacid dehalogenase-like hydrolase domain-containing protein 1A	*FO*	1.69	1.79
	66VO	1.85	1.75
Heat shock 70 kDa protein 4	*FO*	1.33	1.39
	66VO	2.48	1.86
Hect e3 ubiquitin ligase	*FO*	2.31	2.53
	66VO	3.53	2.43
Helical cytokine receptor crfb7	*FO*	2.09	2.01
	66VO	2.66	1.97
Heterogeneous nuclear ribonucleoprotein A1	*FO*	2.76	1.75
	66VO	3.40	4.83
HIG1 domain family member 1A	*FO*	1.35	2.31
	66VO	3.23	2.73
High affinity immunoglobulin epsilon receptor subunit gamma	*FO*	0.98	1.02
	66VO	1.97	1.57
Histone deacetylase 1	*FO*	1.20	1.44
	66VO	1.94	1.79
Histone H2A.Z	*FO*	2.41	2.86
	66VO	3.58	2.89
Histone H2AV	*FO*	1.32	1.39
	66VO	2.26	1.91
Histone H3-like centromeric protein A	*FO*	2.75	2.28
	66VO	2.76	2.68
Hiv-1 rev binding protein 2	*FO*	1.30	1.43
	66VO	1.88	1.50
Homogentisate 1,2-dioxygenase	*FO*	2.70	2.58
	66VO	5.27	2.42
Hormone-sensitive lipase	*FO*	7.71	11.68
	66VO	14.31	10.67
HpaII tiny fragments locus 9c protein	*FO*	2.17	2.43
	66VO	3.50	3.07
HRAS-like suppressor	*FO*	1.87	2.33
	66VO	0.83	1.99
HRAS-like suppressor 2	*FO*	1.32	1.19
	66VO	3.50	1.54
Hspc171 protein	*FO*	1.19	1.21
	66VO	2.55	1.91
Hydrolethalus syndrome protein 1 homolog	*FO*	1.45	1.15
	66VO	5.14	1.24
Immunoglobulin heavy chain Mem5	*FO*	12.13	2.92
	66VO	41.16	9.31
Immunoglobulin heavy chain V-III region HIL	*FO*	12.51	3.13
	66VO	41.67	9.60
Immunoglobulin kappa chain V region 3368	*FO*	4.62	1.66
	66VO	4.86	2.87
Immunoglobulin kappa chain V-VI region NQ2-6.1	*FO*	7.90	4.04
	66VO	19.35	5.37
Immunoglobulin M heavy chain	*FO*	11.68	2.40
	66VO	31.16	7.11
Import inner membrane translocase subunit TIM44, mitochondrial	*FO*	1.89	2.00
	66VO	3.25	2.62
Inorganic pyrophosphatase	*FO*	1.98	2.15

	66VO	3.78	3.02
Inositol-trisphosphate 3-kinase C	*FO*	1.26	1.32
	66VO	3.18	2.45
Integrator complex subunit 12	*FO*	1.40	1.32
	66VO	2.94	1.96
Integrin beta-1-binding protein 1	*FO*	2.50	2.53
	66VO	4.67	3.48
Integrin beta-2	*FO*	1.28	1.26
	66VO	2.20	1.81
Interferon regulatory factor 1	*FO*	2.34	2.39
	66VO	3.35	2.52
Interferon regulatory factor 8	*FO*	1.40	1.57
	66VO	2.17	1.82
Interferon-induced 35 kDa protein	*FO*	1.87	1.98
	66VO	2.30	1.76
Interferon-induced GTP-binding protein Mx2	*FO*	1.88	2.97
	66VO	2.34	2.81
Interferon-induced protein 44	*FO*	9.20	12.19
	66VO	13.35	13.78
Interferon-inducible protein gig2	*FO*	2.60	2.87
	66VO	4.31	3.68
Interleukin enhancer-binding factor 2 homolog	*FO*	3.33	4.48
	66VO	9.02	6.67
Interleukin-6	*FO*	4.85	4.96
	66VO	26.85	14.01
Interleukin-6 receptor subunit beta	*FO*	1.45	1.25
	66VO	2.38	1.96
Isoamyl acetate-hydrolyzing esterase 1 homolog	*FO*	1.32	1.44
	66VO	1.95	1.69
Isocitrate dehydrogenase [NAD] subunit beta, mitochondrial	*FO*	1.41	1.55
	66VO	2.25	1.69
Isocitrate dehydrogenase [NADP] cytoplasmic	*FO*	3.60	3.38
	66VO	6.26	4.96
Isopentenyl-diphosphate Delta-isomerase 1	*FO*	1.22	1.08
	66VO	1.61	1.83
Kallikrein-8	*FO*	4.68	3.28
	66VO	4.98	6.00
Keratin, type I cytoskeletal 13	*FO*	0.37	0.61
	66VO	1.81	2.56
KH domain-containing, RNA-binding, signal transduction-associated prote	*FO*	0.93	1.07
	66VO	1.66	1.49
Kh-type splicing regulatory protein	*FO*	1.91	1.85
	66VO	4.10	3.58
Kinesin family member 15	*FO*	4.13	3.85
	66VO	3.62	3.47
Kinesin family member 18a	*FO*	2.66	2.38
	66VO	3.36	2.85
Kinetochore-associated protein 1	*FO*	3.21	3.01
	66VO	6.40	5.43
Kinetochore-associated protein DSN1 homolog	*FO*	1.84	1.59
	66VO	4.12	3.24
Kinetochore-associated protein NSL1 homolog	*FO*	2.16	1.71
	66VO	4.29	3.60
Kynurenine 3-monooxygenase	*FO*	2.14	1.94
	66VO	3.74	2.86
LAS1-like protein	*FO*	1.10	1.18
	66VO	2.13	1.80
Lathosterol oxidase	*FO*	1.83	0.84
	66VO	3.25	2.65

Leucine-rich PPR motif-containing protein, mitochondrial	*FO*	2.28	2.69
	66VO	4.74	4.04
Leucine-rich repeat-containing protein 20	*FO*	1.03	1.05
	66VO	2.05	2.23
Leucine-rich repeats and immunoglobulin-like domains protein 1	*FO*	1.64	1.92
	66VO	5.75	5.62
Low-density lipoprotein receptor-related protein 2	*FO*	1.08	1.27
	66VO	1.81	1.47
Lupus La protein homolog B	*FO*	1.54	2.01
	66VO	3.62	2.82
Lymphocyte cytosolic protein 2	*FO*	1.35	1.39
	66VO	1.42	1.86
Lymphokine-activated killer T-cell-originated protein kinase homolog	*FO*	2.50	2.40
	66VO	4.41	3.16
LYR motif-containing protein 4	*FO*	1.78	2.06
	66VO	4.55	3.01
LYR motif-containing protein 7	*FO*	1.02	1.23
	66VO	1.81	1.67
Lysine-specific histone demethylase 1	*FO*	1.06	1.29
	66VO	1.49	1.63
Lysosome-associated membrane glycoprotein 3	*FO*	1.23	2.18
	66VO	1.67	2.50
Lysozyme g	*FO*	2.03	2.47
	66VO	3.56	0.83
Lysyl-tRNA synthetase	*FO*	1.97	2.28
	66VO	3.58	2.95
Macrophage migration inhibitory factor	*FO*	1.30	1.24
	66VO	1.80	1.89
MAD2L1-binding protein	*FO*	4.11	4.09
	66VO	7.21	5.68
Mannosyl-oligosaccharide glucosidase	*FO*	1.28	1.46
	66VO	2.49	2.06
Matrix metallopeptidase 13	*FO*	2.20	2.47
	66VO	5.83	4.43
Mediator of RNA polymerase II transcription subunit 10	*FO*	1.07	1.04
	66VO	1.60	1.29
Mediator of RNA polymerase II transcription subunit 18	*FO*	1.03	1.08
	66VO	1.81	1.67
Mediator of RNA polymerase II transcription subunit 20	*FO*	1.33	1.46
	66VO	2.66	2.24
Melanoma-associated antigen G1	*FO*	1.22	1.27
	66VO	2.77	2.14
Methylmalonic aciduria and homocystinuria type C homolog	*FO*	1.34	1.51
	66VO	2.68	2.70
Methyltransferase-like protein 1	*FO*	1.97	2.07
	66VO	4.47	2.87
Methyltransferase-like protein 2A	*FO*	1.54	1.59
	66VO	2.40	2.07
Microsomal glutathione S-transferase 3	*FO*	3.10	3.08
	66VO	4.00	4.30
Mif4g domain containing protein	*FO*	3.25	5.69
	66VO	6.02	4.18
Mitochondrial 28S ribosomal protein S22	*FO*	1.30	1.31
	66VO	2.07	1.69
Mitochondrial 28S ribosomal protein S25	*FO*	1.17	1.22
	66VO	1.90	1.68
Mitochondrial 28S ribosomal protein S28	*FO*	1.55	1.71
	66VO	2.67	2.00
Mitochondrial 39S ribosomal protein L23	*FO*	2.14	1.58

	66VO	3.02	2.26
Mitochondrial 39S ribosomal protein L3	*FO*	1.34	1.41
	66VO	1.94	1.81
Mitochondrial 39S ribosomal protein L49	*FO*	1.35	1.32
	66VO	2.85	2.33
Mitochondrial carrier homolog 2	*FO*	2.11	1.96
	66VO	4.18	3.11
Mitochondrial chaperone BCS1	*FO*	1.83	2.01
	66VO	3.50	3.03
Mitochondrial glutamate carrier 1	*FO*	1.60	1.85
	66VO	3.43	2.59
Mitochondrial import inner membrane translocase subunit Tim17-B	*FO*	1.14	1.23
	66VO	1.95	1.37
Mitochondrial import inner membrane translocase subunit Tim22	*FO*	1.73	1.84
	66VO	4.45	3.50
Mitochondrial import receptor subunit TOM40 homolog	*FO*	2.80	3.10
	66VO	8.98	6.24
Mitochondrial intermembrane space import and assembly protein 40	*FO*	1.86	2.16
	66VO	5.14	3.65
Mitochondrial ornithine transporter 1	*FO*	4.22	5.75
	66VO	6.15	6.28
Mitochondrial ribosomal protein S23	*FO*	1.26	1.36
	66VO	2.38	1.99
Mitochondrial-processing peptidase subunit alpha, mitochondrial	*FO*	1.29	1.29
	66VO	2.31	1.90
Mitoferrin-1	*FO*	1.58	1.81
	66VO	2.66	1.84
Mitotic checkpoint protein BUB3	*FO*	1.53	1.71
	66VO	3.13	2.77
Mitotic spindle assembly checkpoint protein MAD2A	*FO*	3.73	3.70
	66VO	5.75	4.16
MKI67 FHA domain-interacting nucleolar phosphoprotein-like	*FO*	1.56	1.53
	66VO	2.92	2.44
mRNA export factor	*FO*	1.05	1.07
	66VO	2.24	1.76
mRNA turnover protein 4 homolog	*FO*	1.18	1.32
	66VO	2.79	1.89
mRNA-capping enzyme	*FO*	2.76	4.03
	66VO	4.75	3.16
Mucin-3A	*FO*	1.32	1.32
	66VO	2.61	2.70
Multifunctional protein ADE2	*FO*	1.85	1.73
	66VO	3.17	3.37
MYC-induced nuclear antigen	*FO*	1.24	1.26
	66VO	1.64	1.52
Myosin-Ie	*FO*	1.23	1.47
	66VO	2.07	1.82
N-acetyltransferase 10	*FO*	1.93	2.47
	66VO	4.16	3.13
N-acetyltransferase NAT13	*FO*	1.55	1.52
	66VO	3.46	2.46
NADH dehydrogenase [ubiquinone] 1 alpha subcomplex subunit 10, mitoc	*FO*	1.34	1.30
	66VO	1.69	1.64
NADH dehydrogenase [ubiquinone] 1 alpha subcomplex subunit 13	*FO*	1.39	1.48
	66VO	2.12	2.04
NADH dehydrogenase [ubiquinone] 1 alpha subcomplex subunit 6	*FO*	1.35	1.20
	66VO	1.87	1.91
NADH dehydrogenase [ubiquinone] 1 alpha subcomplex subunit 8	*FO*	1.06	0.97
	66VO	1.66	1.45

NADH dehydrogenase [ubiquinone] 1 alpha subcomplex subunit 9, mitoch	*FO*	1.32	1.26
	66VO	1.63	1.63
NADH dehydrogenase [ubiquinone] 1 beta subcomplex subunit 5, mitocho	*FO*	1.34	1.44
	66VO	2.12	1.94
NADH dehydrogenase [ubiquinone] flavoprotein 2, mitochondrial	*FO*	1.41	1.54
	66VO	1.94	2.01
NADH dehydrogenase [ubiquinone] iron-sulfur protein 2, mitochondrial	*FO*	1.37	1.39
	66VO	1.66	1.52
Nardilysin	*FO*	1.23	1.37
	66VO	1.87	1.75
Nascent polypeptide-associated complex subunit alpha	*FO*	6.61	11.62
	66VO	12.33	10.63
Natural killer-enhancing factor A	*FO*	2.71	3.03
	66VO	2.89	2.59
NEDD8	*FO*	1.39	1.59
	66VO	2.78	2.27
Neighbor of COX4	*FO*	1.77	1.95
	66VO	4.59	3.19
Neutral alpha-glucosidase AB	*FO*	1.66	1.74
	66VO	1.59	1.52
NHP2-like protein 1	*FO*	2.15	2.72
	66VO	5.94	4.25
Nicotinamide phosphoribosyltransferase	*FO*	1.52	1.44
	66VO	3.26	2.50
Nicotinamide riboside kinase 2	*FO*	2.30	2.67
	66VO	4.13	2.74
Nicotinic acid receptor 1	*FO*	2.24	1.77
	66VO	1.79	2.34
NIF3-like protein 1	*FO*	1.30	1.39
	66VO	1.79	1.71
N-myc-interactor	*FO*	1.12	1.37
	66VO	2.11	1.85
Nocturnin	*FO*	2.09	2.52
	66VO	4.79	2.90
Nogo-B receptor	*FO*	2.08	1.65
	66VO	2.81	2.80
Nuclear cap-binding protein subunit 2	*FO*	1.18	1.27
	66VO	2.18	1.93
Nuclear pore complex protein Nup155	*FO*	1.29	1.52
	66VO	2.50	2.35
Nuclear pore membrane glycoprotein 210	*FO*	2.51	3.17
	66VO	3.57	3.11
Nuclear prelamin A recognition factor	*FO*	1.15	1.18
	66VO	2.06	1.97
Nuclear protein Hcc-1	*FO*	1.86	2.43
	66VO	3.88	3.31
Nuclear receptor 2C2-associated protein	*FO*	1.40	1.73
	66VO	3.62	2.89
Nuclear transport factor 2	*FO*	1.52	1.70
	66VO	3.09	2.43
Nucleolar and spindle-associated protein 1	*FO*	2.63	2.73
	66VO	2.85	3.02
Nucleolar GTP-binding protein 2	*FO*	2.18	2.08
	66VO	2.46	1.77
Nucleolar phosphoprotein p130	*FO*	1.23	1.31
	66VO	2.53	1.99
Nucleolar protein 5	*FO*	2.15	2.42
	66VO	5.82	3.92
Nucleolar protein 5A	*FO*	1.23	1.25

	66VO	2.26	1.72
NudC domain-containing protein 2	*FO*	1.16	1.14
	66VO	2.55	1.93
Obg-like ATPase 1	*FO*	1.56	1.83
	66VO	2.18	1.95
OCIA domain-containing protein 1	*FO*	1.23	1.18
	66VO	1.77	1.61
Oligoribonuclease, mitochondrial	*FO*	1.42	1.56
	66VO	2.06	1.95
Opioid growth factor receptor-like protein 1	*FO*	2.79	4.13
	66VO	3.48	2.76
Optic atrophy 3 protein	*FO*	1.51	1.82
	66VO	2.44	2.06
Origin recognition complex subunit 5	*FO*	2.57	2.68
	66VO	4.47	5.04
Ornithine aminotransferase, mitochondrial	*FO*	1.60	1.30
	66VO	3.21	1.72
Ornithine carbamoyltransferase, mitochondrial	*FO*	3.68	3.70
	66VO	10.54	6.49
Ornithine decarboxylase	*FO*	5.61	7.12
	66VO	31.99	16.19
OTU domain-containing protein 6B	*FO*	1.42	1.61
	66VO	2.01	2.07
OX-2 membrane glycoprotein precursor	*FO*	3.81	3.17
	66VO	3.82	4.07
p21-activated protein kinase-interacting protein 1-like	*FO*	1.92	2.07
	66VO	4.84	3.44
PAB-dependent poly(A)-specific ribonuclease subunit 2	*FO*	1.23	1.26
	66VO	1.33	1.41
Palmitoyl-protein thioesterase 1	*FO*	5.38	9.64
	66VO	14.37	9.98
Papaya proteinase 4	*FO*	6.93	8.36
	66VO	11.69	8.84
Para-hydroxybenzoate--polyprenyltransferase, mitochondrial	*FO*	1.39	1.58
	66VO	2.31	2.37
PC4 and SFRS1-interacting protein	*FO*	2.10	2.31
	66VO	3.26	3.39
PCI domain-containing protein 2	*FO*	1.18	1.18
	66VO	1.71	1.56
Pentatricopeptide repeat domain 2	*FO*	1.08	1.11
	66VO	1.83	1.60
Pentatricopeptide repeat-containing protein 3, mitochondrial	*FO*	1.44	1.58
	66VO	2.44	2.26
Peptidyl-prolyl cis-trans isomerase	*FO*	1.98	2.34
	66VO	3.30	2.40
Peptidyl-prolyl cis-trans isomerase A	*FO*	1.42	1.69
	66VO	2.32	2.10
Peptidyl-prolyl cis-trans isomerase E	*FO*	1.29	1.33
	66VO	2.14	1.79
Peptidyl-prolyl cis-trans isomerase H	*FO*	1.49	1.64
	66VO	3.13	2.15
Peptidyl-prolyl cis-trans isomerase-like 1	*FO*	2.54	2.83
	66VO	6.72	4.86
Peptidyl-prolyl cis-trans isomerase-like 3	*FO*	1.44	1.56
	66VO	3.06	2.51
Perforin-1	*FO*	5.84	2.97
	66VO	4.50	9.53
Peroxiredoxin-1	*FO*	3.79	4.72
	66VO	3.16	2.76

Peroxisome proliferator-activated receptor gamma coactivator-related pro	*FO*	5.33	6.13
	66VO	21.30	13.72
PHD finger-like domain-containing protein 5A	*FO*	1.98	2.18
	66VO	4.12	3.42
Phosphatidylcholine transfer protein	*FO*	2.31	3.54
	66VO	3.66	4.45
Phosphatidylcholine-sterol acyltransferase	*FO*	1.27	2.29
	66VO	0.54	2.18
Phosphatidylethanolamine-binding protein 1	*FO*	1.45	1.45
	66VO	2.16	1.94
Phosphatidylinositide phosphatase SAC1-B	*FO*	1.23	1.45
	66VO	2.08	1.87
Phosphatidylinositol N-acetylglucosaminyltransferase subunit Y	*FO*	1.40	1.30
	66VO	1.78	1.73
Phosphatidylinositol transfer protein beta isoform	*FO*	1.88	1.72
	66VO	2.22	2.24
Phosphoacetylglucosamine mutase	*FO*	1.39	1.68
	66VO	1.90	1.84
Phosphoglycerate mutase 1	*FO*	1.34	1.21
	66VO	1.73	1.28
Phospholipase D4	*FO*	1.34	1.36
	66VO	1.90	2.27
Phospholipid hydroperoxide glutathione peroxidase, mitochondrial	*FO*	2.04	1.81
	66VO	2.71	2.03
PIH1 domain-containing protein 1	*FO*	1.24	1.20
	66VO	1.72	1.44
Piwi-like protein 2	*FO*	6.49	1.84
	66VO	7.08	5.03
Plasminogen activator inhibitor 1 RNA-binding protein	*FO*	1.24	1.26
	66VO	1.86	1.66
PMS1 protein homolog 2	*FO*	0.66	0.48
	66VO	3.83	0.43
Poly(A) RNA polymerase, mitochondrial	*FO*	1.89	2.12
	66VO	3.04	2.78
Poly(ADP-ribose) glycohydrolase	*FO*	2.56	2.53
	66VO	3.44	2.67
Poly(rC)-binding protein 2	*FO*	1.75	1.91
	66VO	3.00	2.55
Poly(U)-binding-splicing factor PUF60	*FO*	1.47	0.97
	66VO	2.23	2.28
Polymeric immunoglobulin receptor	*FO*	2.38	2.22
	66VO	4.22	4.30
PRA1 family protein 3	*FO*	1.52	1.85
	66VO	3.73	2.47
Prefoldin subunit 2	*FO*	1.06	1.10
	66VO	1.65	1.53
Prefoldin subunit 4	*FO*	1.24	1.47
	66VO	2.03	2.05
Prefoldin subunit 6	*FO*	1.29	1.40
	66VO	2.84	2.23
Pre-mRNA-processing factor 40 homolog A	*FO*	1.48	1.87
	66VO	2.07	1.94
Pre-rRNA-processing protein TSR1 homolog	*FO*	1.90	2.24
	66VO	3.34	2.73
Presequence protease, mitochondrial	*FO*	1.89	1.79
	66VO	6.07	3.75
Probable asparaginyl-tRNA synthetase, mitochondrial	*FO*	1.39	1.37
	66VO	1.92	1.83
Probable ATP-dependent RNA helicase DDX5	*FO*	2.08	2.63

	66VO	3.67	3.08
Probable clathrin heavy chain 1	*FO*	1.84	2.82
	66VO	3.50	1.96
Probable dimethyladenosine transferase	*FO*	1.07	1.32
	66VO	3.03	2.03
Probable E3 ubiquitin-protein ligase HERC6	*FO*	1.51	1.57
	66VO	1.88	1.56
Probable glutamyl-tRNA synthetase, mitochondrial	*FO*	1.35	1.56
	66VO	2.26	1.68
Probable G-protein coupled receptor 39	*FO*	0.71	0.63
	66VO	2.35	0.42
Probable nucleolar protein NOP5-2	*FO*	1.39	2.37
	66VO	2.84	2.12
Probable O-sialoglycoprotein endopeptidase	*FO*	1.19	1.17
	66VO	2.19	1.70
Probable signal peptidase complex subunit 2	*FO*	1.71	1.76
	66VO	2.52	1.80
Probable thiopurine S-methyltransferase	*FO*	2.21	2.61
	66VO	2.31	2.41
Probable tRNA pseudouridine synthase 1	*FO*	1.05	1.28
	66VO	1.70	1.60
Profilin-2	*FO*	1.39	1.48
	66VO	2.55	1.91
Programmed cell death protein 5	*FO*	1.13	1.22
	66VO	2.07	1.82
Prohibitin-2	*FO*	1.61	1.69
	66VO	2.53	2.01
Proliferating cell nuclear antigen	*FO*	1.86	2.02
	66VO	3.35	3.14
Proliferation-associated protein 2G4	*FO*	1.98	2.20
	66VO	4.54	3.60
Proline-serine-threonine phosphatase-interacting protein 1	*FO*	1.58	1.49
	66VO	2.16	2.12
Prolyl 3-hydroxylase 2 precursor	*FO*	1.53	1.85
	66VO	1.92	2.13
Prostaglandin E synthase 3	*FO*	1.80	2.04
	66VO	5.28	3.75
Proteasome activator complex subunit 1	*FO*	1.72	1.77
	66VO	2.64	2.32
Proteasome activator complex subunit 2	*FO*	2.21	2.44
	66VO	3.19	2.82
Proteasome activator complex subunit 3	*FO*	1.27	1.39
	66VO	2.88	2.10
Proteasome assembly chaperone 3	*FO*	1.55	1.79
	66VO	2.67	2.23
Proteasome maturation protein	*FO*	1.55	1.76
	66VO	3.09	2.19
Proteasome subunit alpha type-1	*FO*	2.12	2.57
	66VO	4.26	3.17
Proteasome subunit alpha type-2	*FO*	1.94	2.44
	66VO	4.25	3.20
Proteasome subunit alpha type-3	*FO*	2.11	2.13
	66VO	3.44	2.15
Proteasome subunit alpha type-4	*FO*	1.76	1.90
	66VO	2.84	2.32
Proteasome subunit alpha type-6	*FO*	1.53	1.65
	66VO	2.63	2.12
Proteasome subunit alpha type-7	*FO*	1.68	1.96
	66VO	3.02	2.33

Proteasome subunit beta type-1-B	*FO*	1.40	1.63
	66VO	2.37	1.90
Proteasome subunit beta type-2	*FO*	1.80	1.97
	66VO	3.17	2.31
Proteasome subunit beta type-3	*FO*	2.17	3.02
	66VO	5.18	3.87
Proteasome subunit beta type-5	*FO*	1.70	2.32
	66VO	2.68	1.76
Proteasome subunit beta type-7	*FO*	2.36	2.48
	66VO	4.81	3.68
Proteasome subunit beta type-9	*FO*	1.70	1.71
	66VO	2.33	2.32
Protein arginine N-methyltransferase 1	*FO*	3.05	3.47
	66VO	7.75	5.18
Protein arginine N-methyltransferase 3	*FO*	1.81	2.11
	66VO	2.86	2.51
Protein arginine N-methyltransferase 6	*FO*	1.48	1.74
	66VO	2.77	2.75
Protein BAT5	*FO*	1.19	1.50
	66VO	1.74	1.84
Protein BCCIP homolog	*FO*	1.91	2.02
	66VO	3.37	2.92
Protein BTG3	*FO*	1.71	1.87
	66VO	2.45	1.55
Protein BUD31 homolog	*FO*	1.02	1.18
	66VO	1.97	1.59
Protein canopy homolog 2	*FO*	1.55	2.10
	66VO	3.28	2.36
Protein chibby homolog 1	*FO*	1.23	1.32
	66VO	2.13	1.71
Protein CWC15 homolog	*FO*	1.56	1.32
	66VO	3.66	4.15
Protein deltex-1	*FO*	1.47	1.33
	66VO	1.79	1.82
Protein disulfide-isomerase A4	*FO*	1.17	1.07
	66VO	2.29	1.76
Protein disulfide-isomerase A6	*FO*	1.72	1.60
	66VO	3.29	2.53
Protein FAM139A	*FO*	5.72	5.32
	66VO	5.44	6.73
Protein FAM36A	*FO*	1.58	1.79
	66VO	1.99	1.92
Protein FAM54A	*FO*	1.88	1.87
	66VO	2.34	2.19
Protein FAM60A	*FO*	1.33	1.33
	66VO	1.85	1.73
Protein FAM82B	*FO*	1.72	1.44
	66VO	1.92	1.91
Protein LSM12 homolog A	*FO*	1.05	1.05
	66VO	1.92	1.60
Protein LYRIC	*FO*	1.38	1.67
	66VO	2.39	2.06
Protein mago nashi homolog	*FO*	1.23	1.44
	66VO	2.39	1.99
Protein NEDD1	*FO*	2.63	3.69
	66VO	13.31	3.14
Protein RCC2 homolog	*FO*	1.60	1.87
	66VO	3.97	2.97
Protein RER1	*FO*	0.95	1.20

	66VO	1.72	1.49
Protein S100-A1	*FO*	5.34	4.04
	66VO	9.95	18.27
Protein SEC13 homolog	*FO*	1.12	1.55
	66VO	2.37	1.88
Protein transport protein Sec23A	*FO*	2.10	2.13
	66VO	3.94	3.23
Protoporphyrinogen oxidase	*FO*	1.52	1.59
	66VO	2.69	2.42
Pterin-4-alpha-carbinolamine dehydratase	*FO*	1.05	1.03
	66VO	1.54	1.50
Pumilio domain-containing protein C14orf21 homolog	*FO*	1.18	1.21
	66VO	1.83	1.55
Putative ATP-dependent Clp protease proteolytic subunit, mitochondrial	*FO*	1.43	1.42
	66VO	2.29	1.87
Putative hexokinase HKDC1	*FO*	2.28	2.69
	66VO	1.87	2.32
Putative oxidoreductase yjhC	*FO*	1.53	1.73
	66VO	1.65	1.78
Putative phosphoethanolamine N-methyltransferase 3	*FO*	3.01	2.84
	66VO	11.38	5.46
Putative pre-mRNA-splicing factor ATP-dependent RNA helicase DHX15	*FO*	1.52	1.65
	66VO	2.19	1.85
Putative RNA exonuclease NEF-sp	*FO*	2.92	8.09
	66VO	5.62	10.42
Putative RNA methyltransferase NOL1	*FO*	2.17	2.23
	66VO	4.75	3.42
Putative thiosulfate sulfurtransferase KAT	*FO*	2.66	3.52
	66VO	5.28	4.44
Pyruvate dehydrogenase [lipoamide]]-phosphatase 2, mitochondrial	*FO*	1.35	1.47
	66VO	2.68	2.14
Ran GTPase-activating protein 1	*FO*	1.23	1.30
	66VO	2.67	1.87
Ras GTPase-activating-like protein IQGAP2	*FO*	2.53	2.84
	66VO	2.72	2.93
Ras-related protein Rab-8A	*FO*	1.21	1.19
	66VO	2.37	2.01
Ras-related protein Rap-1	*FO*	1.41	1.40
	66VO	2.15	2.02
Ras-related protein Rap-1b	*FO*	1.38	2.14
	66VO	1.69	1.46
Receptor-binding cancer antigen expressed on SiSo cells	*FO*	1.20	1.33
	66VO	1.96	1.65
Recombination activating gene 1 activating protein 1	*FO*	1.28	1.09
	66VO	2.22	1.66
Regulator of telomere elongation helicase 1	*FO*	1.30	1.20
	66VO	1.61	1.77
Replication factor C subunit 3	*FO*	1.83	1.72
	66VO	2.74	2.58
Replication factor C subunit 4	*FO*	1.86	2.02
	66VO	3.33	3.15
Replication protein A 32 kDa subunit	*FO*	1.95	2.18
	66VO	3.48	3.78
Reticulon-4-interacting protein 1 homolog, mitochondrial	*FO*	1.65	1.74
	66VO	2.67	2.59
Retinol dehydrogenase 12	*FO*	1.66	1.70
	66VO	4.11	2.86
Rho GDP-dissociation inhibitor 1	*FO*	1.22	1.37
	66VO	1.73	1.68

Rho-related GTP-binding protein RhoF	*FO*	1.29	1.45
	66VO	2.22	1.81
Ribonuclease P protein subunit p29	*FO*	1.18	1.16
	66VO	1.78	1.48
Ribonucleoside-diphosphate reductase large subunit	*FO*	3.05	3.19
	66VO	6.24	5.21
Ribonucleoside-diphosphate reductase subunit M2	*FO*	2.79	2.50
	66VO	4.92	3.85
Ribosomal protein L22-like 1	*FO*	1.21	1.52
	66VO	2.24	2.24
Ribosomal protein L7-like 1	*FO*	1.23	1.42
	66VO	2.60	2.17
RING finger and WD repeat domain-containing protein 3	*FO*	1.00	1.12
	66VO	1.76	1.67
RING-box protein 2	*FO*	1.02	1.06
	66VO	2.11	1.67
RNA methyltransferase-like protein 1A	*FO*	1.65	1.26
	66VO	4.92	2.54
RNA-binding protein 28	*FO*	1.58	1.84
	66VO	3.35	2.29
RNA-binding protein 4	*FO*	1.08	1.33
	66VO	1.48	1.86
RNA-binding protein 8A	*FO*	1.05	1.20
	66VO	1.69	1.51
rRNA 2'-O-methyltransferase fibrillarin	*FO*	1.82	2.36
	66VO	4.18	3.07
rRNA-processing protein FCF1 homolog	*FO*	1.26	1.36
	66VO	2.73	2.08
RuvB-like 1	*FO*	1.96	2.15
	66VO	4.21	3.50
RWD domain-containing protein 1	*FO*	1.28	1.36
	66VO	1.75	1.62
Sarcoma antigen NY-SAR-48	*FO*	1.29	1.33
	66VO	2.44	2.06
Scavenger mRNA-decapping enzyme DcpS	*FO*	2.62	3.22
	66VO	4.01	3.87
Securin	*FO*	2.27	2.15
	66VO	2.50	2.77
Selenide, water dikinase	*FO*	2.36	2.19
	66VO	2.67	2.15
Selenide, water dikinase 1	*FO*	2.90	3.30
	66VO	3.45	3.27
Selenide, water dikinase 2	*FO*	1.85	2.12
	66VO	3.37	2.79
Selenoprotein H	*FO*	1.89	2.24
	66VO	3.18	3.31
Septin-2	*FO*	1.01	1.33
	66VO	1.62	1.62
Serine protease HTRA2, mitochondrial	*FO*	1.36	1.11
	66VO	2.81	1.58
Serine/threonine-protein kinase 19	*FO*	1.37	1.52
	66VO	3.58	3.21
Serine/threonine-protein kinase Eg2-like	*FO*	4.11	3.84
	66VO	4.06	3.79
Serine/threonine-protein kinase Nek2	*FO*	2.14	1.80
	66VO	2.65	2.14
Serine-threonine kinase receptor-associated protein	*FO*	1.85	1.52
	66VO	2.37	2.22
Serpin B6	*FO*	0.86	1.09

	66VO	2.15	1.59
Serum paraoxonase/arylesterase 2	*FO*	2.90	2.09
	66VO	4.07	4.70
Serum response factor-binding protein 1	*FO*	1.41	1.55
	66VO	3.16	2.38
Seryl-tRNA synthetase	*FO*	2.39	2.49
	66VO	3.32	2.71
Shugoshin-like 1	*FO*	2.84	2.83
	66VO	2.95	2.94
Sialidase-3	*FO*	7.27	5.69
	66VO	22.17	21.01
Signal recognition particle 14 kDa protein	*FO*	1.11	1.22
	66VO	2.15	1.50
Signal recognition particle 9 kDa protein	*FO*	1.77	1.77
	66VO	2.13	1.68
Signal transducer and activator of transcription 1	*FO*	1.53	1.60
	66VO	2.50	2.06
Small inducible cytokine scya104	*FO*	5.45	12.69
	66VO	8.86	7.44
Small nuclear ribonucleoprotein E	*FO*	1.66	2.00
	66VO	2.78	2.12
Small nuclear ribonucleoprotein F	*FO*	2.51	3.18
	66VO	6.83	5.46
Small nuclear ribonucleoprotein G	*FO*	1.95	2.44
	66VO	3.98	3.32
Small nuclear ribonucleoprotein Sm D2	*FO*	1.69	2.42
	66VO	4.38	3.56
Small nuclear ribonucleoprotein Sm D3	*FO*	1.19	1.41
	66VO	2.31	1.81
Sodium- and chloride-dependent gaba transporter 2	*FO*	2.07	2.25
	66VO	2.41	2.33
Sodium channel subunit beta-1	*FO*	2.05	2.02
	66VO	4.04	3.80
Solute carrier family 22 member 5	*FO*	1.12	1.00
	66VO	2.38	1.86
Spectrin beta chain, brain 2	*FO*	3.07	4.90
	66VO	5.12	3.92
S-phase kinase-associated protein 2	*FO*	2.20	2.21
	66VO	5.53	4.28
Splicing factor U2AF 35 kDa subunit	*FO*	1.44	1.45
	66VO	2.25	1.93
Squalene monooxygenase	*FO*	2.00	0.75
	66VO	6.83	6.29
Start domain containing 10	*FO*	3.26	3.26
	66VO	10.60	5.49
Stress-induced-phosphoprotein 1	*FO*	1.22	1.27
	66VO	2.03	1.79
Succinate dehydrogenase [ubiquinone] cytochrome b small subunit, mitoc	*FO*	1.61	1.80
	66VO	1.77	1.59
Succinate dehydrogenase [ubiquinone] flavoprotein subunit, mitochondria	*FO*	1.83	1.80
	66VO	2.02	1.93
Superoxide dismutase [Cu-Zn]	*FO*	3.93	8.25
	66VO	10.70	5.69
Supervillin	*FO*	1.43	1.67
	66VO	1.96	1.69
SWI/SNF-related matrix-associated actin-dependent regulator of chromat	*FO*	1.17	1.29
	66VO	1.84	1.65
Targeting protein for Xklp2	*FO*	2.40	2.49
	66VO	3.12	3.04

Tata box binding protein -associated rna polymerase b	*FO*	0.82	1.08
	66VO	4.35	0.77
Tax1-binding protein 3	*FO*	1.43	1.85
	66VO	2.24	1.94
T-cell receptor beta chain T17T-22	*FO*	3.53	2.14
	66VO	6.39	2.29
T-complex protein 1 subunit delta	*FO*	1.69	1.86
	66VO	2.68	2.27
T-complex protein 1 subunit epsilon	*FO*	1.19	1.34
	66VO	2.06	1.75
T-complex protein 1 subunit eta	*FO*	1.50	1.68
	66VO	2.27	2.11
Testin	*FO*	1.78	1.94
	66VO	2.14	2.23
Thioredoxin	*FO*	1.96	2.50
	66VO	3.22	2.65
Thioredoxin-like protein 1	*FO*	1.51	1.84
	66VO	3.83	2.52
Thioredoxin-like protein 4A	*FO*	1.41	1.63
	66VO	2.06	1.43
THO complex subunit 7 homolog	*FO*	1.22	1.27
	66VO	2.04	1.98
Threonyl-tRNA synthetase, cytoplasmic	*FO*	2.97	3.72
	66VO	8.60	5.96
THUMP domain-containing protein 1	*FO*	2.23	2.35
	66VO	5.33	4.03
Thymidine kinase 2, mitochondrial	*FO*	1.16	1.25
	66VO	3.44	2.25
Thymidylate kinase	*FO*	1.22	1.27
	66VO	2.37	2.01
Thymidylate synthase	*FO*	2.72	3.23
	66VO	3.42	3.77
Toll-like receptor 9 isoform A	*FO*	1.46	2.35
	66VO	1.40	1.61
Transcription elongation factor A protein 1	*FO*	1.33	1.68
	66VO	2.70	2.27
Transcription factor BTF3 homolog 4	*FO*	1.25	1.31
	66VO	3.05	2.08
Transcription factor Dp-1	*FO*	1.38	1.38
	66VO	1.45	1.80
Transcription factor PU.1	*FO*	1.14	1.16
	66VO	2.02	1.55
Transcription factor SOX-6	*FO*	0.66	1.07
	66VO	4.61	0.42
Transcription initiation factor TFIID subunit 13	*FO*	1.12	1.33
	66VO	2.12	1.77
Transcription termination factor 2	*FO*	1.17	1.44
	66VO	2.41	1.90
Transcription termination factor, mitochondrial	*FO*	0.98	1.21
	66VO	2.34	2.51
Transducin beta-like 2 protein	*FO*	1.25	1.31
	66VO	1.72	1.57
Transforming growth factor, beta receptor II	*FO*	1.17	1.34
	66VO	1.51	1.58
Transketolase	*FO*	2.92	2.87
	66VO	4.06	4.50
Translation initiation factor eIF-2B subunit delta	*FO*	1.22	1.20
	66VO	1.90	1.46
Translation machinery-associated protein 20	*FO*	1.28	1.30

	66VO	2.55	2.01
Translocation-associated membrane protein 1-like 1	*FO*	1.33	1.24
	66VO	1.94	1.75
Translocon-associated protein subunit delta	*FO*	1.24	1.14
	66VO	1.79	1.46
Transmembrane emp24 domain-containing protein 9	*FO*	1.79	1.51
	66VO	3.33	2.01
Transmembrane protease, serine 2	*FO*	5.49	5.62
	66VO	10.52	8.23
Transmembrane protein 147	*FO*	1.50	1.70
	66VO	3.72	2.85
Transmembrane protein 33	*FO*	1.41	1.67
	66VO	2.75	1.91
Transmembrane protein 93	*FO*	1.32	1.58
	66VO	2.39	2.06
Transmembrane protein C2orf37 homolog	*FO*	1.08	1.16
	66VO	2.31	2.02
Transmembrane protein MGC3196	*FO*	1.62	1.74
	66VO	2.48	2.56
Transposon TX1 uncharacterized 149 kDa protein	*FO*	1.30	1.52
	66VO	4.62	3.64
Trifunctional enzyme subunit alpha, mitochondrial	*FO*	1.83	2.08
	66VO	3.57	3.02
Trifunctional enzyme subunit beta, mitochondrial	*FO*	1.58	1.79
	66VO	2.22	1.94
Tripartite motif protein 29	*FO*	0.40	0.92
	66VO	5.57	5.81
tRNA-dihydrouridine synthase 4-like	*FO*	1.64	1.69
	66VO	3.89	3.06
tRNA-nucleotidyltransferase 1, mitochondrial	*FO*	1.35	1.56
	66VO	2.03	2.15
Tubulin alpha chain	*FO*	1.04	1.18
	66VO	1.89	1.81
Tubulin beta-1 chain	*FO*	1.24	1.35
	66VO	2.29	2.29
Tubulin beta-2 chain	*FO*	1.36	1.61
	66VO	2.89	2.79
Tubulin beta-2C chain	*FO*	1.27	1.45
	66VO	2.49	2.30
Tubulin beta-3 chain	*FO*	2.14	1.51
	66VO	4.95	4.30
Tumor necrosis factor receptor superfamily member 4	*FO*	1.55	1.94
	66VO	4.18	2.58
Tumor necrosis factor receptor superfamily member 5	*FO*	1.27	1.48
	66VO	2.01	1.90
Tumor necrosis factor-inducible protein TSG-6	*FO*	0.44	0.49
	66VO	4.18	9.65
Type I inositol-1,4,5-trisphosphate 5-phosphatase	*FO*	1.87	1.19
	66VO	4.30	3.25
Type II IL-1 receptor	*FO*	3.24	3.27
	66VO	29.53	11.51
Tyrosine-protein kinase BTK	*FO*	1.52	1.36
	66VO	1.95	2.12
Tyrosine-protein phosphatase non-receptor type 12	*FO*	1.22	1.29
	66VO	1.77	1.87
Tyrosyl-tRNA synthetase, cytoplasmic	*FO*	2.43	3.42
	66VO	12.56	7.90
U1 small nuclear ribonucleoprotein A	*FO*	2.01	2.72
	66VO	3.91	2.98

U2 small nuclear ribonucleoprotein A'	*FO*	1.50	2.34
	66VO	2.43	3.17
U3 small nucleolar ribonucleoprotein protein IMP4	*FO*	1.64	1.43
	66VO	2.46	2.24
U3 small nucleolar ribonucleoprotein protein MPP10	*FO*	1.21	1.45
	66VO	2.73	2.33
U4/U6 small nuclear ribonucleoprotein Prp31	*FO*	1.12	1.29
	66VO	1.83	1.72
U4/U6.U5 tri-snRNP-associated protein 2	*FO*	2.56	2.61
	66VO	5.42	4.10
U6 snRNA-associated Sm-like protein LSm2	*FO*	1.06	0.88
	66VO	1.94	1.59
U6 snRNA-associated Sm-like protein LSm3	*FO*	1.39	1.73
	66VO	3.33	2.72
U7 snRNA-associated Sm-like protein LSm10	*FO*	1.13	1.11
	66VO	2.17	1.88
Ubiquinone biosynthesis protein COQ7 homolog	*FO*	1.29	1.49
	66VO	2.03	1.89
Ubiquitin	*FO*	2.13	2.77
	66VO	3.20	5.55
Ubiquitin carboxyl-terminal hydrolase 10	*FO*	1.50	1.69
	66VO	2.12	1.97
Ubiquitin carboxyl-terminal hydrolase 4	*FO*	1.16	1.25
	66VO	1.68	1.48
Ubiquitin carboxyl-terminal hydrolase 5	*FO*	1.56	2.01
	66VO	3.10	2.50
Ubiquitin carboxyl-terminal hydrolase isozyme L3	*FO*	1.73	2.04
	66VO	3.45	2.83
Ubiquitin conjugation factor E4 A	*FO*	1.19	1.44
	66VO	1.99	1.59
Ubiquitin family protein	*FO*	6.93	17.94
	66VO	6.32	9.10
Ubiquitin-conjugating enzyme E2 L3	*FO*	1.10	1.06
	66VO	1.69	1.57
Ubiquitin-conjugating enzyme E2 N	*FO*	1.53	1.83
	66VO	2.98	2.69
Ubiquitin-conjugating enzyme E2 R2	*FO*	2.09	2.16
	66VO	2.54	1.83
Ubiquitin-like modifier-activating enzyme 6	*FO*	1.74	2.11
	66VO	2.82	2.30
Ubiquitin-like protein	*FO*	7.03	5.31
	66VO	7.88	3.07
UCHL5-interacting protein	*FO*	1.29	1.18
	66VO	1.95	1.58
UDP-glucose 6-dehydrogenase	*FO*	1.89	2.33
	66VO	5.05	3.62
UDP-glucuronosyltransferase 2B17	*FO*	1.60	1.80
	66VO	2.48	2.49
UDP-xylose and UDP-N-acetylglucosamine transporter	*FO*	1.33	1.73
	66VO	3.37	2.28
UNC45 homolog A	*FO*	1.53	1.60
	66VO	2.00	2.38
Uncharacterized methyltransferase WBSCR22	*FO*	1.19	1.29
	66VO	1.71	1.56
Uncharacterized potential DNA-binding protein C14orf106	*FO*	2.64	2.93
	66VO	3.00	2.86
Uncharacterized protein At1g14870	*FO*	5.87	10.03
	66VO	14.50	9.24
Uncharacterized protein C12orf5	*FO*	0.98	1.37

	66VO	2.16	1.49
Uncharacterized protein C17orf37	*FO*	1.16	1.06
	66VO	4.78	5.65
Uncharacterized protein C17orf89	*FO*	1.00	1.12
	66VO	1.61	1.80
Uncharacterized protein C18orf37 homolog	*FO*	1.17	1.30
	66VO	2.28	2.04
Uncharacterized protein C19orf60 homolog	*FO*	1.43	1.67
	66VO	2.87	2.26
Uncharacterized protein C1orf156 homolog	*FO*	1.64	1.44
	66VO	2.82	2.70
Uncharacterized protein C1orf93 homolog	*FO*	1.91	2.48
	66VO	1.23	1.69
Uncharacterized protein C20orf72 homolog	*FO*	3.16	3.64
	66VO	8.31	7.64
Uncharacterized protein C21orf45 homolog	*FO*	2.15	2.02
	66VO	2.75	2.37
Uncharacterized protein C21orf59	*FO*	1.28	1.14
	66VO	2.11	1.96
Uncharacterized protein C3orf60 homolog	*FO*	1.25	1.18
	66VO	2.83	2.01
Uncharacterized protein C7orf44	*FO*	2.95	3.36
	66VO	5.39	4.34
Uncharacterized protein C8orf55 homolog	*FO*	2.32	2.93
	66VO	9.96	7.50
Uncharacterized protein C9orf105 homolog	*FO*	1.27	1.48
	66VO	2.34	2.07
Uncharacterized protein KIAA0090	*FO*	1.32	1.60
	66VO	2.80	2.34
Uncharacterized protein KIAA1712 homolog	*FO*	1.33	1.21
	66VO	2.02	1.96
Uncharacterized protein LOC391356 homolog	*FO*	2.15	2.84
	66VO	5.06	3.86
Uncharacterized protein YKR074W	*FO*	1.12	1.20
	66VO	2.22	1.93
UPF0139 protein C19orf56 homolog	*FO*	1.32	1.09
	66VO	4.34	1.77
UPF0287 protein C16orf61 homolog	*FO*	1.31	1.26
	66VO	2.07	1.97
UPF0327 protein C1orf151	*FO*	1.37	1.52
	66VO	2.68	2.38
UPF0351 protein C9orf32 homolog	*FO*	1.31	1.37
	66VO	3.13	2.09
UPF0369 protein C6orf57	*FO*	1.26	1.58
	66VO	2.49	2.62
UPF0384 protein CGI-117 homolog	*FO*	1.15	1.05
	66VO	2.64	1.63
UPF0390 protein zgc136864	*FO*	1.21	1.23
	66VO	2.38	1.90
UPF0436 protein C9orf6 homolog	*FO*	1.07	1.13
	66VO	1.65	1.89
UPF0446 protein C12orf31 homolog	*FO*	1.15	1.29
	66VO	2.18	1.66
UPF0464 protein C15orf44	*FO*	1.31	1.51
	66VO	1.94	1.76
Uracil phosphoribosyltransferase	*FO*	1.08	1.09
	66VO	1.93	1.53
Uridine 5'-monophosphate synthase	*FO*	2.02	2.01
	66VO	3.37	2.93

Uridine phosphorylase 1	*FO*	1.62	2.04
	66VO	2.67	1.81
Valyl-tRNA synthetase	*FO*	1.04	1.08
	66VO	2.18	1.62
Voltage-dependent anion-selective channel protein 2	*FO*	1.48	1.84
	66VO	2.08	2.05
Von willebrand factor type a	*FO*	3.57	2.90
	66VO	5.46	4.26
WD repeat and HMG-box DNA-binding protein 1	*FO*	3.50	3.68
	66VO	6.21	5.61
WD repeat-containing protein 61	*FO*	1.75	1.88
	66VO	2.94	2.45
Zinc finger BED domain-containing protein 1	*FO*	0.43	0.42
	66VO	2.06	1.95
Zinc finger CCCH domain-containing protein 7B	*FO*	1.61	1.73
	66VO	1.45	1.70
Zinc finger CCHC domain-containing protein 10	*FO*	1.24	1.45
	66VO	2.58	2.14
Zinc finger CCHC domain-containing protein 9	*FO*	1.25	1.35
	66VO	1.93	1.80
Zinc finger protein 313	*FO*	1.82	2.04
	66VO	2.55	1.89
Zinc finger protein 554	*FO*	1.03	1.10
	66VO	1.64	1.73
Zinc finger protein 782	*FO*	1.05	1.24
	66VO	1.65	1.63
Zinc finger protein ZPR1	*FO*	1.19	1.28
	66VO	2.48	1.83

Cluster 3: Strong down-regulation (247 genes)

**Putative Id**		**INF$_E$**	**INF$_L$**
Description		INF$_E$	INF$_L$
10-formyltetrahydrofolate dehydrogenase	*FO*	0.32	0.28
	66VO	0.15	0.35
1-O-acylceramide synthase	*FO*	0.30	0.41
	66VO	0.16	0.20
2-acylglycerol O-acyltransferase 2-A	*FO*	0.50	0.13
	66VO	0.09	0.11
2-oxo-4-hydroxy-4-carboxy-5-ureidoimidazoline decarboxylase	*FO*	0.21	0.20
	66VO	0.22	0.25
40S ribosomal protein S5	*FO*	0.36	0.42
	66VO	0.24	0.26
52 kDa Ro protein	*FO*	0.57	0.26
	66VO	0.08	0.17
5'-AMP-activated protein kinase subunit beta-1	*FO*	0.51	0.45
	66VO	0.31	0.38
5-hydroxyisourate hydrolase	*FO*	0.19	0.45
	66VO	0.07	0.26
6-phosphofructo-2-kinase/fructose-2,6-biphosphatase 4	*FO*	0.67	0.83
	66VO	0.29	0.45
Abhydrolase domain-containing protein 12	*FO*	0.48	0.55
	66VO	0.21	0.44
Acid ceramidase	*FO*	0.27	0.37
	66VO	0.22	0.27
Acyl-CoA desaturase	*FO*	0.36	0.39
	66VO	0.26	0.36
Adenylate cyclase type 5	*FO*	0.64	0.48
	66VO	0.19	0.35
ADP-ribosyl cyclase 1	*FO*	0.49	0.40
	66VO	0.25	0.30
Alanine--glyoxylate aminotransferase 2, mitochondrial	*FO*	0.44	0.28
	66VO	0.24	0.27
Alcohol dehydrogenase 1	*FO*	0.24	0.32
	66VO	0.16	0.26
Allantoinase, mitochondrial	*FO*	0.20	0.32
	66VO	0.27	0.26
Alpha 1 collagen type I	*FO*	0.55	0.74
	66VO	0.17	0.55
Alpha-1-antitrypsin-like protein CM55-MS	*FO*	0.11	0.02
	66VO	0.20	0.30
Alpha-tocopherol transfer protein	*FO*	0.34	0.39
	66VO	0.19	0.25
Amine sulfotransferase	*FO*	0.21	0.32
	66VO	0.14	0.19
Aminopeptidase N	*FO*	0.28	0.37
	66VO	0.11	0.26
Angiotensin-converting enzyme	*FO*	0.10	0.12
	66VO	0.02	0.04
Apolipoprotein A-I	*FO*	0.36	0.36
	66VO	0.11	0.21
Apoptosis regulator Bcl-X	*FO*	0.43	0.52
	66VO	0.26	0.30
Aquaporin FA-CHIP	*FO*	0.24	0.34
	66VO	0.11	0.18
Aquaporin-10	*FO*	0.34	0.25
	66VO	0.08	0.33

B(0,+)-type amino acid transporter 1	*FO*	0.20	0.19
	66VO	0.03	0.15
BCL2/adenovirus E1B 19 kDa protein-interacting protein 3	*FO*	0.42	0.32
	66VO	0.29	0.28
Beta,beta-carotene 15,15'-monooxygenase	*FO*	0.25	0.20
	66VO	0.05	0.12
Beta-galactosidase	*FO*	0.50	0.46
	66VO	0.24	0.17
Beta-hexosaminidase alpha chain	*FO*	0.34	0.35
	66VO	0.26	0.36
Betaine--homocysteine S-methyltransferase 1	*FO*	0.46	0.68
	66VO	0.20	0.45
Calsequestrin-1	*FO*	0.13	0.20
	66VO	0.26	0.21
Cannabinoid receptor type 1A	*FO*	0.27	0.33
	66VO	0.21	0.45
Carboxypeptidase inhibitor SmCI	*FO*	0.43	0.33
	66VO	0.28	0.42
Carboxypeptidase O	*FO*	0.21	0.20
	66VO	0.03	0.13
Carnitine palmitoyltransferase 1B	*FO*	0.64	0.57
	66VO	0.24	0.48
Cathepsin B	*FO*	0.37	0.56
	66VO	0.22	0.35
Cathepsin D	*FO*	0.38	0.56
	66VO	0.31	0.35
CB1 cannabinoid receptor	*FO*	0.28	0.32
	66VO	0.19	0.41
Cbp/p300-interacting transactivator 2	*FO*	0.32	0.35
	66VO	0.15	0.13
C-C motif chemokine 20	*FO*	0.29	0.63
	66VO	0.08	0.26
Choline transporter-like protein 4	*FO*	0.43	0.63
	66VO	0.22	0.55
Chordin-like protein	*FO*	0.36	0.36
	66VO	0.18	0.48
Cleft lip and palate transmembrane protein	*FO*	0.49	0.46
	66VO	0.25	0.57
Coagulation factor V	*FO*	0.31	0.45
	66VO	0.46	0.26
Collagen alpha-1(I) chain	*FO*	0.57	0.76
	66VO	0.18	0.61
Collagen alpha-2(I) chain	*FO*	0.51	0.74
	66VO	0.15	0.50
Collagen type IV alpha-3-binding protein	*FO*	0.37	0.32
	66VO	0.16	0.29
Complement C1q tumor necrosis factor-related protein 3	*FO*	0.12	0.15
	66VO	0.01	0.08
Complement C1q-like protein 3	*FO*	0.46	0.56
	66VO	0.14	0.39
Complement C2	*FO*	0.48	0.45
	66VO	0.23	0.36
Complement component q subcomponent-like 4	*FO*	1.10	0.53
	66VO	0.04	0.13
Complement factor H	*FO*	0.51	0.62
	66VO	0.19	0.31
Complement regulator factor H	*FO*	0.22	0.14
	66VO	0.28	0.18
Cubilin	*FO*	0.13	0.26

	66VO	0.06	0.06
Cullin-associated NEDD8-dissociated protein 1	*FO*	0.80	0.52
	66VO	0.35	0.37
Cystatin	*FO*	0.32	0.36
	66VO	0.27	0.26
Cysteine sulfinic acid decarboxylase	*FO*	0.44	0.38
	66VO	0.26	0.37
Cytochrome P450 1A1	*FO*	0.33	0.26
	66VO	0.11	0.41
Cytochrome P450 2A10	*FO*	0.18	0.20
	66VO	0.11	0.16
Cytochrome P450 2C30	*FO*	0.38	0.33
	66VO	0.22	0.33
Cytochrome P450 2F1	*FO*	0.36	0.24
	66VO	0.36	0.38
Cytochrome P450 2J5	*FO*	0.22	0.19
	66VO	0.09	0.18
Cytochrome P450 2J6	*FO*	0.23	0.20
	66VO	0.02	0.12
Cytochrome P450 3A30	*FO*	0.48	0.32
	66VO	0.20	0.31
Cytochrome P450 3A40	*FO*	1.65	0.67
	66VO	0.08	0.15
Cytochrome P450 3A56	*FO*	0.71	0.41
	66VO	0.24	0.25
Cytokine receptor family member b9	*FO*	0.33	0.53
	66VO	0.26	0.21
Cytosolic non-specific dipeptidase	*FO*	0.57	0.59
	66VO	0.30	0.35
D-amino-acid oxidase	*FO*	0.38	0.31
	66VO	0.19	0.29
D-dopachrome decarboxylase	*FO*	0.55	0.39
	66VO	0.33	0.35
Deoxyribonuclease-1	*FO*	0.16	0.21
	66VO	0.10	0.30
Desmoplakin	*FO*	0.48	0.76
	66VO	0.28	0.71
Diacylglycerol O-acyltransferase 2	*FO*	0.52	0.08
	66VO	0.20	0.29
Digestive cysteine proteinase 1	*FO*	0.33	0.40
	66VO	0.23	0.28
Dimethylaniline monooxygenase [N-oxide-forming] 5	*FO*	0.65	0.79
	66VO	0.18	0.18
Dimethylglycine dehydrogenase, mitochondrial	*FO*	0.44	0.55
	66VO	0.29	0.62
Dipeptidase 1	*FO*	0.22	0.50
	66VO	0.07	0.17
Dipeptidyl peptidase 4	*FO*	0.44	0.49
	66VO	0.17	0.34
DNA-binding protein inhibitor ID-4	*FO*	0.40	0.38
	66VO	0.19	0.41
Ectonucleotide pyrophosphatase/phosphodiesterase 6	*FO*	0.17	0.22
	66VO	0.06	0.08
Ectonucleotide pyrophosphatase/phosphodiesterase family member 7	*FO*	0.14	0.07
	66VO	0.04	0.06
Epidermal growth factor receptor kinase substrate 8-like protein 2	*FO*	0.41	0.44
	66VO	0.26	0.46
Epididymal secretory protein E1	*FO*	0.37	0.53
	66VO	0.29	0.33

Erythropoietin receptor	*FO*	0.51	0.43
	66VO	0.09	0.15
Estrogen receptor beta	*FO*	0.23	0.18
	66VO	0.08	0.25
Eukaryotic translation initiation factor 4E type 3	*FO*	0.47	0.43
	66VO	0.15	0.26
Fam83d-b protein	*FO*	0.43	0.41
	66VO	0.20	0.30
Fatty acid-binding protein, intestinal	*FO*	0.11	0.03
	66VO	0.02	0.05
Fatty-acid amide hydrolase 1	*FO*	0.21	0.22
	66VO	0.07	0.14
F-box only protein 25	*FO*	0.35	0.27
	66VO	0.15	0.15
F-box only protein 8	*FO*	0.38	0.49
	66VO	0.31	0.32
Ferritin, heavy subunit	*FO*	0.41	0.43
	66VO	0.23	0.27
Fibroblast growth factor receptor 4	*FO*	0.49	0.51
	66VO	0.24	0.44
Flavin reductase	*FO*	0.52	0.49
	66VO	0.22	0.36
Formimidoyltransferase-cyclodeaminase	*FO*	0.70	0.44
	66VO	0.16	0.28
Fucolectin-1	*FO*	0.27	0.41
	66VO	0.03	0.12
Galactocerebrosidase	*FO*	0.35	0.49
	66VO	0.21	0.37
Galanin	*FO*	0.09	0.05
	66VO	0.17	0.22
Gamma-aminobutyric acid receptor-associated protein-like 1	*FO*	0.33	0.33
	66VO	0.25	0.30
Gamma-glutamyl hydrolase	*FO*	0.18	0.29
	66VO	0.11	0.18
Gamma-glutamyltranspeptidase 1	*FO*	0.20	0.33
	66VO	0.09	0.12
Glucokinase	*FO*	0.44	0.45
	66VO	0.29	0.36
Glucosylceramidase	*FO*	0.29	0.35
	66VO	0.11	0.21
Glutamyl aminopeptidase	*FO*	0.28	0.27
	66VO	0.04	0.11
Glycerate kinase	*FO*	0.35	0.34
	66VO	0.16	0.25
Glycerol-3-phosphate dehydrogenase [NAD+], cytoplasmic	*FO*	0.71	0.73
	66VO	0.23	0.45
Glycogenin-1	*FO*	0.33	0.38
	66VO	0.36	0.58
Granulins	*FO*	0.36	0.59
	66VO	0.22	0.28
Growth factor receptor-bound protein 14	*FO*	0.53	0.51
	66VO	0.19	0.40
GTPase IMAP family member 4	*FO*	0.33	0.31
	66VO	0.04	0.18
GTPase IMAP family member 7	*FO*	0.51	0.44
	66VO	0.36	0.46
Guanidinoacetate N-methyltransferase	*FO*	0.39	0.38
	66VO	0.16	0.41
Heat-stable enterotoxin receptor	*FO*	0.37	0.38

	66VO	0.31	0.72
Hepatocyte nuclear factor 4-gamma	*FO*	0.48	0.44
	66VO	0.12	0.26
HMG box-containing protein 1	*FO*	0.50	0.45
	66VO	0.31	0.39
Homologue of sarcophaga proteinase	*FO*	0.31	0.36
	66VO	0.16	0.20
Hydroxyacid oxidase 2	*FO*	0.43	0.55
	66VO	0.14	0.39
Hydroxysteroid 17-beta dehydrogenase 6	*FO*	0.27	0.24
	66VO	0.17	0.20
Integral membrane protein GPR137	*FO*	0.50	0.48
	66VO	0.29	0.35
Iodothyronine deiodinase 1	*FO*	0.23	0.16
	66VO	0.10	0.19
Iron/zinc purple acid phosphatase-like protein	*FO*	0.26	0.36
	66VO	0.11	0.19
Kelch domain-containing protein 8A	*FO*	0.53	0.37
	66VO	0.29	0.52
Kelch-like protein 26	*FO*	0.50	0.41
	66VO	0.30	0.36
Kynurenine/alpha-aminoadipate aminotransferase mitochondrial	*FO*	0.33	0.39
	66VO	0.26	0.27
Lactoperoxidase	*FO*	0.76	0.13
	66VO	0.30	0.41
Lactose-binding lectin l-2	*FO*	0.48	0.39
	66VO	0.11	0.25
Lectin	*FO*	0.13	0.17
	66VO	0.04	0.16
Legumain	*FO*	0.39	0.49
	66VO	0.33	0.38
Low affinity cationic amino acid transporter 2	*FO*	0.33	0.39
	66VO	0.14	0.34
Major antigen	*FO*	0.23	0.35
	66VO	0.07	0.22
Major histocompatibility complex class Ia chain	*FO*	0.59	0.42
	66VO	0.29	0.31
MAM domain-containing protein 2	*FO*	0.66	0.92
	66VO	0.16	0.57
Mannose-binding protein C	*FO*	0.17	0.50
	66VO	0.06	0.16
Mantle gene 8	*FO*	0.18	0.32
	66VO	0.05	0.14
Mast cell carboxypeptidase A	*FO*	0.38	0.60
	66VO	0.11	0.27
Meprin A subunit alpha	*FO*	0.20	0.16
	66VO	0.05	0.05
Meprin A subunit beta	*FO*	0.19	0.22
	66VO	0.05	0.11
Metallothionein	*FO*	0.21	0.39
	66VO	0.10	0.13
Methionine-R-sulfoxide reductase B2	*FO*	0.18	0.17
	66VO	0.06	0.14
Microtubule-associated protein RP/EB family member 3	*FO*	0.35	0.35
	66VO	0.17	0.37
Moesin	*FO*	0.50	0.40
	66VO	0.21	0.41
MOSC domain-containing protein 1, mitochondrial	*FO*	0.30	0.25
	66VO	0.15	0.29

Multidrug and toxin extrusion protein 1	*FO*	0.57	0.65
	66VO	0.20	0.59
Musculoskeletal embryonic nuclear protein 1	*FO*	0.50	0.49
	66VO	0.22	0.54
Myocilin	*FO*	0.26	0.23
	66VO	0.24	0.18
Myosin heavy chain, fast skeletal muscle	*FO*	0.25	0.46
	66VO	0.08	0.17
N-acetylated-alpha-linked acidic dipeptidase-like protein	*FO*	0.28	0.31
	66VO	0.06	0.17
NADH dehydrogenase [ubiquinone] flavoprotein 1, mitochondrial	*FO*	0.41	0.47
	66VO	0.20	0.26
Nanos homolog 1	*FO*	0.21	0.29
	66VO	0.18	0.74
Neurabin-1	*FO*	0.45	0.52
	66VO	0.14	0.39
Neutral alpha-glucosidase C	*FO*	0.85	0.78
	66VO	0.15	0.28
Neutral and basic amino acid transport protein rBAT	*FO*	0.25	0.23
	66VO	0.06	0.11
Non-syndromic hearing impairment protein 5	*FO*	0.66	0.67
	66VO	0.23	0.30
Nostrin	*FO*	0.50	0.56
	66VO	0.27	0.54
Novel immune-type receptor 4	*FO*	0.52	0.73
	66VO	0.25	0.46
N-terminal EF-hand calcium-binding protein 1	*FO*	0.28	0.24
	66VO	0.11	0.25
NUAK family SNF1-like kinase 2	*FO*	0.59	0.49
	66VO	0.25	0.33
Nuclear protein 1	*FO*	0.49	0.37
	66VO	0.19	0.24
Nuclear receptor subfamily 1 group D member 1	*FO*	0.07	0.19
	66VO	0.11	0.19
Occludin	*FO*	0.40	0.51
	66VO	0.15	0.31
Ovary-specific c1q-like factor	*FO*	0.23	0.35
	66VO	0.08	0.24
Oxysterol-binding protein-related protein 3	*FO*	0.45	0.61
	66VO	0.37	0.33
Pancreatic secretory trypsin inhibitor	*FO*	0.26	0.12
	66VO	0.15	0.23
Para-nitrobenzyl esterase	*FO*	0.41	0.48
	66VO	0.31	0.51
PDZ domain-containing protein 1	*FO*	0.14	0.30
	66VO	0.10	0.22
Pentraxin fusion protein	*FO*	0.46	0.45
	66VO	0.42	0.39
Peptidase d	*FO*	0.40	0.38
	66VO	0.22	0.37
Phospholipase A2, acidic 1	*FO*	0.03	0.01
	66VO	0.06	0.02
Phospholipase D1	*FO*	0.55	0.54
	66VO	0.24	0.32
Placental protein 11	*FO*	0.49	0.40
	66VO	0.34	0.43
Plexin-A4	*FO*	0.51	0.44
	66VO	0.31	0.59
Podocan	*FO*	0.45	0.57

	66VO	0.17	0.56
Poly(rC)-binding protein 3	*FO*	0.35	0.38
	66VO	0.26	0.36
Probable 10-formyltetrahydrofolate dehydrogenase ALDH1L2	*FO*	0.32	0.21
	66VO	0.13	0.28
Probable glutamate receptor	*FO*	0.45	0.32
	66VO	0.16	0.30
Probable G-protein coupled receptor 144	*FO*	0.37	0.46
	66VO	0.29	0.35
Probable N-acetyltransferase CML1	*FO*	0.31	0.37
	66VO	0.21	0.22
Probable serine carboxypeptidase CPVL	*FO*	0.28	0.38
	66VO	0.12	0.16
Proline rich 5	*FO*	0.39	0.35
	66VO	0.16	0.27
Protein aidB	*FO*	0.54	0.47
	66VO	0.25	0.40
Protein CREG2	*FO*	0.54	0.50
	66VO	0.28	0.38
Protein Z-dependent protease inhibitor	*FO*	0.67	0.62
	66VO	0.15	0.31
Protocadherin LKC	*FO*	0.38	0.42
	66VO	0.14	0.31
Protocadherin-like wing polarity protein stan	*FO*	0.69	0.92
	66VO	0.16	0.41
PTB domain-containing engulfment adapter protein 1	*FO*	0.54	0.51
	66VO	0.21	0.39
Putative phospholipase B-like 2	*FO*	0.36	0.36
	66VO	0.21	0.25
Putative protein 2	*FO*	0.37	0.45
	66VO	0.28	0.35
Radixin	*FO*	0.56	0.48
	66VO	0.32	0.62
Rap guanine nucleotide exchange factor 3	*FO*	0.42	0.53
	66VO	0.15	0.29
RAS guanyl-releasing protein 2	*FO*	0.38	0.52
	66VO	0.26	0.38
Ras-related protein Rab-20	*FO*	0.34	0.27
	66VO	0.11	0.17
Ras-related protein Rab-9A	*FO*	0.57	0.59
	66VO	0.29	0.47
Receptor-type tyrosine-protein phosphatase delta	*FO*	0.31	0.32
	66VO	0.13	0.29
Regucalcin	*FO*	0.43	0.55
	66VO	0.29	0.33
Retinitis pigmentosa 1 homolog	*FO*	0.48	0.28
	66VO	0.26	0.39
Retinol dehydrogenase 8	*FO*	0.51	0.44
	66VO	0.18	0.32
Scavenger receptor class B member 1	*FO*	0.31	0.18
	66VO	0.14	0.19
Second macrophage colony-stimulating factor	*FO*	0.62	0.54
	66VO	0.21	0.48
Secretagogin	*FO*	0.46	0.53
	66VO	0.23	0.35
Selenoprotein Pa	*FO*	0.35	0.32
	66VO	0.25	0.31
Selenoprotein Pb	*FO*	0.27	0.18
	66VO	0.28	0.44

Seprase	*FO*	0.43	0.40
	66VO	0.12	0.27
Serine hydroxymethyltransferase, cytosolic	*FO*	0.33	0.39
	66VO	0.24	0.37
Serine hydroxymethyltransferase, mitochondrial	*FO*	0.36	0.26
	66VO	0.12	0.20
Serine incorporator 1	*FO*	0.54	0.61
	66VO	0.29	0.37
Serine/threonine-protein kinase ULK1	*FO*	0.45	0.46
	66VO	0.12	0.21
Serine--pyruvate aminotransferase, mitochondrial	*FO*	0.53	0.44
	66VO	0.19	0.42
Serotransferrin	*FO*	0.34	0.40
	66VO	0.18	0.27
SET and MYND domain-containing protein 1	*FO*	0.44	0.52
	66VO	0.26	0.34
Sickle tail protein	*FO*	0.53	0.50
	66VO	0.25	0.46
Sodium-dependent neutral amino acid transporter B(0	*FO*	0.09	0.07
	66VO	0.04	0.03
Solute carrier family 12 member 2	*FO*	0.40	0.46
	66VO	0.14	0.43
Solute carrier family 13 member 2	*FO*	0.14	0.12
	66VO	0.22	0.14
Solute carrier family 13 member 5	*FO*	0.27	0.42
	66VO	0.16	0.25
Solute carrier family 22 member 7	*FO*	0.31	0.52
	66VO	0.27	0.55
Solute carrier family 25 member 42	*FO*	0.37	0.35
	66VO	0.15	0.22
Solute carrier family 5 (sodium glucose transporter) member 11	*FO*	0.38	0.42
	66VO	0.07	0.24
Solute carrier family member 19	*FO*	0.41	0.71
	66VO	0.19	0.36
Sperm acrosome associated 4	*FO*	0.50	0.19
	66VO	0.28	0.22
Sphingomyelin phosphodiesterase acid lysosomal	*FO*	0.19	0.28
	66VO	0.12	0.18
Sporulation-specific protein 15	*FO*	0.25	0.34
	66VO	0.04	0.16
Sulfotransferase family cytosolic 2B member 1	*FO*	0.64	0.99
	66VO	0.20	0.40
Sushi domain-containing protein 2	*FO*	0.27	0.36
	66VO	0.07	0.16
Synaptonemal complex protein 1	*FO*	0.43	0.40
	66VO	0.23	0.41
T-complex protein 11-like 2	*FO*	0.55	0.51
	66VO	0.16	0.27
Tenascin	*FO*	0.81	0.83
	66VO	0.14	0.53
Tetraspanin-3	*FO*	0.43	0.34
	66VO	0.31	0.37
Thrombospondin-4	*FO*	0.68	0.85
	66VO	0.22	0.60
Tissue-type plasminogen activator	*FO*	0.31	0.29
	66VO	0.32	0.27
Transmembrane protein 106B	*FO*	0.41	0.54
	66VO	0.29	0.30
Transmembrane protein 45B	*FO*	0.51	1.01

	66VO	0.26	0.27
Transmembrane protein 59	*FO*	0.47	0.46
	66VO	0.30	0.46
Transposable element Tc1 transposase	*FO*	0.62	0.52
	66VO	0.27	0.46
Transposase domain-containing protein	*FO*	0.47	0.47
	66VO	0.18	0.44
Uncoupling protein 1	*FO*	0.19	0.11
	66VO	0.04	0.08
Uncharacterized protein C11orf51 homolog	*FO*	0.40	0.33
	66VO	0.17	0.22
Uncharacterized protein C15orf27	*FO*	0.32	0.32
	66VO	0.24	0.36
Uncharacterized protein C5orf4	*FO*	0.28	0.22
	66VO	0.11	0.14
Uncharacterized protein C9orf61	*FO*	0.48	0.34
	66VO	0.34	0.34
Xaa-Pro aminopeptidase 2	*FO*	0.29	0.38
	66VO	0.06	0.17
Xaa-Pro dipeptidase	*FO*	0.50	0.42
	66VO	0.14	0.24
Zinc finger protein 678	*FO*	0.66	0.89
	66VO	0.19	0.43
Zona pellucida protein x	*FO*	0.59	0.53
	66VO	0.16	0.42
Zymogen granule membrane protein 16	*FO*	0.69	0.55
	66VO	0.25	0.45

Cluster 4: Down-regulation (1,000 genes)

Putative Id		INFE	INFL
[Pyruvate dehydrogenase [lipoamide]] kinase isozyme 1, mitochondrial	*FO*	0.65	0.61
	66VO	0.60	0.66
116 kDa U5 small nuclear ribonucleoprotein component	*FO*	0.72	0.60
	66VO	0.66	0.61
14 kda apolipoprotein	*FO*	0.68	0.53
	66VO	0.63	0.54
1-acyl-sn-glycerol-3-phosphate acyltransferase zeta	*FO*	0.71	0.91
	66VO	0.31	0.50
1-phosphatidylinositol-4,5-bisphosphate phosphodiesterase delta-4	*FO*	0.57	0.63
	66VO	0.28	0.52
26S proteasome non-ATPase regulatory subunit 9	*FO*	0.89	0.95
	66VO	0.89	0.76
28S ribosomal protein S16, mitochondrial	*FO*	0.75	0.78
	66VO	0.68	0.70
3-hydroxybutyrate dehydrogenase type 2	*FO*	0.86	0.68
	66VO	0.40	0.65
3-oxo-5-beta-steroid 4-dehydrogenase	*FO*	0.71	0.69
	66VO	0.18	0.40
3-phosphoinositide-dependent protein kinase 1	*FO*	0.88	0.88
	66VO	0.82	0.76
40S ribosomal protein S13	*FO*	0.99	1.06
	66VO	1.31	1.13
4-aminobutyrate aminotransferase, mitochondrial	*FO*	0.56	0.51
	66VO	0.48	0.48
52 kDa repressor of the inhibitor of the protein kinase	*FO*	0.76	0.88
	66VO	0.49	0.63
52 kDa ro protein	*FO*	0.63	0.42
	66VO	0.24	0.33
5-aminolevulinate synthase, erythroid-specific, mitochondrial	*FO*	0.56	0.76
	66VO	0.46	0.59
5'-AMP-activated protein kinase subunit gamma-2	*FO*	0.56	0.59
	66VO	0.43	0.52
5-formyltetrahydrofolate cyclo-ligase	*FO*	0.78	0.79
	66VO	0.45	0.58
5'-nucleotidase domain-containing protein 2	*FO*	0.83	0.65
	66VO	0.31	0.44
60S ribosomal protein L15	*FO*	0.76	0.74
	66VO	0.59	0.36
60S ribosomal protein L38	*FO*	0.55	0.61
	66VO	0.52	0.51
60S ribosomal protein L7	*FO*	1.03	0.93
	66VO	0.47	0.71
6PF-2-K/Fru-2,6-P2ASE liver/muscle isozymes	*FO*	0.74	0.71
	66VO	0.34	0.53
6-pyruvoyl tetrahydrobiopterin synthase	*FO*	0.76	0.75
	66VO	0.40	0.39
Absent in melanoma 1 protein	*FO*	0.66	0.65
	66VO	0.27	0.53
Acetolactate synthase-like protein	*FO*	0.99	1.13
	66VO	0.47	1.24
Actin, alpha skeletal muscle	*FO*	0.57	0.65
	66VO	0.18	0.76
Actin-related protein 10	*FO*	0.79	0.88
	66VO	0.58	0.73
Adenosine deaminase	*FO*	0.70	0.60

	66VO	0.41	0.57
Adiponectin receptor protein 1	*FO*	0.75	0.88
	66VO	0.57	0.63
ADP-ribosylation factor 4	*FO*	1.10	1.21
	66VO	0.19	0.22
ADP-ribosylation factor-like protein 16	*FO*	0.49	0.40
	66VO	0.42	0.61
ADP-ribosylation factor-like protein 5A	*FO*	0.73	0.79
	66VO	0.77	0.68
ADP-ribosylation factor-like protein 8B	*FO*	0.53	0.47
	66VO	0.36	0.39
AKT-interacting protein	*FO*	0.70	0.62
	66VO	0.49	0.56
Alcohol dehydrogenase class-3	*FO*	0.82	0.58
	66VO	0.47	0.52
Aldehyde dehydrogenase family 8 member A1	*FO*	1.70	1.63
	66VO	0.84	0.85
Aldehyde dehydrogenase, mitochondrial	*FO*	0.66	0.62
	66VO	0.34	0.43
Aldehyde oxidase	*FO*	0.50	0.55
	66VO	0.26	0.37
Alkylated DNA repair protein alkB homolog 5	*FO*	0.78	0.74
	66VO	0.74	0.77
Alpha-(1,3)-fucosyltransferase	*FO*	0.70	0.76
	66VO	0.55	0.67
Alpha-1-antitrypsin homolog	*FO*	0.52	0.54
	66VO	0.35	0.63
Alpha-1-inhibitor 3	*FO*	0.74	0.81
	66VO	0.34	0.78
Alpha-1-syntrophin	*FO*	0.68	0.53
	66VO	0.48	0.56
Alpha-2 adrenergic receptor	*FO*	0.51	0.52
	66VO	0.51	0.52
Alpha-2-HS-glycoprotein	*FO*	0.36	0.29
	66VO	0.27	0.40
Alpha-2-macroglobulin	*FO*	0.67	0.72
	66VO	0.62	0.64
Alpha-2-macroglobulin-like protein 1	*FO*	0.66	0.60
	66VO	0.77	0.91
Alpha-actinin-2	*FO*	0.84	0.58
	66VO	0.32	0.45
Alpha-crystallin B chain	*FO*	1.00	0.88
	66VO	0.24	0.45
Alpha-globin transcription factor CP2	*FO*	0.74	0.68
	66VO	0.51	0.65
Alpha-N-acetylgalactosaminidase	*FO*	0.70	0.78
	66VO	0.61	0.61
Aminoacylase-1A	*FO*	0.52	0.51
	66VO	0.27	0.40
Ammonium transporter Rh type B	*FO*	0.60	0.81
	66VO	0.24	0.82
Ancient ubiquitous protein 1	*FO*	0.66	0.65
	66VO	0.58	0.56
Angiopoietin-related protein 4	*FO*	1.00	0.71
	66VO	0.42	0.26
Ankib1 protein	*FO*	0.59	0.51
	66VO	0.30	0.39
Ankyrin repeat and FYVE domain-containing protein 1	*FO*	0.63	0.71
	66VO	0.42	0.51

Ankyrin repeat and SOCS box protein 13	*FO*	0.93	0.87
	66VO	0.59	0.66
Ankyrin repeat and SOCS box protein 15	*FO*	0.35	0.27
	66VO	0.68	0.84
Ankyrin repeat and SOCS box protein 5	*FO*	0.58	0.42
	66VO	0.35	0.40
Ankyrin repeat domain-containing protein 12	*FO*	0.62	0.68
	66VO	0.61	0.70
Annexin A13	*FO*	0.61	0.64
	66VO	0.43	0.64
Annexin A5	*FO*	0.85	0.80
	66VO	0.62	0.59
AP-1 complex subunit gamma-1	*FO*	0.75	0.75
	66VO	0.58	0.79
AP1 subunit gamma-binding protein 1	*FO*	0.68	0.67
	66VO	0.68	0.59
Apolipoprotein A-I-binding protein	*FO*	0.45	0.43
	66VO	0.38	0.50
Apolipoprotein B-100	*FO*	1.30	1.64
	66VO	1.66	1.38
Apoptosis regulatory protein Siva	*FO*	0.78	0.86
	66VO	0.42	0.58
Apoptosis-related protein 3	*FO*	0.55	0.68
	66VO	0.35	0.39
Apoptosis-stimulating of p53 protein 1	*FO*	0.67	0.64
	66VO	0.64	0.59
Apoptotic chromatin condensation inducer in the nucleus	*FO*	0.74	0.73
	66VO	0.68	0.77
Arachidonate 12-lipoxygenase, 12S-type	*FO*	0.65	0.60
	66VO	0.48	0.51
ARF GTPase-activating protein GIT2	*FO*	0.78	0.82
	66VO	0.51	0.83
Arfaptin-1	*FO*	0.67	0.73
	66VO	0.70	0.76
Arrestin domain-containing protein 3	*FO*	0.91	0.79
	66VO	0.38	0.37
Aryl hydrocarbon receptor nuclear translocator-like protein 1	*FO*	0.38	0.34
	66VO	0.59	0.64
Aspartoacylase	*FO*	0.59	0.67
	66VO	0.26	0.39
Aspartyl-tRNA synthetase, cytoplasmic	*FO*	0.88	0.76
	66VO	0.60	0.62
Ataxin-2	*FO*	0.59	0.76
	66VO	0.58	0.71
ATP synthase-coupling factor 6, mitochondrial	*FO*	0.91	0.84
	66VO	0.34	0.55
ATPase inhibitor, mitochondrial	*FO*	0.59	0.56
	66VO	0.35	0.46
Atp-binding sub-family c (cftr mrp) member 4	*FO*	0.66	0.66
	66VO	0.50	0.47
ATP-binding sub-family f member 3	*FO*	0.66	0.54
	66VO	0.69	0.66
ATP-dependent RNA helicase DDX42	*FO*	0.65	0.37
	66VO	0.77	0.76
Baculoviral IAP repeat-containing protein 4	*FO*	0.90	0.93
	66VO	0.72	0.65
Basic helix-loop-helix domain-containing protein KIAA2018	*FO*	0.63	0.59
	66VO	0.47	0.65
Basic leucine zipper and W2 domain-containing protein 1	*FO*	0.85	0.83

	66VO	0.60	0.69
Basic leucine zipper and W2 domain-containing protein 1-A	*FO*	0.88	0.78
	66VO	0.65	0.69
Basigin	*FO*	0.74	0.80
	66VO	0.74	0.70
Bc003940 protein	*FO*	0.69	0.69
	66VO	0.59	0.62
Bcl-2-related ovarian killer protein homolog A	*FO*	0.81	1.00
	66VO	0.48	0.70
Beta-2 adrenergic receptor	*FO*	0.60	0.55
	66VO	0.36	0.32
Beta-2-microglobulin	*FO*	0.70	0.70
	66VO	0.63	0.61
Beta-galactosidase-1-like protein 2	*FO*	0.67	0.72
	66VO	0.36	0.61
Beta-soluble NSF attachment protein	*FO*	0.37	0.31
	66VO	0.33	0.35
Beta-synuclein	*FO*	0.56	0.45
	66VO	0.63	0.57
Beta-type platelet-derived growth factor receptor	*FO*	0.73	0.76
	66VO	0.49	0.60
Beta-ureidopropionase	*FO*	0.56	0.54
	66VO	0.27	0.49
Bhlh protein dec1b	*FO*	0.63	0.60
	66VO	0.31	0.49
Biogenesis of lysosome-related organelles complex-1 subunit 3	*FO*	0.66	0.71
	66VO	0.48	0.51
Biotinidase	*FO*	0.63	0.47
	66VO	0.38	0.44
Bone morphogenetic protein 2	*FO*	0.54	0.45
	66VO	0.75	0.63
Brain-specific serine protease 4	*FO*	0.52	1.45
	66VO	0.41	1.89
BRO1 domain-containing protein BROX	*FO*	0.63	0.64
	66VO	0.38	0.45
Bromodomain adjacent to zinc finger domain protein 2B	*FO*	0.62	0.65
	66VO	0.62	0.70
BSD domain-containing protein 1	*FO*	0.74	0.66
	66VO	0.33	0.35
BTB/POZ domain-containing protein KCTD13	*FO*	0.69	0.82
	66VO	0.67	0.57
BTB/POZ domain-containing protein KCTD14	*FO*	0.71	0.90
	66VO	0.36	0.52
Butyrate response factor 1	*FO*	0.74	0.72
	66VO	0.52	0.67
Butyrophilin subfamily 2 member A2	*FO*	0.48	0.47
	66VO	0.36	0.55
Calcium and integrin-binding protein 1	*FO*	0.58	0.86
	66VO	0.35	0.52
Calcium-regulated heat stable protein 1	*FO*	0.52	0.55
	66VO	0.64	0.35
Calmodulin	*FO*	0.61	0.67
	66VO	0.45	0.61
Calpain-9	*FO*	0.69	0.65
	66VO	0.56	0.56
cAMP and cAMP-inhibited cGMP 3',5'-cyclic phosphodiesterase 10A	*FO*	0.73	0.63
	66VO	0.55	0.56
cAMP response element-binding protein	*FO*	0.62	0.72
	66VO	0.62	0.65

cAMP responsive element binding protein 3-like 3	*FO*	0.61	0.74
	66VO	0.41	0.38
cAMP-responsive element-binding protein-like 2	*FO*	0.70	0.71
	66VO	0.51	0.53
CAP-Gly domain-containing linker protein 4	*FO*	0.72	0.74
	66VO	0.55	0.55
Caprin-1	*FO*	0.75	0.84
	66VO	0.71	0.72
Carbohydrate sulfotransferase 14	*FO*	0.60	0.60
	66VO	0.55	0.61
Carboxypeptidase D	*FO*	0.63	0.66
	66VO	0.50	0.54
Carboxypeptidase Z	*FO*	0.43	0.41
	66VO	0.26	0.34
Carboxy-terminal domain RNA polymerase II polypeptide A small phospha	*FO*	0.53	0.48
	66VO	0.29	0.45
Carnitine O-acetyltransferase	*FO*	0.72	0.72
	66VO	0.62	0.74
Carnitine O-palmitoyltransferase I, liver isoform	*FO*	0.53	0.69
	66VO	0.40	0.64
Caspase-3	*FO*	0.67	0.64
	66VO	0.67	0.77
Catalase	*FO*	0.51	0.49
	66VO	0.40	0.53
Cathepsin F	*FO*	0.55	0.49
	66VO	0.35	0.34
C-C chemokine receptor type 7	*FO*	0.71	0.85
	66VO	0.34	0.72
C-C motif chemokine 25	*FO*	0.65	0.82
	66VO	0.52	0.60
CD63 antigen	*FO*	0.69	0.82
	66VO	0.53	0.57
CDGSH iron sulfur domain-containing protein 1	*FO*	0.79	0.80
	66VO	0.72	0.71
CDH1-D	*FO*	0.52	0.55
	66VO	0.52	0.64
Cdkn2a interacting protein	*FO*	0.59	0.61
	66VO	0.56	0.49
Cell cycle control protein 50A	*FO*	0.48	0.49
	66VO	0.24	0.35
Cell cycle progression protein 1	*FO*	0.48	0.51
	66VO	0.15	0.33
Cell division cycle 5-related protein	*FO*	0.67	0.65
	66VO	0.75	0.73
Cell division cycle and apoptosis regulator protein 1	*FO*	0.73	0.64
	66VO	0.48	0.77
Cell division cycle protein 16 homolog	*FO*	0.87	0.90
	66VO	1.23	1.19
Cell surface	*FO*	0.64	0.61
	66VO	0.53	0.61
Centrosomal protein of 290 kDa	*FO*	0.57	0.57
	66VO	0.49	0.50
Ceroid-lipofuscinosis neuronal protein 5	*FO*	0.55	0.70
	66VO	0.31	0.44
Cg14073- isoform a	*FO*	0.76	0.53
	66VO	0.68	0.77
Ch076 protein	*FO*	0.93	0.98
	66VO	0.87	1.14
Charged multivesicular body protein 1b	*FO*	0.65	0.65

	66VO	0.47	0.49
Charged multivesicular body protein 5	*FO*	0.75	0.83
	66VO	0.73	0.69
Chemokine receptor-like 1	*FO*	0.80	0.79
	66VO	0.70	0.82
Chloride channel protein 7	*FO*	0.47	0.51
	66VO	0.30	0.34
Chloride intracellular channel protein 2	*FO*	0.69	0.72
	66VO	0.50	0.64
Chloride intracellular channel protein 4	*FO*	0.52	0.55
	66VO	0.51	0.55
Choline transporter-like protein 2	*FO*	0.62	0.73
	66VO	0.44	0.58
Chromobox protein homolog 8	*FO*	0.89	0.85
	66VO	0.54	0.61
Chronic lymphocytic leukemia deletion region gene 6 protein homolog	*FO*	0.68	0.72
	66VO	0.63	0.56
Ciliary neurotrophic factor receptor alpha	*FO*	0.75	0.61
	66VO	0.33	0.49
Cingulin-like protein 1	*FO*	0.54	0.59
	66VO	0.46	0.59
Class I histocompatibility antigen, F10 alpha chain	*FO*	0.03	0.49
	66VO	0.09	2.60
Claudin domain containing 1	*FO*	0.68	0.76
	66VO	0.66	0.73
Cleavage stimulation factor 77 kDa subunit	*FO*	0.86	0.85
	66VO	0.80	0.91
CLIP-associating protein 2	*FO*	0.70	0.68
	66VO	0.64	0.69
CMP-sialic acid transporter	*FO*	0.65	0.69
	66VO	0.43	0.45
Coagulation factor IX	*FO*	0.25	0.32
	66VO	0.23	0.27
Coagulation factor X	*FO*	0.49	0.49
	66VO	0.36	0.51
Coagulation factor XI	*FO*	0.67	0.68
	66VO	0.63	0.70
Coagulation factor XIII B chain	*FO*	0.82	0.77
	66VO	0.41	0.53
Coatomer subunit zeta-2	*FO*	0.54	0.54
	66VO	0.40	0.38
Coiled-coil domain-containing protein 41	*FO*	0.77	0.77
	66VO	0.62	0.70
Coiled-coil domain-containing protein 47	*FO*	0.54	0.61
	66VO	0.60	0.73
Coiled-coil domain-containing protein 64A	*FO*	0.85	0.94
	66VO	0.44	0.47
Cold-inducible RNA-binding protein	*FO*	0.76	0.45
	66VO	0.53	0.63
Collagen alpha-1(II) chain	*FO*	0.63	0.75
	66VO	0.33	0.60
Collagen alpha-1(VI) chain	*FO*	0.78	0.84
	66VO	0.29	0.77
Collagen alpha-2(VI	*FO*	0.68	0.78
	66VO	0.23	0.68
COMM domain-containing protein 2	*FO*	0.52	0.54
	66VO	0.46	0.42
Complement C1q-like protein 2	*FO*	0.77	0.73
	66VO	0.27	0.39

Complement c1q-like protein 4	*FO*	0.66	0.78
	66VO	0.37	0.43
Complement C3-1	*FO*	0.74	1.13
	66VO	0.31	0.41
Complement component C8 gamma chain	*FO*	0.54	0.59
	66VO	0.30	0.32
Complement factor B	*FO*	0.62	0.68
	66VO	0.58	0.47
Complement factor I	*FO*	0.37	0.46
	66VO	0.59	0.51
Conserved oligomeric Golgi complex component 7	*FO*	1.04	1.28
	66VO	1.09	1.18
Copper transport protein ATOX1	*FO*	0.87	0.81
	66VO	0.51	0.56
Cordon-bleu 1	*FO*	1.58	1.22
	66VO	0.54	0.72
COX16-like protein C14orf112 homolog, mitochondrial	*FO*	0.84	0.76
	66VO	0.90	1.10
Cryptochrome 1a	*FO*	0.62	0.69
	66VO	0.53	0.47
C-terminal-binding protein 2	*FO*	0.57	0.52
	66VO	0.40	0.46
Cullin-1	*FO*	0.72	0.71
	66VO	0.75	0.63
Cullin-3	*FO*	0.54	1.36
	66VO	0.85	1.23
C-X-C chemokine receptor type 7	*FO*	0.51	0.45
	66VO	0.24	0.29
C-X-C motif chemokine 14	*FO*	0.45	0.27
	66VO	0.40	0.41
CXXC-type zinc finger protein 6	*FO*	0.66	0.66
	66VO	0.46	0.56
Cyclin d-type binding-protein 1	*FO*	0.79	0.81
	66VO	0.85	0.64
Cyclin G2	*FO*	0.71	0.60
	66VO	0.53	0.64
Cysteine protease ATG4D	*FO*	1.07	0.87
	66VO	0.45	0.40
Cysteine-rich PDZ-binding protein	*FO*	0.68	0.69
	66VO	0.60	0.63
Cysteine-rich protein 2	*FO*	0.75	0.52
	66VO	1.28	0.70
Cystinosin	*FO*	0.60	0.75
	66VO	0.56	0.56
Cytochrome b ascorbate-dependent protein 3	*FO*	0.35	0.47
	66VO	0.36	0.34
Cytochrome P450 24A1, mitochondrial	*FO*	0.63	0.45
	66VO	0.56	0.31
Cytochrome P450 2J2	*FO*	0.52	0.54
	66VO	0.22	0.27
Cytochrome P450 2K4	*FO*	0.54	0.48
	66VO	0.29	0.32
Cytochrome P450 4A10	*FO*	0.67	0.71
	66VO	1.29	0.47
Cytoplasmic dynein 1 heavy chain 1	*FO*	0.73	0.56
	66VO	0.40	0.37
Cytoplasmic dynein 1 intermediate chain 2	*FO*	0.23	0.24
	66VO	0.81	0.91
Cytosolic alanine aminotransferase 2	*FO*	0.42	0.51

	66VO	0.31	0.34
Cytosolic sulfotransferase 1	*FO*	0.23	0.28
	66VO	0.32	0.40
Dachshund homolog 1	*FO*	0.60	0.66
	66VO	0.51	0.46
DCN1-like protein 5	*FO*	0.42	0.44
	66VO	0.35	0.42
Deleted in azoospermia-like	*FO*	0.53	0.53
	66VO	0.36	0.42
Delta-like protein 4	*FO*	0.71	0.59
	66VO	0.43	0.50
Deoxyribonuclease gamma	*FO*	0.66	0.40
	66VO	0.33	0.35
Dermal papilla-derived protein 6 homolog	*FO*	0.52	0.84
	66VO	0.32	0.45
Diablo homolog, mitochondrial	*FO*	0.86	0.84
	66VO	0.59	0.63
Diamine acetyltransferase 1	*FO*	0.55	0.51
	66VO	0.27	0.39
Differentially expressed in FDCP 6 homolog	*FO*	0.90	1.17
	66VO	0.81	0.57
Dipeptidyl aminopeptidase-like protein 6	*FO*	0.67	1.05
	66VO	0.29	0.18
Dipeptidyl peptidase 9	*FO*	0.60	0.56
	66VO	0.32	0.32
Dipeptidyl-peptidase 1	*FO*	0.60	0.76
	66VO	0.48	0.49
Dipeptidylpeptidase 3	*FO*	0.78	0.82
	66VO	0.65	0.51
Disabled homolog 2	*FO*	0.39	0.40
	66VO	0.26	0.31
DLA class I histocompatibility antigen, A9/A9 alpha chain	*FO*	0.43	0.62
	66VO	0.25	0.54
DNA polymerase beta	*FO*	0.80	0.75
	66VO	0.67	0.62
DNA polymerase subunit delta-4	*FO*	0.76	0.88
	66VO	0.58	0.56
DNA topoisomerase 1	*FO*	0.62	0.55
	66VO	0.39	0.48
DNA-(apurinic or apyrimidinic site) lyase 2	*FO*	0.77	0.59
	66VO	0.75	0.80
DNA-damage-inducible transcript 4 protein	*FO*	1.65	1.31
	66VO	0.64	0.24
DnaJ homolog subfamily A member 4	*FO*	0.71	0.57
	66VO	0.72	0.54
DnaJ homolog subfamily B member 12	*FO*	0.70	0.73
	66VO	0.73	0.71
DnaJ homolog subfamily B member 14	*FO*	0.66	0.71
	66VO	0.65	0.58
DnaJ homolog subfamily C member 7	*FO*	0.75	0.66
	66VO	0.52	0.48
Docking protein 7	*FO*	0.46	0.54
	66VO	0.32	0.35
Dolichol-phosphate mannosyltransferase subunit 3	*FO*	0.78	0.86
	66VO	0.74	0.73
Dolichyl-diphosphooligosaccharide--protein glycosyltransferase subunit S[illegible]	*FO*	0.80	0.79
	66VO	0.71	0.80
Dual specificity mitogen-activated protein kinase kinase 4	*FO*	0.71	0.78
	66VO	0.54	0.69

Dual specificity protein kinase CLK1	*FO*	0.67	0.66
	66VO	0.45	0.54
Dual specificity protein kinase CLK4	*FO*	0.66	0.66
	66VO	0.55	0.67
Dual specificity protein phosphatase 5	*FO*	0.93	0.89
	66VO	0.43	0.80
Dynactin subunit 6	*FO*	0.67	0.64
	66VO	0.52	0.55
Dynein light chain roadblock-type 2	*FO*	0.76	0.67
	66VO	0.43	0.66
Dynein light chain Tctex-type 1	*FO*	0.69	0.62
	66VO	0.56	0.66
E2-induced gene 5 protein homolog	*FO*	0.77	0.97
	66VO	0.54	0.71
E3 SUMO-protein ligase CBX4	*FO*	0.67	0.83
	66VO	0.40	0.40
E3 ubiquitin-protein ligase CHFR	*FO*	0.69	0.71
	66VO	0.68	0.61
E3 ubiquitin-protein ligase MARCH3	*FO*	0.80	0.72
	66VO	0.64	0.59
E3 ubiquitin-protein ligase NEDD4	*FO*	0.51	0.49
	66VO	0.28	0.37
E3 ubiquitin-protein ligase NEDD4-like protein	*FO*	0.49	0.51
	66VO	0.25	0.36
E3 ubiquitin-protein ligase SMURF1	*FO*	0.62	0.60
	66VO	0.71	0.62
E3 ubiquitin-protein ligase UBR2	*FO*	0.59	0.51
	66VO	0.42	0.41
E3 ubiquitin-protein ligase UBR3	*FO*	0.47	0.53
	66VO	0.38	0.52
E3 ubiquitin-protein ligase UBR5	*FO*	0.92	0.99
	66VO	1.38	1.32
EF-hand calcium-binding domain-containing protein 2	*FO*	0.50	0.39
	66VO	0.22	0.39
EF-hand domain-containing family member A1	*FO*	0.69	0.62
	66VO	0.52	0.61
EGF-like domain-containing protein 7	*FO*	0.52	0.47
	66VO	0.65	0.51
Elastase-3B	*FO*	0.57	0.45
	66VO	0.49	0.43
ELAV-like protein 2	*FO*	0.28	0.26
	66VO	0.22	0.48
Ellis van creveld	*FO*	0.45	0.44
	66VO	0.52	0.51
ELMO domain-containing protein 2	*FO*	0.65	0.71
	66VO	0.43	0.47
Endonuclease/exonuclease/phosphatase family domain-containing protei	*FO*	0.70	0.70
	66VO	0.48	0.53
Endothelial lipase	*FO*	0.65	0.58
	66VO	0.15	0.18
Endothelial zinc finger protein induced by tumor necrosis factor alpha	*FO*	0.64	0.65
	66VO	0.31	0.36
Endothelin-converting enzyme 1	*FO*	0.65	0.59
	66VO	0.67	0.60
Enhancer of polycomb 1 isoform 1	*FO*	0.67	0.65
	66VO	0.58	0.63
Enhancer of polycomb homolog 1	*FO*	0.69	0.66
	66VO	0.57	0.72
Enoyl coenzyme a hydratase domain containing 3	*FO*	0.50	0.33

	66VO	0.30	0.32
Ependymin-1	*FO*	0.50	0.59
	66VO	0.26	0.42
Ephrin-A5b	*FO*	0.56	0.57
	66VO	0.50	0.70
Epiplakin	*FO*	0.46	0.52
	66VO	0.27	0.55
Epoxide hydrolase 1	*FO*	0.73	0.71
	66VO	0.42	0.59
Ethanolamine kinase 2	*FO*	0.52	0.36
	66VO	0.60	0.69
Eukaryotic initiation factor 4A-II	*FO*	0.60	0.78
	66VO	0.70	0.61
Eukaryotic translation initiation factor subunit 10	*FO*	0.75	0.70
	66VO	0.52	0.66
Exocyst complex component 1	*FO*	0.89	0.89
	66VO	0.64	0.71
Exocyst complex component 2	*FO*	0.68	0.69
	66VO	0.64	0.68
Exocyst complex component 4	*FO*	0.46	0.74
	66VO	0.44	0.48
Exocyst complex component 7	*FO*	0.79	0.91
	66VO	0.70	0.82
Exosome complex exonuclease MTR3	*FO*	0.73	0.70
	66VO	0.63	0.54
Exostosin-2	*FO*	0.66	0.74
	66VO	0.72	0.74
Extracellular matrix protein 2	*FO*	0.72	0.71
	66VO	0.80	0.75
Extracellular peptidase inhibitor	*FO*	0.57	0.53
	66VO	0.48	0.44
Fanconi anemia group F protein	*FO*	0.76	0.65
	66VO	0.55	0.50
Fatty acid-binding protein, brain	*FO*	0.54	0.48
	66VO	0.65	0.43
F-box only protein 42	*FO*	0.86	0.92
	66VO	0.68	0.68
Ferritin, lower subunit	*FO*	0.58	0.68
	66VO	0.38	0.52
FGFR1 oncogene partner 2 homolog	*FO*	0.45	0.36
	66VO	0.46	0.45
Fibrinogen beta chain	*FO*	0.68	0.63
	66VO	0.55	0.57
Fibroblast growth factor receptor homolog 1	*FO*	0.95	1.05
	66VO	0.43	0.78
Fibronectin	*FO*	0.71	0.68
	66VO	0.53	0.53
FK506-binding protein 1B	*FO*	0.51	0.53
	66VO	0.53	0.59
FKBP12-rapamycin complex-associated protein	*FO*	0.82	0.70
	66VO	0.68	0.56
Flocculin-like partial	*FO*	0.52	0.37
	66VO	0.44	0.55
Follistatin Mrna	*FO*	0.72	0.55
	66VO	1.40	0.43
Forkhead box protein O3	*FO*	0.81	0.75
	66VO	0.52	0.57
Four and a half LIM domains protein 2	*FO*	0.41	0.62
	66VO	0.53	0.47

Four and a half LIM domains protein 3	*FO*	0.93	0.89
	66VO	0.29	0.56
Fructose-bisphosphate aldolase A	*FO*	0.54	0.34
	66VO	0.23	0.29
Fumarylacetoacetate hydrolase domain-containing protein 2	*FO*	0.71	0.54
	66VO	0.32	0.49
Fumarylacetoacetate hydrolase domain-containing protein 2B	*FO*	0.64	0.62
	66VO	0.46	0.62
FUS-interacting serine-arginine-rich protein 1	*FO*	0.59	0.59
	66VO	0.62	0.69
FXYD domain-containing ion transport regulator 6	*FO*	0.61	0.62
	66VO	0.37	0.69
FYVE finger-containing phosphoinositide kinase	*FO*	0.69	0.73
	66VO	0.56	0.61
G protein pathway suppressor 2	*FO*	0.56	0.50
	66VO	0.42	0.47
G7c protein	*FO*	0.61	0.48
	66VO	0.17	0.26
Galectin 8	*FO*	0.47	0.46
	66VO	0.32	0.31
Gametogenetin-binding protein 2	*FO*	0.60	0.60
	66VO	0.54	0.56
Gamma-aminobutyric acid receptor-associated protein	*FO*	0.82	0.77
	66VO	0.56	0.59
Gamma-aminobutyric acid receptor-associated protein-like 2	*FO*	0.62	0.63
	66VO	0.45	0.44
Gamma-secretase subunit Aph-1b	*FO*	0.73	0.88
	66VO	0.56	0.64
Gamma-secretase subunit Psenen	*FO*	0.65	0.74
	66VO	0.53	0.60
Gap junction Cx32.2 protein	*FO*	0.52	0.52
	66VO	0.29	0.34
Gastrula zinc finger protein XlCGF57.1	*FO*	0.68	0.74
	66VO	0.55	0.89
GC-rich sequence DNA-binding factor homolog	*FO*	0.75	0.72
	66VO	0.60	0.75
GDH/6PGL endoplasmic bifunctional protein	*FO*	0.71	0.67
	66VO	0.66	0.77
GDNF-inducible zinc finger protein 1	*FO*	0.72	0.70
	66VO	0.74	0.72
GDP-fucose protein O-fucosyltransferase 1	*FO*	0.73	0.74
	66VO	0.56	0.55
Gem-associated protein 5	*FO*	0.79	0.77
	66VO	0.34	0.47
Gla-rich protein	*FO*	2.44	5.31
	66VO	0.27	0.43
Glucagon receptor	*FO*	0.72	0.65
	66VO	0.35	0.78
Glutaminase kidney isoform, mitochondrial	*FO*	0.62	0.71
	66VO	0.37	0.44
Glutamine-rich protein 1	*FO*	0.78	0.85
	66VO	0.55	0.71
Glutaryl-CoA dehydrogenase, mitochondrial	*FO*	0.66	0.80
	66VO	0.33	0.54
Glutathione S-transferase theta-1	*FO*	0.62	0.72
	66VO	0.40	0.56
Glycerol kinase 2	*FO*	0.60	0.56
	66VO	0.57	0.55
Glycerol testis specific 1	*FO*	0.77	0.60

	66VO	0.26	0.40
Glycogen phosphorylase, muscle form	*FO*	0.70	0.76
	66VO	0.32	0.60
Glycolipid transfer protein	*FO*	0.77	0.80
	66VO	0.79	0.81
Glycoprotein endo-alpha-1,2-mannosidase	*FO*	0.60	0.63
	66VO	0.35	0.48
Glycosyltransferase 8 domain-containing protein 3	*FO*	0.57	0.52
	66VO	0.35	0.42
Glyoxalase domain-containing protein 4	*FO*	0.82	0.65
	66VO	0.53	0.49
Golgi SNAP receptor complex member 2	*FO*	0.74	0.82
	66VO	0.62	0.60
Golgin subfamily A member 4	*FO*	0.66	0.79
	66VO	0.49	0.55
Golgin subfamily A member 5	*FO*	0.71	0.74
	66VO	0.43	0.49
Golgin subfamily A member 7	*FO*	0.51	0.48
	66VO	0.35	0.45
GPI mannosyltransferase 1	*FO*	0.71	0.71
	66VO	0.72	0.70
Granzyme B(G,H)	*FO*	0.85	1.08
	66VO	1.21	1.94
Green-sensitive opsin	*FO*	0.71	0.62
	66VO	0.79	0.79
Growth hormone receptor type I	*FO*	0.36	0.36
	66VO	0.28	0.35
Growth hormone receptor type II	*FO*	0.76	0.53
	66VO	0.33	0.37
Growth inhibition and differentiation-related protein 88 homolog	*FO*	0.71	0.66
	66VO	0.55	0.58
GTP cyclohydrolase 1 feedback regulatory protein	*FO*	0.54	0.37
	66VO	0.37	0.47
GTPase activating protein and vps9 domains 1	*FO*	0.37	0.39
	66VO	0.29	0.46
GTPase IMAP family member 5	*FO*	0.74	0.48
	66VO	0.35	0.79
GTPase-activating Rap/Ran-GAP domain-like 1	*FO*	0.42	0.64
	66VO	0.44	0.38
GTP-binding protein 2	*FO*	0.77	0.62
	66VO	0.54	0.55
Guanine nucleotide-binding protein alpha-14 subunit	*FO*	0.75	0.71
	66VO	0.27	0.38
Guanine nucleotide-binding protein G(I)/G(S)/G(O) subunit gamma-2	*FO*	0.62	0.69
	66VO	0.57	0.60
Haloacid dehalogenase-like hydrolase domain-containing protein 2	*FO*	0.89	0.98
	66VO	0.65	0.76
Haloacid dehalogenase-like hydrolase domain-containing protein 3	*FO*	0.82	0.76
	66VO	0.67	0.81
Harmonin	*FO*	0.55	0.64
	66VO	0.29	0.51
Hat family dimerisation domain containing expressed	*FO*	0.65	0.69
	66VO	0.83	1.13
Hcls1-associated protein x-1	*FO*	0.67	0.61
	66VO	0.56	0.50
HD domain-containing protein 2	*FO*	0.72	0.81
	66VO	0.60	0.54
HD domain-containing protein 3	*FO*	0.56	0.54
	66VO	0.36	0.42

Heat shock 70 kDa protein 4L	*FO*	0.63	0.61
	66VO	0.49	0.54
Heat shock factor-binding protein 1	*FO*	0.51	0.64
	66VO	0.40	0.45
Heat shock protein 67B2	*FO*	0.45	0.40
	66VO	0.31	0.37
Hemicentin 1	*FO*	0.60	0.61
	66VO	0.46	0.50
Hemoglobin subunit alpha-A	*FO*	0.38	0.35
	66VO	0.33	0.40
Hemoglobin subunit alpha-B	*FO*	0.62	0.69
	66VO	0.31	0.47
Heterogeneous nuclear ribonucleoprotein H2	*FO*	0.71	0.67
	66VO	0.51	0.52
Heterogeneous nuclear ribonucleoprotein Q	*FO*	0.75	0.68
	66VO	0.56	0.66
High choriolytic enzyme 2	*FO*	0.13	0.12
	66VO	0.23	0.29
High mobility group protein B1	*FO*	0.91	0.85
	66VO	0.90	0.85
Histamine N-methyltransferase	*FO*	0.54	0.59
	66VO	0.36	0.47
Histone deacetylase complex subunit SAP130	*FO*	0.72	0.71
	66VO	0.68	0.74
Histone demethylase JARID1B	*FO*	0.71	0.87
	66VO	0.51	0.58
Histone H1	*FO*	0.64	0.48
	66VO	0.56	0.60
Histone H2A type 2-B	*FO*	0.47	0.44
	66VO	0.38	0.45
Histone H2A.x	*FO*	0.60	0.65
	66VO	0.42	0.51
Histone H3.3	*FO*	0.63	0.65
	66VO	0.61	0.59
Histone H3.3b	*FO*	0.75	0.84
	66VO	0.38	0.46
Histone-binding protein RBBP4	*FO*	1.22	1.26
	66VO	1.50	1.37
HIV Tat-specific factor 1 homolog	*FO*	0.59	0.51
	66VO	0.51	0.69
Homeodomain-interacting protein kinase 1	*FO*	0.69	0.79
	66VO	0.41	0.50
Hsp90 co-chaperone Cdc37	*FO*	0.96	0.93
	66VO	1.43	1.23
Hydroxyacyl-coenzyme A dehydrogenase, mitochondrial	*FO*	0.80	0.66
	66VO	0.63	0.64
IgGFc-binding protein	*FO*	0.64	0.58
	66VO	0.42	0.59
Immature colon carcinoma transcript 1 protein	*FO*	0.68	0.70
	66VO	0.65	0.69
Immediate early response 3-interacting protein 1	*FO*	0.73	0.76
	66VO	0.66	0.76
Immediate early response 5-like	*FO*	0.56	0.56
	66VO	0.38	0.53
Importin-7	*FO*	0.71	0.72
	66VO	0.83	0.75
Inhibitor of Bruton tyrosine kinase	*FO*	0.75	0.66
	66VO	0.76	0.19
Inhibitor of DNA binding 4	*FO*	0.52	0.51

	66VO	0.43	0.48
Inhibitor of growth protein 4	*FO*	0.71	0.71
	66VO	0.46	0.69
Inhibitor of growth protein 5	*FO*	0.56	0.55
	66VO	0.58	0.50
Inositol hexakisphosphate kinase 2	*FO*	0.47	0.47
	66VO	0.30	0.33
Insulin-like growth factor II	*FO*	0.44	0.36
	66VO	0.30	0.37
Insulin-like growth factor-binding protein 4	*FO*	0.47	0.48
	66VO	0.22	0.42
Integral membrane protein 2B	*FO*	0.66	0.64
	66VO	0.59	0.61
Integral membrane protein GPR175	*FO*	0.66	0.67
	66VO	0.41	0.49
Integrin alpha-4	*FO*	0.46	0.65
	66VO	0.33	0.45
Inter-alpha-trypsin inhibitor heavy chain H2	*FO*	0.50	0.35
	66VO	0.26	0.34
Interferon-related developmental regulator 1	*FO*	0.85	0.82
	66VO	0.81	0.67
Interleukin-1 receptor type II	*FO*	0.80	0.63
	66VO	0.58	0.47
Intraflagellar transport 20 homolog	*FO*	0.91	0.84
	66VO	1.46	0.89
Intraflagellar transport 80 homolog	*FO*	0.88	0.83
	66VO	0.70	0.74
Iron-sulfur cluster assembly 2 homolog, mitochondrial	*FO*	0.55	0.54
	66VO	0.47	0.44
Iron-sulfur cluster assembly enzyme ISCU, mitochondrial	*FO*	0.75	0.72
	66VO	0.45	0.53
Isocitrate dehydrogenase [NADP], mitochondrial	*FO*	0.58	0.58
	66VO	0.29	0.40
JmjC domain-containing protein 3	*FO*	0.63	0.62
	66VO	0.51	0.62
Junction plakoglobin	*FO*	0.78	0.82
	66VO	0.57	0.58
Junctional adhesion molecule A	*FO*	0.62	0.77
	66VO	0.35	0.52
Junctional adhesion molecule C	*FO*	0.82	0.49
	66VO	0.40	0.31
Kelch domain-containing protein 1	*FO*	0.57	0.46
	66VO	0.38	0.50
Kelch-like protein 12	*FO*	0.66	0.61
	66VO	0.40	0.54
Keratin, type I cytoskeletal 18	*FO*	1.12	0.92
	66VO	1.42	0.75
KN motif and ankyrin repeat domain-containing protein 1	*FO*	0.70	0.65
	66VO	0.56	0.60
Kruppel-like factor 2a	*FO*	0.45	0.64
	66VO	0.28	0.60
Kv channel-interacting protein 1	*FO*	0.61	0.69
	66VO	0.24	0.46
L-2-hydroxyglutarate dehydrogenase, mitochondrial	*FO*	0.93	0.70
	66VO	0.46	0.56
Lactoylglutathione lyase	*FO*	0.84	0.81
	66VO	0.68	0.77
LAG1 longevity assurance homolog 2	*FO*	0.35	0.50
	66VO	0.32	0.37

Laminin domain-containing 2	*FO*	0.50	0.53
	66VO	0.43	0.73
Latent-transforming growth factor beta-binding protein 4	*FO*	0.60	0.53
	66VO	0.26	0.55
Leucine-rich repeat-containing protein 42	*FO*	0.66	0.69
	66VO	0.52	0.68
Leucine-rich repeat-containing protein 45	*FO*	1.00	0.80
	66VO	0.57	0.55
Leucine-rich repeat-containing protein 57	*FO*	0.76	0.75
	66VO	0.61	0.53
Leucine-rich repeat-containing protein 58	*FO*	0.52	0.62
	66VO	0.43	0.38
Leucine-rich repeat-containing protein 8D	*FO*	0.40	0.52
	66VO	0.51	0.56
Leucine-zipper-like transcriptional regulator 1	*FO*	0.73	0.70
	66VO	0.58	0.61
Leukocyte immune-type receptor	*FO*	0.94	0.85
	66VO	0.47	0.74
LIM domain-binding protein 1	*FO*	0.76	0.72
	66VO	0.58	0.70
LINE-1 reverse transcriptase homolog	*FO*	0.70	0.61
	66VO	0.69	0.57
Lipoma HMGIC fusion partner-like 3 protein	*FO*	0.55	0.52
	66VO	0.42	0.39
Liver X receptor alpha	*FO*	0.69	0.82
	66VO	0.46	0.57
LMBR1 domain-containing protein 1	*FO*	0.54	0.55
	66VO	0.43	0.45
Long-chain specific acyl-CoA dehydrogenase, mitochondrial	*FO*	0.53	0.58
	66VO	0.37	0.48
Long-chain-fatty-acid--CoA ligase 4	*FO*	0.79	0.88
	66VO	0.46	0.50
Loricrin	*FO*	0.65	0.62
	66VO	0.59	0.49
LYR motif-containing protein 1	*FO*	0.91	0.92
	66VO	1.53	1.28
Lysosomal alpha-mannosidase	*FO*	0.50	0.64
	66VO	0.22	0.31
Lysosomal alpha-n-acetyl glucosaminidase	*FO*	0.76	0.82
	66VO	0.49	0.59
Lysosomal-associated transmembrane protein 4A	*FO*	0.49	0.48
	66VO	0.31	0.36
Lysosome membrane protein 2	*FO*	0.75	0.72
	66VO	0.72	0.78
Macrophage expressed 1	*FO*	0.80	0.86
	66VO	0.72	0.57
Major facilitator superfamily domain-containing protein 1	*FO*	0.46	0.50
	66VO	0.35	0.40
Major facilitator superfamily domain-containing protein 5	*FO*	0.75	0.84
	66VO	0.71	0.75
Major facilitator superfamily domain-containing protein 8	*FO*	0.42	0.52
	66VO	0.30	0.34
Makorin-1	*FO*	0.64	0.62
	66VO	0.45	0.46
Malate dehydrogenase, cytoplasmic	*FO*	1.23	0.98
	66VO	0.67	0.74
Malate dehydrogenase, mitochondrial	*FO*	0.89	0.76
	66VO	0.76	0.74
Mammalian ependymin-related protein 1	*FO*	0.72	0.56

	66VO	0.29	0.45
Mannose-P-dolichol utilization defect 1 protein	*FO*	0.44	0.48
	66VO	0.79	0.70
MARVEL domain-containing protein 2	*FO*	0.53	0.67
	66VO	0.29	0.55
Matrin 3-like	*FO*	0.72	0.73
	66VO	0.46	0.64
Matrin-3	*FO*	0.74	0.83
	66VO	0.74	0.75
Matrix metalloproteinase-28	*FO*	0.76	0.69
	66VO	0.64	0.73
McKusick-Kaufman/Bardet-Biedl syndromes putative chaperonin	*FO*	0.79	0.79
	66VO	0.74	0.76
Mediator of RNA polymerase II transcription subunit 1	*FO*	0.51	0.79
	66VO	0.25	0.55
Mediator of RNA polymerase II transcription subunit 6	*FO*	0.67	0.68
	66VO	0.58	0.54
Medium-chain specific acyl-CoA dehydrogenase, mitochondrial	*FO*	0.76	0.74
	66VO	0.56	0.61
Metabotropic glutamate receptor 7	*FO*	0.87	0.56
	66VO	0.53	0.55
Metallophosphoesterase domain-containing protein 1	*FO*	0.42	0.45
	66VO	0.45	0.51
Methionine-R-sulfoxide reductase	*FO*	0.67	0.74
	66VO	0.55	0.62
Methyl-CpG-binding domain protein 4	*FO*	0.80	0.71
	66VO	0.41	0.54
Methylmalonate-semialdehyde dehydrogenase [acylating], mitochondrial	*FO*	0.85	0.89
	66VO	0.38	0.81
Methylosome subunit pICln	*FO*	0.79	0.71
	66VO	0.68	0.65
Microspherule protein 1	*FO*	0.75	0.69
	66VO	0.47	0.47
Microtubule-associated proteins 1A/1B light chain 3B	*FO*	0.72	0.69
	66VO	0.34	0.35
Microtubule-associated proteins 1A/1B light chain 3C	*FO*	0.45	0.25
	66VO	0.51	0.49
Mid1-interacting protein 1	*FO*	0.75	0.72
	66VO	0.41	0.50
MIT domain-containing protein 1	*FO*	0.68	0.68
	66VO	0.57	0.72
Mitochondrial fission 1 protein	*FO*	0.65	0.75
	66VO	0.52	0.50
Mitochondrial tumor suppressor 1 homolog	*FO*	0.67	0.61
	66VO	0.34	0.57
Mitogen-activated protein kinase 15	*FO*	0.76	1.09
	66VO	0.26	0.67
Mitogen-activated protein kinase kinase kinase kinase 5	*FO*	0.75	0.92
	66VO	0.75	0.85
Mitotic spindle assembly checkpoint protein MAD2B	*FO*	0.69	0.60
	66VO	0.47	0.48
MLN64 N-terminal domain homolog	*FO*	0.66	0.76
	66VO	0.42	0.64
MORC family CW-type zinc finger protein 4	*FO*	0.72	0.69
	66VO	0.57	0.53
Mortality factor 4-like protein 1	*FO*	0.60	0.53
	66VO	0.30	0.42
Mothers against decapentaplegic homolog 5	*FO*	0.64	0.61
	66VO	0.72	0.61

Mps one binder kinase activator-like 2A	*FO*	1.17	1.25
	66VO	1.44	1.32
Mucin-2	*FO*	0.59	0.61
	66VO	0.38	0.43
Mu-crystallin	*FO*	0.63	0.67
	66VO	0.36	0.54
Multidrug resistance protein 3	*FO*	0.57	0.70
	66VO	0.27	0.49
MUM1-like protein 1	*FO*	0.74	0.61
	66VO	0.44	0.46
Myelin P2	*FO*	0.20	0.26
	66VO	0.23	0.71
Myeloma overexpressed 2	*FO*	0.69	0.69
	66VO	0.70	0.75
Myosin-binding protein H	*FO*	0.65	0.55
	66VO	0.56	0.57
N-acetylserotonin O-methyltransferase-like protein	*FO*	0.37	0.43
	66VO	0.25	0.43
N-acetyltransferase ESCO1	*FO*	0.65	0.68
	66VO	0.67	0.59
NACHT, LRR and PYD domains-containing protein 12	*FO*	0.36	0.32
	66VO	0.22	0.35
NACHT, LRR and PYD domains-containing protein 3	*FO*	0.57	0.54
	66VO	0.36	0.40
N-acylglucosamine 2-epimerase	*FO*	0.64	0.53
	66VO	0.53	0.39
NADH dehydrogenase [ubiquinone] 1 alpha subcomplex subunit 4-like 2	*FO*	0.53	0.42
	66VO	0.53	0.55
NADH dehydrogenase [ubiquinone] 1 beta subcomplex subunit 3	*FO*	0.64	0.63
	66VO	0.25	0.41
NADH-ubiquinone oxidoreductase chain 1	*FO*	0.80	0.91
	66VO	0.64	0.76
NADH-ubiquinone oxidoreductase chain 2	*FO*	0.61	0.59
	66VO	0.46	0.61
NADPH--cytochrome P450 reductase	*FO*	0.95	0.75
	66VO	0.61	0.61
Nattectin	*FO*	0.62	0.66
	66VO	0.67	0.39
NEDD4-like E3 ubiquitin-protein ligase WWP1	*FO*	0.43	0.46
	66VO	0.25	0.34
NEDD8-activating enzyme E1 catalytic subunit	*FO*	0.78	0.76
	66VO	0.57	0.71
Netrin receptor UNC5C	*FO*	2.19	2.42
	66VO	0.50	0.42
Neural cell adhesion molecule 1, 140 kDa isoform	*FO*	0.68	0.68
	66VO	0.44	0.68
Neurogranin	*FO*	0.80	1.11
	66VO	0.43	0.85
Neuronal cell adhesion molecule	*FO*	0.51	0.38
	66VO	0.35	0.44
Neuronal-specific septin-3	*FO*	0.72	0.68
	66VO	0.47	0.62
NF-kappa-B inhibitor-like protein 1	*FO*	0.72	0.69
	66VO	0.54	0.65
NFU1 iron-sulfur cluster scaffold homolog	*FO*	0.65	0.62
	66VO	0.51	0.53
Nischarin	*FO*	0.73	0.70
	66VO	0.42	0.27
Non-receptor tyrosine-protein kinase TYK2	*FO*	0.73	0.86

	66VO	0.62	0.61
Non-specific lipid-transfer protein	*FO*	0.48	0.33
	66VO	0.30	0.27
Normal mucosa of esophagus-specific gene 1 protein	*FO*	0.75	0.88
	66VO	0.49	0.65
Novel protein vertebrate desmuslin	*FO*	0.59	0.61
	66VO	0.41	0.70
Novel protein vertebrate member ras oncogene family	*FO*	0.52	0.64
	66VO	0.34	0.47
N-sulphoglucosamine sulphohydrolase	*FO*	0.48	0.47
	66VO	0.25	0.32
Nuclear factor 7, brain	*FO*	0.74	0.78
	66VO	0.38	0.57
Nuclear factor erythroid 2-related factor 2	*FO*	0.66	0.61
	66VO	0.57	0.52
Nuclear receptor coactivator 7	*FO*	0.32	0.42
	66VO	0.43	0.41
Nuclear receptor subfamily 2 group C member 1-B	*FO*	0.76	0.79
	66VO	0.48	0.70
Nucleobindin-2	*FO*	0.64	0.54
	66VO	0.42	0.34
Nucleolar GTP-binding protein 1	*FO*	0.71	0.80
	66VO	0.68	0.80
Nucleolar RNA helicase 2	*FO*	0.64	0.62
	66VO	0.47	0.62
Nucleolin	*FO*	0.54	0.43
	66VO	0.37	0.37
Nucleotide-binding protein 2	*FO*	0.51	0.69
	66VO	0.46	0.41
Octapeptide-repeat protein T2	*FO*	0.68	0.62
	66VO	0.44	0.49
Optineurin	*FO*	1.09	1.30
	66VO	1.26	1.12
Organic solute transporter alpha	*FO*	0.46	0.56
	66VO	0.43	0.45
ORM1-like protein 1	*FO*	0.60	0.44
	66VO	0.27	0.31
Ornithine decarboxylase antizyme 1	*FO*	0.78	0.86
	66VO	0.75	0.65
Ornithine decarboxylase antizyme 2	*FO*	0.72	0.72
	66VO	0.69	0.64
Osteoclast-stimulating factor 1	*FO*	0.99	1.09
	66VO	1.39	1.20
Oxysterol-binding protein-related protein 1	*FO*	0.64	0.61
	66VO	0.74	0.54
P2X purinoceptor 1	*FO*	0.95	0.68
	66VO	0.62	0.68
P2Y purinoceptor 5	*FO*	0.78	0.66
	66VO	0.63	0.53
P3 protein	*FO*	0.91	0.98
	66VO	0.87	0.88
p53 apoptosis effector related to PMP-22	*FO*	1.95	0.91
	66VO	0.82	0.89
Palmitoyltransferase ZDHHC7	*FO*	0.72	0.58
	66VO	0.29	0.37
Pancreatic carboxypeptidase a1	*FO*	0.34	0.43
	66VO	0.18	0.24
Parafibromin	*FO*	0.55	0.56
	66VO	0.40	0.38

Peptide methionine sulfoxide reductase	*FO*	0.55	0.65
	66VO	0.37	0.65
Peptide methionine sulfoxide reductase msrB	*FO*	0.70	0.77
	66VO	0.25	0.49
Peptide-N(4)-(N-acetyl-beta-glucosaminyl) asparagine amidase	*FO*	0.79	0.92
	66VO	0.67	0.69
Perforin	*FO*	0.93	1.18
	66VO	0.50	0.72
Pericentriolar material 1 protein	*FO*	0.61	0.57
	66VO	0.55	0.61
Period circadian protein homolog 2	*FO*	0.88	0.71
	66VO	0.39	0.39
Peripheral myelin protein 22	*FO*	0.51	0.54
	66VO	0.38	0.49
Peroxiredoxin-6	*FO*	0.78	0.78
	66VO	0.54	0.51
Peroxisomal carnitine O-octanoyltransferase	*FO*	0.58	0.54
	66VO	0.34	0.50
Peroxisomal Lon protease homolog 2	*FO*	0.71	0.54
	66VO	0.53	0.55
Peroxisomal membrane protein 2	*FO*	0.70	0.64
	66VO	0.62	0.74
Peroxisomal multifunctional enzyme type 2	*FO*	0.85	0.75
	66VO	0.51	0.64
Pescadillo	*FO*	0.70	0.63
	66VO	0.54	0.41
PEST proteolytic signal-containing nuclear protein	*FO*	0.73	0.76
	66VO	0.78	0.83
Phenylalanine-4-hydroxylase	*FO*	0.67	0.58
	66VO	0.53	0.48
PHI/VIP	*FO*	0.39	0.38
	66VO	0.27	0.36
Phosphatase and actin regulator 1	*FO*	0.44	0.42
	66VO	0.27	0.39
Phosphatase and actin regulator 3	*FO*	0.63	0.51
	66VO	0.30	0.41
Phosphatase and actin regulator 4	*FO*	0.54	0.56
	66VO	0.45	0.55
Phosphatase PSR1	*FO*	0.69	0.72
	66VO	0.46	0.55
Phosphatidylcholine:ceramide cholinephosphotransferase 2	*FO*	0.56	0.44
	66VO	0.49	0.56
Phosphatidylethanolamine N-methyltransferase	*FO*	0.57	0.50
	66VO	0.24	0.35
Phosphatidylinositide phosphatase SAC1-A	*FO*	0.70	0.71
	66VO	0.62	0.73
Phosphatidylinositol 4-kinase alpha	*FO*	0.54	0.65
	66VO	0.32	0.42
Phosphatidylinositol 4-kinase type 2-alpha	*FO*	0.68	0.69
	66VO	0.50	0.50
Phosphatidylinositol N-acetylglucosaminyltransferase subunit H	*FO*	0.67	0.82
	66VO	0.61	0.57
Phosphatidylinositol transfer protein alpha isoform	*FO*	0.61	0.50
	66VO	0.73	0.85
Phosphoglucomutase-like protein 5	*FO*	0.74	0.73
	66VO	0.33	0.65
Phosphoinositide 3-kinase regulatory subunit 4	*FO*	0.58	0.66
	66VO	0.36	0.39
Phospholipase D3	*FO*	0.60	0.63

	66VO	0.49	0.44
Phosphomannomutase 1	*FO*	0.64	0.65
	66VO	0.33	0.39
Phosphoribosyl pyrophosphate synthetase-associated protein 1	*FO*	0.83	0.76
	66VO	0.52	0.75
Phytanoyl-CoA dioxygenase, peroxisomal	*FO*	0.88	0.84
	66VO	0.36	0.42
Pituitary tumor-transforming gene 1 protein-interacting protein	*FO*	0.50	0.57
	66VO	0.72	0.69
Piwi-like protein 1	*FO*	0.46	0.47
	66VO	0.43	0.42
Plakophilin-3	*FO*	0.72	0.70
	66VO	0.58	0.55
Platelet receptor Gi24	*FO*	0.70	0.71
	66VO	0.63	0.54
Pleiotrophic factor-alpha-2	*FO*	0.74	0.59
	66VO	0.28	0.38
Pleiotropic regulator 1	*FO*	1.20	1.35
	66VO	0.82	0.78
Poliovirus receptor-related protein 3	*FO*	0.63	0.51
	66VO	0.39	0.45
Poliovirus receptor-related protein 4	*FO*	1.00	0.92
	66VO	0.67	0.61
Poly [ADP-ribose] polymerase 11	*FO*	1.06	0.81
	66VO	0.49	0.65
Poly [ADP-ribose] polymerase 12	*FO*	0.64	0.70
	66VO	0.50	0.60
Polyadenylate-binding protein-interacting protein 2	*FO*	0.62	0.63
	66VO	0.36	0.46
Polycomb protein SCMH1	*FO*	0.55	0.63
	66VO	0.33	0.49
Polyhomeotic-like protein 1	*FO*	0.68	0.62
	66VO	0.41	0.59
Polypeptide N-acetylgalactosaminyltransferase 14	*FO*	0.37	0.42
	66VO	0.37	0.32
Polypyrimidine tract-binding protein 2	*FO*	0.92	0.67
	66VO	0.69	1.08
POU domain, class 5, transcription factor 1	*FO*	0.44	0.55
	66VO	0.29	0.68
Pre-B-cell leukemia transcription factor 1	*FO*	0.60	0.62
	66VO	0.37	0.55
Prefoldin subunit 3	*FO*	0.73	0.76
	66VO	0.60	0.76
Prefoldin subunit 5	*FO*	1.29	1.39
	66VO	1.57	1.43
Pre-mRNA branch site protein p14	*FO*	0.73	0.82
	66VO	0.71	0.59
Pre-mRNA-processing factor 39	*FO*	0.73	0.82
	66VO	0.49	0.55
Pre-mRNA-splicing factor syf2	*FO*	0.88	0.87
	66VO	0.87	0.69
Pre-mRNA-splicing regulator WTAP	*FO*	0.65	0.65
	66VO	0.64	0.72
Probable arylformamidase	*FO*	0.47	0.57
	66VO	0.40	0.45
Probable Bax inhibitor 1	*FO*	0.62	0.63
	66VO	0.57	0.57
Probable G-protein coupled receptor 157	*FO*	0.72	0.78
	66VO	0.27	0.28

Probable isovaleryl-CoA dehydrogenase	*FO*	0.74	0.81
	66VO	0.48	0.50
Probable N-acetyltransferase camello	*FO*	0.59	0.75
	66VO	0.36	0.45
Probable palmitoyltransferase ZDHHC4	*FO*	0.86	0.69
	66VO	0.31	0.56
Probable polypeptide N-acetylgalactosaminyltransferase 8	*FO*	0.82	0.87
	66VO	0.42	0.74
Probable proline racemase	*FO*	0.60	0.63
	66VO	0.60	0.63
Probable ribosome biogenesis protein RLP24	*FO*	0.60	0.52
	66VO	0.51	0.57
Probable tRNA(His) guanylyltransferase	*FO*	0.74	0.71
	66VO	0.76	0.68
Probable ubiquitin-conjugating enzyme E2 W	*FO*	0.66	0.60
	66VO	0.42	0.42
Programmed cell death protein 10	*FO*	0.56	0.88
	66VO	0.36	0.58
Prolactin regulatory element-binding protein	*FO*	0.73	0.72
	66VO	0.55	0.56
Proline synthetase co-transcribed bacterial homolog protein	*FO*	1.02	0.73
	66VO	0.69	0.62
Prominin-1	*FO*	0.59	0.70
	66VO	0.43	0.52
Prostaglandin E synthase	*FO*	0.75	0.73
	66VO	0.40	0.41
Protease-associated domain-containing protein of 21 kDa	*FO*	0.74	0.65
	66VO	0.32	0.46
Protein ariadne-1 homolog	*FO*	0.76	0.89
	66VO	0.63	0.63
Protein ariadne-2 homolog	*FO*	0.74	0.62
	66VO	0.47	0.60
Protein C20orf11 homolog	*FO*	0.69	0.65
	66VO	0.68	0.62
Protein cappuccino homolog	*FO*	0.79	0.81
	66VO	0.64	0.56
Protein CASP	*FO*	0.79	0.69
	66VO	0.64	0.68
Protein CIAO1	*FO*	0.86	0.84
	66VO	0.83	0.78
Protein deltex-3	*FO*	0.86	0.98
	66VO	0.66	1.51
Protein DJ-1	*FO*	0.75	0.74
	66VO	0.51	0.67
Protein FAM117A	*FO*	0.69	0.65
	66VO	0.80	0.73
Protein FAM135A	*FO*	0.83	0.74
	66VO	0.57	0.81
Protein FAM26E	*FO*	0.65	0.67
	66VO	0.61	0.79
Protein FAM43A	*FO*	0.59	0.55
	66VO	0.47	0.46
Protein FAM49A	*FO*	0.76	0.82
	66VO	0.72	0.86
Protein FAM49B	*FO*	0.71	0.89
	66VO	1.17	1.56
Protein FAM8A1	*FO*	0.81	0.93
	66VO	0.68	1.15
Protein FAM98C	*FO*	0.88	0.87

	66VO	0.57	0.65
Protein GPR89A	*FO*	0.39	0.36
	66VO	0.79	0.63
Protein GTLF3B	*FO*	0.67	0.65
	66VO	0.48	0.53
Protein ITFG3	*FO*	0.76	0.76
	66VO	0.72	0.67
Protein jagunal homolog 1-A	*FO*	0.72	0.61
	66VO	0.50	0.53
Protein lin-37 homolog	*FO*	0.77	0.74
	66VO	0.43	0.47
Protein mab-21-like 2	*FO*	0.58	0.55
	66VO	0.24	0.50
Protein MGR2 homolog	*FO*	0.61	0.53
	66VO	0.38	0.48
Protein NLRC3	*FO*	0.81	0.99
	66VO	0.49	1.12
Protein odd-skipped-related 1	*FO*	0.57	0.45
	66VO	0.45	0.56
Protein PAT1 homolog 1	*FO*	0.65	0.71
	66VO	0.71	0.58
Protein phosphatase 1 regulatory subunit 12A	*FO*	0.74	0.71
	66VO	0.45	0.53
Protein phosphatase 1 regulatory subunit 3C	*FO*	0.25	0.22
	66VO	0.55	0.42
Protein phosphatase 1K, mitochondrial	*FO*	0.96	0.95
	66VO	0.42	0.70
Protein phosphatase regulatory subunit 14a	*FO*	0.47	0.39
	66VO	0.45	0.41
Protein phosphatase regulatory subunit b alpha isoform 2	*FO*	0.55	0.63
	66VO	0.59	0.63
Protein rogdi homolog	*FO*	0.50	0.52
	66VO	0.32	0.27
Protein S100-B	*FO*	0.32	0.32
	66VO	0.27	0.33
Protein SERAC1	*FO*	0.71	0.78
	66VO	0.79	0.83
Protein Tob1	*FO*	0.56	0.47
	66VO	0.35	0.44
Protein transport protein Sec24A	*FO*	0.74	0.74
	66VO	0.75	0.69
Protein unc-84 homolog A	*FO*	0.76	0.71
	66VO	0.47	0.54
Protein YIF1A	*FO*	0.82	0.82
	66VO	0.82	0.78
Protein YIPF1	*FO*	0.51	0.49
	66VO	0.30	0.37
Protein YIPF4	*FO*	0.59	0.64
	66VO	0.50	0.56
Protein yippee-like 5	*FO*	0.70	0.65
	66VO	0.44	0.51
Proteinase-activated receptor 2	*FO*	0.50	0.65
	66VO	0.23	0.34
Protein-tyrosine sulfotransferase 1	*FO*	0.62	0.71
	66VO	0.57	0.73
Proto-oncogene tyrosine-protein kinase FER	*FO*	0.80	0.75
	66VO	0.58	0.60
Proto-oncogene tyrosine-protein kinase receptor ret	*FO*	0.66	0.61
	66VO	0.48	0.53

Pterin-4-alpha-carbinolamine dehydratase	*FO*	0.71	0.66
	66VO	0.43	0.74
Putative deoxyribonuclease TATDN2	*FO*	0.85	0.83
	66VO	0.78	0.86
Putative deoxyribonuclease TATDN3	*FO*	0.66	0.63
	66VO	0.45	0.45
Putative glycerophosphodiester phosphodiesterase 5	*FO*	0.53	0.45
	66VO	0.25	0.36
Putative hydroxypyruvate isomerase	*FO*	0.34	0.42
	66VO	0.29	0.41
Putative metalloprotease C21orf57	*FO*	0.66	0.63
	66VO	0.44	0.60
Putative N-acetylglucosamine-6-phosphate deacetylase	*FO*	0.73	0.55
	66VO	0.28	0.30
Putative uncharacterized protein C6orf191	*FO*	0.57	0.69
	66VO	0.22	0.48
Pyridine nucleotide-disulphide oxidoreductase domain 1	*FO*	0.90	0.88
	66VO	0.59	0.59
Pyridoxine-5'-phosphate oxidase	*FO*	0.64	0.80
	66VO	0.41	0.58
Pyrin	*FO*	0.89	0.88
	66VO	0.43	0.55
Quinone oxidoreductase	*FO*	0.59	0.49
	66VO	0.34	0.43
Rab GTPase-binding effector protein 2	*FO*	0.57	0.49
	66VO	0.26	0.35
Rab proteins geranylgeranyltransferase component A 1	*FO*	0.74	0.68
	66VO	0.48	0.46
RAD50-interacting protein 1	*FO*	0.66	0.66
	66VO	0.52	0.60
Ral guanine nucleotide dissociation stimulator-like 2	*FO*	0.56	0.45
	66VO	0.23	0.41
Ras and Rab interactor 2	*FO*	0.81	0.84
	66VO	0.61	0.64
Ras GTPase-activating protein 1	*FO*	0.70	0.76
	66VO	0.54	0.64
Ras-related GTP-binding protein C	*FO*	0.68	0.77
	66VO	0.38	0.45
Ras-related protein O-Krev	*FO*	0.71	0.83
	66VO	0.61	0.68
Ras-related protein Rab-2A	*FO*	0.64	0.61
	66VO	0.51	0.54
Ras-related protein Rab-7a	*FO*	0.75	0.94
	66VO	0.49	0.41
RB1-inducible coiled-coil protein 1	*FO*	0.65	0.66
	66VO	0.55	0.59
Receptor-transporting protein 3	*FO*	0.73	0.74
	66VO	0.32	0.31
Receptor-type tyrosine-protein phosphatase beta	*FO*	0.48	0.66
	66VO	0.30	0.47
Regulator of G-protein signaling 2	*FO*	0.54	0.56
	66VO	0.34	0.33
RelA-associated inhibitor	*FO*	0.72	0.67
	66VO	0.54	0.58
Renin receptor	*FO*	0.50	0.63
	66VO	0.42	0.39
Response gene to complement 32 protein	*FO*	0.69	0.55
	66VO	0.72	0.48
Reticulon-1	*FO*	0.41	0.35

	66VO	0.29	0.43
Retinal dehydrogenase 2	*FO*	0.63	0.64
	66VO	0.41	0.43
Retinoblastoma-binding protein 6	*FO*	0.61	0.59
	66VO	0.69	0.71
Retinoic acid receptor alpha	*FO*	0.69	0.72
	66VO	0.55	0.59
Retinoic acid receptor RXR-beta	*FO*	0.71	0.67
	66VO	0.52	0.60
Retinol dehydrogenase 7	*FO*	0.69	0.59
	66VO	0.35	0.48
Retrovirus-related Pol polyprotein from transposon opus	*FO*	0.73	0.83
	66VO	0.66	0.52
Retrovirus-related Pol polyprotein LINE-1	*FO*	0.65	0.66
	66VO	0.45	0.63
Rhesus blood group-associated glycoprotein	*FO*	0.65	0.59
	66VO	0.36	0.61
Rhomboid domain-containing protein 1	*FO*	0.78	0.66
	66VO	0.60	0.58
Rho-related GTP-binding protein RhoC	*FO*	0.79	0.71
	66VO	0.59	0.65
Rho-related GTP-binding protein RhoN	*FO*	0.46	0.55
	66VO	0.31	0.43
Ribonuclease inhibitor	*FO*	0.83	0.74
	66VO	0.32	0.43
Ribonuclease T2	*FO*	0.64	0.71
	66VO	0.56	0.53
Ribose-phosphate pyrophosphokinase 2	*FO*	0.59	0.50
	66VO	0.45	0.55
Ribosome biogenesis protein NSA2 homolog	*FO*	0.76	0.65
	66VO	0.46	0.55
RING finger protein 11	*FO*	0.58	0.58
	66VO	0.45	0.41
RING finger protein 212	*FO*	1.65	0.26
	66VO	0.79	0.85
RING finger protein 4	*FO*	0.80	0.83
	66VO	0.73	0.68
RMD5 homolog B	*FO*	0.69	0.65
	66VO	0.61	0.64
RNA 3'-terminal phosphate cyclase-like protein	*FO*	0.57	0.56
	66VO	0.60	0.62
RNA binding motif protein 10 like (kd	*FO*	0.66	0.60
	66VO	0.56	0.70
RNA pseudouridylate synthase domain-containing protein 1	*FO*	0.89	0.76
	66VO	0.48	0.60
RNA-binding motif, single-stranded-interacting protein 3	*FO*	0.69	0.61
	66VO	0.48	0.56
RNA-binding protein 10	*FO*	0.50	0.44
	66VO	0.32	0.35
RNA-binding protein 5	*FO*	0.69	0.62
	66VO	0.38	0.48
RNA-binding protein 7	*FO*	0.69	0.70
	66VO	0.61	0.69
RNA-binding protein PNO1	*FO*	0.66	0.63
	66VO	0.58	0.59
RNAse k	*FO*	0.57	0.67
	66VO	0.46	0.40
Secernin-2	*FO*	0.60	0.49
	66VO	0.36	0.52

Secretory calcium-binding phosphoprotein 5	*FO*	0.42	0.54
	66VO	0.35	0.49
Seizure 6-like protein	*FO*	0.67	0.66
	66VO	0.52	0.61
Selenium-binding protein 1	*FO*	0.60	0.58
	66VO	0.29	0.47
Semaphorin-4E	*FO*	0.99	1.23
	66VO	0.55	0.70
Senescence-associated protein	*FO*	0.30	0.41
	66VO	0.41	0.36
Sentrin-specific protease 5	*FO*	0.67	0.75
	66VO	0.82	0.83
Sentrin-specific protease 7	*FO*	0.59	0.52
	66VO	0.38	0.37
Sequestosome-1	*FO*	0.59	0.53
	66VO	0.42	0.46
Ser/Thr-rich protein T10 in DGCR region	*FO*	0.66	0.56
	66VO	0.46	0.53
Serine hydrolase-like protein	*FO*	0.78	0.63
	66VO	0.23	0.52
Serine incorporator 3	*FO*	0.50	0.60
	66VO	0.31	0.41
Serine proteinase	*FO*	0.94	1.31
	66VO	0.82	1.75
Serine/threonine-protein kinase TBK1	*FO*	0.89	0.91
	66VO	0.76	0.75
Serine/threonine-protein kinase tousled-like 1	*FO*	0.62	0.66
	66VO	0.51	0.58
Serine/threonine-protein phosphatase 2A catalytic subunit beta isoform	*FO*	0.60	0.59
	66VO	0.41	0.41
SH3 domain containing ring finger 1	*FO*	0.53	0.49
	66VO	0.37	0.40
SH3 domain-binding glutamic acid-rich-like protein 2	*FO*	0.55	0.48
	66VO	0.35	0.65
SH3 domain-binding protein 5-like	*FO*	0.65	0.65
	66VO	0.57	0.61
Shwachman-Bodian-Diamond syndrome protein homolog	*FO*	0.67	0.65
	66VO	0.62	0.61
Sialate O-acetylesterase	*FO*	0.61	0.82
	66VO	0.26	0.32
Signal peptide peptidase-like 2A	*FO*	0.56	0.70
	66VO	0.39	0.58
Signal peptide, CUB and EGF-like domain-containing protein 1	*FO*	0.67	0.58
	66VO	0.39	0.62
Signal recognition particle receptor subunit alpha	*FO*	0.70	0.76
	66VO	0.77	0.68
Signal recognition particle receptor subunit beta	*FO*	0.81	0.77
	66VO	1.52	0.90
Signal transducer and activator of transcription 3	*FO*	0.64	0.87
	66VO	0.84	0.91
Signal-induced proliferation-associated 1-like protein 2	*FO*	0.64	0.67
	66VO	0.43	0.34
Similar to Kazrin-A	*FO*	0.59	0.57
	66VO	0.48	0.61
Skeletal muscle and kidney-enriched inositol phosphatase	*FO*	0.70	0.64
	66VO	0.49	0.56
SLIT and NTRK-like protein 6	*FO*	0.51	0.46
	66VO	0.28	0.35
Small EDRK-rich factor 2	*FO*	0.72	0.60

	66VO	0.56	0.56
Small glutamine-rich tetratricopeptide repeat-containing protein B	*FO*	0.38	0.35
	66VO	0.28	0.36
Small muscular protein	*FO*	0.71	0.79
	66VO	0.44	0.55
Small nuclear RNA activating polypeptide 1b	*FO*	0.79	0.81
	66VO	0.74	0.77
Smoothelin-like protein 1	*FO*	0.97	1.03
	66VO	0.24	0.74
snRNA-activating protein complex subunit 3	*FO*	0.72	0.68
	66VO	0.66	0.74
Sodium- and chloride-dependent GABA transporter 3	*FO*	0.74	0.73
	66VO	0.47	0.59
Sodium channel modifier 1	*FO*	0.83	0.63
	66VO	0.34	0.53
Sodium channel protein type 4 subunit alpha	*FO*	0.42	0.37
	66VO	0.39	0.37
Sodium/potassium-transporting ATPase subunit alpha-1	*FO*	0.77	0.77
	66VO	0.71	0.78
Sodium-coupled neutral amino acid transporter 3	*FO*	0.62	0.84
	66VO	0.76	0.64
Soluble calcium-activated nucleotidase 1	*FO*	0.77	0.87
	66VO	0.44	0.66
Solute carrier family 12 member 7	*FO*	0.40	0.15
	66VO	0.52	0.36
Solute carrier family 12 member 8	*FO*	0.72	0.64
	66VO	0.35	0.64
Solute carrier family 2, facilitated glucose transporter member 5	*FO*	0.63	0.41
	66VO	0.35	0.42
Solute carrier family 25 member 43	*FO*	0.87	0.94
	66VO	0.58	0.59
Somatotropin	*FO*	0.32	0.42
	66VO	0.26	0.37
Sorting nexin-10	*FO*	0.50	0.52
	66VO	0.40	0.45
Sorting nexin-14	*FO*	0.39	0.32
	66VO	0.71	0.60
Sorting nexin-4	*FO*	0.69	0.69
	66VO	0.43	0.55
Sorting nexin-8	*FO*	0.46	0.59
	66VO	0.32	0.32
Sorting nexin-9	*FO*	0.62	0.66
	66VO	0.39	0.41
Soul heme-binding protein	*FO*	0.93	0.76
	66VO	0.59	0.58
SPARC	*FO*	0.63	0.72
	66VO	0.42	0.61
Spastin	*FO*	0.48	0.38
	66VO	0.82	0.75
Speckle-type POZ protein	*FO*	0.78	0.70
	66VO	0.72	0.57
Spectrin alpha chain, brain	*FO*	0.63	0.73
	66VO	0.50	0.58
Spectrin beta chain, brain 1	*FO*	0.65	0.75
	66VO	0.30	0.46
Sperm associated antigen 17	*FO*	0.36	0.36
	66VO	0.39	0.50
Splicing factor 3B subunit 1	*FO*	0.69	0.66
	66VO	0.33	0.40

Splicing factor arginine/serine-rich 11	*FO*	0.65	0.59
	66VO	0.63	0.70
Stabilin-2	*FO*	0.93	0.85
	66VO	0.73	0.41
STAM-binding protein-like	*FO*	0.58	0.54
	66VO	0.39	0.46
STE20-like serine/threonine-protein kinase	*FO*	0.81	0.65
	66VO	0.63	0.60
STIP1 homology and U box-containing protein 1	*FO*	0.58	0.53
	66VO	0.27	0.34
Stonustoxin subunit alpha	*FO*	0.81	0.80
	66VO	0.42	0.62
Stonustoxin subunit beta	*FO*	0.40	0.58
	66VO	0.30	0.49
Stromal cell-derived factor 1	*FO*	0.77	0.72
	66VO	0.41	0.65
Sulfatase-modifying factor 1	*FO*	0.75	0.81
	66VO	0.49	0.59
Sulfated glycoprotein 1	*FO*	0.49	0.55
	66VO	0.41	0.43
Suppressor of G2 allele of SKP1 homolog	*FO*	0.72	0.73
	66VO	0.63	0.65
Survival of motor neuron protein interacting protein 1	*FO*	0.56	0.63
	66VO	0.43	0.48
Synaptogyrin-2	*FO*	0.83	0.93
	66VO	0.56	0.62
Synaptonemal complex central element protein 1	*FO*	0.64	0.91
	66VO	1.42	0.65
Synaptopodin-2	*FO*	0.65	0.60
	66VO	0.33	0.55
Syntaxin-18	*FO*	0.63	0.66
	66VO	0.49	0.55
Syntaxin-binding protein 2	*FO*	0.72	0.78
	66VO	0.66	0.64
Target of Myb protein 1	*FO*	0.50	0.46
	66VO	0.61	0.69
TATA element modulatory factor	*FO*	0.59	0.63
	66VO	0.36	0.41
Tax1-binding protein 1 homolog	*FO*	0.59	0.71
	66VO	0.50	0.51
TBC1 domain family member 10A	*FO*	0.50	0.46
	66VO	0.32	0.39
TBC1 domain family member 13	*FO*	0.92	0.92
	66VO	0.63	0.61
Tcb1 transposase	*FO*	0.50	0.55
	66VO	0.66	0.62
T-cell immunomodulatory protein	*FO*	0.55	0.67
	66VO	0.57	0.62
T-cell receptor gamma chain c region	*FO*	0.77	0.92
	66VO	0.47	1.17
T-complex protein 1 subunit alpha	*FO*	1.40	1.59
	66VO	1.78	1.51
Telomerase protein component 1	*FO*	0.67	0.67
	66VO	0.44	0.57
Telomerase-binding protein EST1A	*FO*	0.76	0.62
	66VO	0.39	0.48
Telomeric repeat-binding factor 1	*FO*	0.63	0.59
	66VO	0.62	0.67
Telomeric repeat-binding factor 2	*FO*	0.79	0.57

	66VO	0.56	0.49
Telomeric repeat-binding factor 2-interacting protein 1	*FO*	0.72	0.72
	66VO	0.51	0.61
Tetraspanin-1	*FO*	0.95	1.25
	66VO	0.38	0.62
Tetraspanin-13	*FO*	0.54	0.58
	66VO	0.49	0.51
Tetraspanin-7	*FO*	0.70	0.59
	66VO	0.60	0.63
Tetratricopeptide repeat protein 35	*FO*	0.71	0.84
	66VO	0.74	0.69
THAP domain-containing protein 2	*FO*	0.75	0.89
	66VO	0.77	1.10
Thioredoxin reductase 1, cytoplasmic	*FO*	0.48	0.52
	66VO	0.26	0.31
THO complex subunit 5 homolog	*FO*	1.04	1.02
	66VO	1.44	1.29
Threonine dehydratase deaminase	*FO*	0.52	0.72
	66VO	0.36	0.54
Thymosin beta	*FO*	0.56	0.53
	66VO	0.39	0.57
Thyroid hormone receptor alpha	*FO*	0.79	0.75
	66VO	0.55	0.69
Thyrotropin subunit beta	*FO*	0.72	0.81
	66VO	0.41	0.65
Tissue alpha-L-fucosidase	*FO*	0.48	0.57
	66VO	0.20	0.32
Topoisomerase i	*FO*	1.02	1.50
	66VO	0.65	0.91
Torsin-1A	*FO*	0.80	0.78
	66VO	0.48	0.51
Tracheary element differentiation-related 7a	*FO*	0.59	0.54
	66VO	0.56	0.57
Trafficking protein particle complex subunit 4	*FO*	0.74	0.80
	66VO	0.74	0.72
Trafficking protein particle complex subunit 5	*FO*	0.65	0.83
	66VO	0.38	0.46
Transaldolase	*FO*	1.12	1.16
	66VO	1.46	1.30
Transcription elongation factor 1 homolog	*FO*	0.69	0.64
	66VO	0.48	0.46
Transcription elongation factor A protein 3	*FO*	0.80	0.68
	66VO	0.33	0.59
Transcription factor Adf-1	*FO*	0.80	0.81
	66VO	0.78	0.79
Transcription factor MafB	*FO*	0.44	0.66
	66VO	0.45	0.46
Transcription initiation factor TFIID subunit 1	*FO*	0.79	0.89
	66VO	0.78	0.77
Transcriptional activator protein Pur-alpha	*FO*	0.58	0.57
	66VO	0.56	0.67
Transcriptional enhancer factor TEF-1	*FO*	0.58	0.65
	66VO	0.51	0.58
Transcriptional repressor NF-X1	*FO*	0.66	0.69
	66VO	0.47	0.52
Transcriptional repressor protein YY1	*FO*	0.78	0.81
	66VO	0.74	0.78
Transforming protein RhoA	*FO*	0.64	0.81
	66VO	0.45	0.75

Transgelin	*FO*	0.93	0.99
	66VO	0.42	0.79
Translocator protein	*FO*	0.62	0.92
	66VO	0.54	0.65
Translocon-associated protein subunit beta	*FO*	1.23	1.24
	66VO	1.43	0.92
Transmembrane and TPR repeat-containing protein 4	*FO*	0.82	0.82
	66VO	0.51	0.72
Transmembrane gamma-carboxyglutamic acid protein 4	*FO*	0.69	0.80
	66VO	0.49	0.60
Transmembrane protein 11	*FO*	0.50	0.53
	66VO	0.37	0.39
Transmembrane protein 120A	*FO*	0.89	0.95
	66VO	1.52	1.30
Transmembrane protein 127	*FO*	0.67	0.76
	66VO	0.60	0.67
Transmembrane protein 131	*FO*	0.71	0.78
	66VO	0.68	0.78
Transmembrane protein 134	*FO*	0.52	0.57
	66VO	0.34	0.45
Transmembrane protein 14A	*FO*	0.62	0.70
	66VO	0.47	0.56
Transmembrane protein 184B	*FO*	0.64	0.58
	66VO	0.25	0.45
Transmembrane protein 199	*FO*	0.72	0.78
	66VO	0.59	0.64
Transmembrane protein 47	*FO*	0.81	0.97
	66VO	0.31	0.77
Transmembrane protein 50A	*FO*	0.62	0.61
	66VO	0.61	0.60
Transmembrane protein 8	*FO*	0.64	0.66
	66VO	0.34	0.45
Transmembrane protein HSPC244	*FO*	0.72	0.82
	66VO	0.48	0.50
Transposable element Tcb1 transposase	*FO*	0.68	0.80
	66VO	0.65	0.72
Transposable element Tcb2 transposase	*FO*	0.65	0.64
	66VO	0.33	0.42
Trimeric intracellular cation channel type A	*FO*	0.63	0.60
	66VO	0.29	0.64
Tripartite motif-containing containing 25	*FO*	0.72	0.71
	66VO	0.42	0.54
Tripartite motif-containing protein 16	*FO*	0.65	0.68
	66VO	0.50	0.60
Tripartite motif-containing protein 25	*FO*	0.74	0.65
	66VO	0.40	0.47
Tripartite motif-containing protein 39	*FO*	0.86	0.78
	66VO	0.54	0.51
Tripartite motif-containing protein 69	*FO*	0.76	0.72
	66VO	0.52	0.65
tRNA guanosine-2'-O-methyltransferase TRM11 homolog	*FO*	1.00	0.54
	66VO	0.39	0.52
Tropomodulin-2	*FO*	0.85	0.62
	66VO	0.53	0.58
Tropomodulin-4	*FO*	1.32	1.53
	66VO	0.38	0.89
Tropomyosin alpha-3 chain	*FO*	0.71	0.71
	66VO	0.65	0.64
Tropomyosin alpha-4 chain	*FO*	0.74	0.76

	66VO	0.34	0.57
Tropomyosin-1 alpha chain	*FO*	1.39	1.36
	66VO	0.33	1.52
Troponin I, fast skeletal muscle	*FO*	0.60	0.29
	66VO	0.35	0.36
Troponin T, cardiac muscle isoforms	*FO*	0.63	0.71
	66VO	0.27	0.66
Trpc4-associated protein	*FO*	0.81	0.67
	66VO	0.67	0.66
Tryptase-2	*FO*	0.42	1.67
	66VO	0.46	2.43
Tuberin	*FO*	0.63	0.66
	66VO	0.41	0.50
Tubulin alpha-1 chain	*FO*	0.53	0.35
	66VO	0.21	0.42
Tubulin polyglutamylase complex subunit 2	*FO*	0.58	0.61
	66VO	0.44	0.27
Tubulin-specific chaperone A	*FO*	0.70	0.74
	66VO	0.79	0.70
Tumor necrosis factor receptor superfamily member 14	*FO*	0.61	0.74
	66VO	0.55	0.71
Tumor necrosis factor receptor superfamily member 16	*FO*	0.50	0.54
	66VO	0.32	0.59
Tumor suppressor candidate 2	*FO*	0.57	0.55
	66VO	0.55	0.61
Type II cax cation proton exchanger	*FO*	0.17	0.31
	66VO	0.73	0.79
Tyrosine aminotransferase	*FO*	0.77	0.75
	66VO	0.68	0.71
U2-associated protein SR140	*FO*	0.68	0.73
	66VO	0.45	0.64
U3 small nucleolar RNA-associated protein 13	*FO*	0.80	0.80
	66VO	0.74	0.69
Ubiquinone biosynthesis protein COQ9, mitochondrial	*FO*	0.69	0.64
	66VO	0.46	0.48
Ubiquitin	*FO*	0.75	0.67
	66VO	0.64	0.61
Ubiquitin carboxyl-terminal hydrolase 25	*FO*	0.64	0.60
	66VO	0.53	0.59
Ubiquitin carboxyl-terminal hydrolase 45	*FO*	0.71	0.52
	66VO	0.42	0.57
Ubiquitin carboxyl-terminal hydrolase 48	*FO*	0.57	0.67
	66VO	0.78	0.87
Ubiquitin carboxyl-terminal hydrolase isozyme L1	*FO*	0.40	0.34
	66VO	0.34	0.47
Ubiquitin fusion degradation protein 1 homolog	*FO*	0.79	0.84
	66VO	0.74	0.69
Ubiquitin thioesterase OTUB1	*FO*	0.64	0.67
	66VO	0.48	0.52
Ubiquitin thioesterase OTUB2	*FO*	0.63	0.54
	66VO	0.42	0.49
Ubiquitin-conjugating enzyme E2 A	*FO*	0.70	0.77
	66VO	0.42	0.40
Ubiquitin-conjugating enzyme E2 D3	*FO*	0.67	0.63
	66VO	0.54	0.47
Ubiquitin-conjugating enzyme E2 D4	*FO*	0.78	0.71
	66VO	0.57	0.62
Ubiquitin-conjugating enzyme E2 E1	*FO*	0.71	0.71
	66VO	0.58	0.54

Ubiquitin-conjugating enzyme E2 G2	*FO*	0.70	0.67
	66VO	0.82	0.73
Ubiquitin-conjugating enzyme E2-25 kDa	*FO*	0.63	0.56
	66VO	0.61	0.40
Ubiquitin-related modifier 1 homolog	*FO*	0.70	0.73
	66VO	0.48	0.54
UBX domain-containing protein 2	*FO*	0.80	0.83
	66VO	0.69	0.71
UBX domain-containing protein 8	*FO*	0.70	0.77
	66VO	0.85	0.72
UDP-GlcNAc:betaGal beta-1,3-N-acetylglucosaminyltransferase 5A	*FO*	0.57	1.03
	66VO	0.42	0.47
UDP-glucuronosyltransferase 1-1	*FO*	0.76	0.94
	66VO	0.19	0.41
UDP-glucuronosyltransferase 1-4	*FO*	0.37	0.62
	66VO	0.71	0.89
UDP-glucuronosyltransferase 2B31	*FO*	0.56	0.60
	66VO	0.37	0.47
UDP-N-acetylglucosamine--peptide N-acetylglucosaminyltransferase 110	*FO*	0.73	0.70
	66VO	0.58	0.65
UNC93-like protein MFSD11	*FO*	0.56	0.56
	66VO	0.30	0.31
Uncharacterized protein C03B1.10	*FO*	0.55	0.56
	66VO	0.59	0.64
Uncharacterized protein C1039.06	*FO*	0.59	0.83
	66VO	0.31	0.48
Uncharacterized protein C10orf18 homolog	*FO*	0.61	0.57
	66VO	0.44	0.48
Uncharacterized protein C10orf57 homolog	*FO*	0.67	0.64
	66VO	0.58	0.55
Uncharacterized protein C11orf68	*FO*	0.76	0.74
	66VO	0.41	0.54
Uncharacterized protein C13orf33 homolog	*FO*	0.52	0.78
	66VO	0.41	0.50
Uncharacterized protein C14orf133	*FO*	0.72	0.68
	66VO	0.52	0.50
Uncharacterized protein C14orf135	*FO*	0.73	0.78
	66VO	0.67	0.76
Uncharacterized protein C14orf153	*FO*	0.66	0.83
	66VO	0.57	0.70
Uncharacterized protein C16orf14	*FO*	0.73	0.71
	66VO	0.56	0.62
Uncharacterized protein C17orf63 homolog	*FO*	0.63	0.50
	66VO	0.72	0.67
Uncharacterized protein C19orf61 homolog	*FO*	0.88	0.96
	66VO	1.25	1.08
Uncharacterized protein C1orf119 homolog	*FO*	0.78	0.82
	66VO	0.90	0.84
Uncharacterized protein C1orf160	*FO*	0.74	0.72
	66VO	0.56	0.61
Uncharacterized protein C1orf21 homolog	*FO*	0.55	0.55
	66VO	0.55	0.57
Uncharacterized protein C2orf25 homolog, mitochondrial	*FO*	1.09	0.98
	66VO	0.81	0.58
Uncharacterized protein C3orf32	*FO*	0.63	0.70
	66VO	0.59	0.58
Uncharacterized protein C61.03	*FO*	0.82	1.10
	66VO	0.62	0.62
Uncharacterized protein C6orf106 homolog	*FO*	0.82	0.78

	66VO	0.64	0.61
Uncharacterized protein C6orf130 homolog	*FO*	0.71	0.66
	66VO	0.46	0.55
Uncharacterized protein C6orf166	*FO*	0.74	0.68
	66VO	0.74	0.67
Uncharacterized protein C8orf40 homolog	*FO*	0.63	0.63
	66VO	0.49	0.53
Uncharacterized protein C9orf41	*FO*	0.60	0.64
	66VO	0.62	0.69
Uncharacterized protein C9orf82 homolog	*FO*	0.74	0.81
	66VO	0.53	0.75
Uncharacterized protein C9orf85 homolog	*FO*	0.62	0.78
	66VO	0.77	0.88
Uncharacterized protein C9orf97 homolog	*FO*	0.66	0.75
	66VO	0.62	0.65
Uncharacterized protein CXorf57	*FO*	0.71	0.68
	66VO	0.69	0.68
Uncharacterized protein DKFZP564O0523	*FO*	0.65	0.62
	66VO	0.62	0.56
Uncharacterized protein KIAA0652	*FO*	0.59	0.53
	66VO	0.58	0.51
Uncharacterized protein KIAA0892	*FO*	0.55	0.52
	66VO	0.41	0.44
Uncharacterized protein KIAA1370 homolog	*FO*	0.74	0.68
	66VO	0.35	0.37
Uncharacterized protein KIAA2026	*FO*	0.46	0.40
	66VO	0.46	0.40
Uncharacterized protein ZK1236.4	*FO*	0.42	0.60
	66VO	0.47	0.51
Uncharacterized serpin-like protein MA_2246	*FO*	0.70	0.83
	66VO	0.45	0.54
UPF0184 protein C9orf16 homolog	*FO*	0.47	0.57
	66VO	0.33	0.21
UPF0279 protein C14orf129 homolog	*FO*	0.71	0.76
	66VO	0.47	0.57
UPF0317 protein C14orf159, mitochondrial	*FO*	0.68	0.66
	66VO	0.42	0.55
UPF0402 protein	*FO*	0.69	0.71
	66VO	0.48	0.58
UPF0414 transmembrane protein C20orf30 homolog	*FO*	0.58	0.57
	66VO	0.47	0.62
UPF0448 protein C10orf104 homolog	*FO*	0.88	0.90
	66VO	0.74	0.65
UPF0451 protein C17orf61 homolog	*FO*	0.67	0.62
	66VO	0.46	0.50
UPF0453 protein C12orf44 homolog	*FO*	0.47	0.45
	66VO	0.30	0.36
UPF0454 protein C12orf49 homolog	*FO*	0.80	0.75
	66VO	0.46	0.47
UPF0459 protein C19orf50 homolog	*FO*	0.69	0.77
	66VO	0.50	0.54
UPF0468 protein C16orf80 homolog	*FO*	0.81	0.76
	66VO	0.77	0.79
UPF0480 protein C15orf24 homolog	*FO*	0.63	0.72
	66VO	0.53	0.54
UPF0487 protein C17orf71	*FO*	0.40	0.35
	66VO	0.46	0.45
UPF0493 protein KIAA1632 homolog	*FO*	0.60	0.57
	66VO	0.32	0.46

UPF0498 protein KIAA1191 homolog	*FO*	0.62	0.55
	66VO	0.44	0.53
UPF0518 protein C11orf56 homolog	*FO*	0.65	0.68
	66VO	0.49	0.72
Upstream stimulatory factor 2	*FO*	0.77	0.83
	66VO	0.61	0.78
Uroporphyrinogen-III synthase	*FO*	0.80	0.83
	66VO	0.61	0.72
Vacuolar ATP synthase 16 kDa proteolipid subunit	*FO*	0.60	0.65
	66VO	0.59	0.58
Vacuolar ATP synthase subunit S1	*FO*	0.42	0.56
	66VO	0.39	0.36
Vacuolar protein sorting-associated protein 11 homolog	*FO*	0.62	0.53
	66VO	0.52	0.58
Vacuolar protein sorting-associated protein 13B	*FO*	0.71	0.70
	66VO	0.59	0.62
Vacuolar protein sorting-associated protein 18 homolog	*FO*	0.72	0.74
	66VO	0.51	0.59
Vacuolar protein sorting-associated protein 33A	*FO*	0.63	0.67
	66VO	0.49	0.58
Vacuolar protein sorting-associated protein 8 homolog	*FO*	0.59	0.62
	66VO	0.42	0.44
Vacuolar protein-sorting-associated protein 36	*FO*	0.61	0.67
	66VO	0.55	0.69
Vacuolar proton pump subunit C 1	*FO*	0.68	0.69
	66VO	0.44	0.43
Vacuolar proton pump subunit d 1	*FO*	0.81	0.95
	66VO	0.51	0.55
Vacuolar proton pump subunit H	*FO*	0.63	0.70
	66VO	0.55	0.52
Vascular endothelial growth factor A	*FO*	0.59	0.53
	66VO	0.41	0.49
Vascular endothelial growth factor receptor 1	*FO*	0.64	0.59
	66VO	0.62	0.56
Vascular endothelial growth factor receptor 2 homolog B	*FO*	0.59	0.48
	66VO	0.54	0.46
Vascular endothelial zinc finger 1	*FO*	0.71	0.67
	66VO	0.74	0.64
Very long-chain acyl-CoA synthetase	*FO*	0.82	0.46
	66VO	0.16	0.21
Very low-density lipoprotein receptor	*FO*	0.56	0.77
	66VO	0.30	0.46
Vesicle transport protein SEC20	*FO*	0.79	0.71
	66VO	0.71	0.67
Vesicle transport protein SFT2A	*FO*	0.64	0.69
	66VO	0.59	0.62
Vesicle transport protein SFT2B	*FO*	0.75	0.85
	66VO	0.40	0.52
Vesicle-associated membrane protein 8	*FO*	0.49	0.47
	66VO	0.24	0.42
Vigilin	*FO*	0.71	0.61
	66VO	0.57	0.58
Vitamin K-dependent protein S	*FO*	0.53	0.57
	66VO	0.42	0.37
WD repeat and FYVE domain-containing protein 3	*FO*	0.57	0.59
	66VO	0.28	0.40
WD repeat domain phosphoinositide-interacting protein 1	*FO*	0.71	0.61
	66VO	0.30	0.43
WD repeat domain phosphoinositide-interacting protein 4	*FO*	0.63	0.63

	66VO	0.51	0.50
WD repeat, SAM and U-box domain-containing protein 1	*FO*	0.55	0.57
	66VO	0.25	0.33
WD repeat-containing protein 23	*FO*	0.66	0.65
	66VO	0.45	0.54
WD repeat-containing protein 44	*FO*	0.78	0.78
	66VO	0.61	0.68
WD repeat-containing protein 68	*FO*	0.66	0.70
	66VO	0.64	0.68
WD repeat-containing protein 82	*FO*	0.68	0.64
	66VO	0.53	0.77
WW domain-binding protein 2	*FO*	0.77	0.83
	66VO	0.76	0.77
Zbed1 protein	*FO*	0.83	0.74
	66VO	0.45	0.72
Zinc finger and KRAB domain-containing protein FLJ16287	*FO*	0.63	0.63
	66VO	0.46	0.56
Zinc finger CCCH domain-containing protein 10	*FO*	0.61	0.57
	66VO	0.27	0.37
Zinc finger ccch type containing 11a-like	*FO*	0.62	0.65
	66VO	0.58	0.63
Zinc finger CCHC domain-containing protein 6	*FO*	0.65	0.50
	66VO	0.33	0.51
Zinc finger domain containing protein	*FO*	0.60	0.53
	66VO	0.40	0.47
Zinc finger MYM-type protein 4	*FO*	0.72	0.74
	66VO	0.46	0.70
Zinc finger protein 227	*FO*	0.81	0.71
	66VO	0.51	0.65
Zinc finger protein 228	*FO*	0.75	0.73
	66VO	0.79	0.78
Zinc finger protein 235	*FO*	0.65	0.66
	66VO	0.45	0.54
Zinc finger protein 26	*FO*	0.69	0.87
	66VO	0.72	0.75
Zinc finger protein 287	*FO*	0.72	0.67
	66VO	0.60	0.67
Zinc finger protein 414	*FO*	0.77	0.67
	66VO	0.71	0.65
Zinc finger protein 43	*FO*	0.64	0.56
	66VO	0.40	0.51
Zinc finger protein 451	*FO*	0.70	0.69
	66VO	0.25	0.27
Zinc finger protein 518A	*FO*	0.63	0.63
	66VO	0.53	0.51
Zinc finger protein 675	*FO*	0.80	0.95
	66VO	0.48	0.83
Zinc finger protein RFP	*FO*	0.85	0.99
	66VO	0.49	0.44
Zinc finger protein ubi-d4	*FO*	0.78	0.80
	66VO	0.76	0.81
Zinc transporter ZIP13	*FO*	0.74	0.72
	66VO	0.50	0.70
Zinc-binding protein A33	*FO*	0.61	0.65
	66VO	0.29	0.31
Zona pellucida sperm-binding protein 4	*FO*	0.24	0.31
	66VO	0.80	1.09
