# Supplementary material for: Dietary vegetable oils do not alter the intestine transcriptome of gilthead sea bream (Sparus aurata), but modulate the transcriptomic response to infection with Enteromyxum leei
Source: BMC Genomics. 2012 Sep 11;13:470. doi: 10.1186/1471-2164-13-470 (PMC3444936; doi:10.1186/1471-2164-13-470)
Supplement: Additional file 2 — Table S2. Ingredients and chemical composition of experimental diets. [file 1471-2164-13-470-S2.pdf]

Ingredient (%)	FO	66VO
Fish meal (CP 70%) [1]	15	15
CPSP 90 [2]	5	5
Corn gluten	40	40
Soybean meal	14.3	14.3
Extruded wheat	4	4
Fish oil [3]	15.15	5.15
Rapeseed oil	0	1.7
Linseed oil	0	5.8
Palm oil	0	2.5
Soya lecithin	1	1
Binder	1	1
Mineral premix [4]	1	1
Vitamin premix [5]	1	1
$CaHPO_4.2H_2O$ (18%P)	2	2
L-Lys	0.55	0.55
Proximate composition		
Dry matter (DM, %)	93.13	92.77
Protein (% DM)	53.2	52.62
Fat (% DM)	21.09	20.99
Ash (% DM)	6.52	6.57

[1]Fish meal (Scandinavian LT)
[2]Fish soluble protein concentrate (Sopropêche, France)
[3]Fish oil (Sopropêche, France)
[4]Supplied the following (mg / kg diet, except as noted): calcium carbonate (40% Ca) 2.15 g, magnesium hydroxide (60% Mg) 1.24 g, potassium chloride 0.9 g, ferric citrate 0.2 g, potassium iodine 4 mg, sodium chloride 0.4 g, calcium hydrogen phosphate 50 g, copper sulphate 0.3, zinc sulphate 40, cobalt sulphate 2, manganese sulphate 30, sodium selenite 0.3.
[5]Supplied the following (mg / kg diet): retinyl acetate 2.58, DL-cholecalciferol 0.037, DL-α tocopheryl acetate 30, menadione sodium bisulphite 2.5, thiamin 7.5, riboflavin 15, pyridoxine 7.5, nicotinic acid 87.5, folic acid 2.5, calcium pantothenate 2.5, vitamin B_{12} 0.025, ascorbic acid 250, inositol 500, biotin 1.25 and choline chloride 500.
