# Supplementary material for: Dietary vegetable oils do not alter the intestine transcriptome of gilthead sea bream (Sparus aurata), but modulate the transcriptomic response to infection with Enteromyxum leei
Source: BMC Genomics. 2012 Sep 11;13:470. doi: 10.1186/1471-2164-13-470 (PMC3444936; doi:10.1186/1471-2164-13-470)
Supplement: Additional file 3 — Table S3. Top biological functions (GO multilevel) represented on the gilthead sea bream oligo-microarray. [file 1471-2164-13-470-S3.pdf]

Gene Ontology: biological process (multilevel)

GO description	Features
Response to stress	562
Organ development	560
Proteolysis	499
Negative regulation of cellular process	492
Positive regulation of cellular process	486
Organelle organization	461
Cell differentiation	432
Response to chemical stimulus	426
Post-translational protein modification	413
Regulation of transcription, DNA-dependent	408
Signal transmission	402
Anatomical structure morphogenesis	382
Oxidation reduction	372
Cell cycle	364
Lipid metabolic process	344
Intracellular signalling pathway	342
Phosphorylation	330
Macromolecular complex assembly	310
Translation	308
Protein transport	306
Cell proliferation	302
Carboxylic acid metabolic process	300
Apoptosis	299
Immune system process	297
Intracellular transport	292
Cellular macromolecule catabolic process	288
Small molecule biosynthetic process	288
Regulation of molecular function	276
Homeostatic process	275
Cell surface receptor linked signalling pathway	274
